# Organelle-dependent polyprotein designs enable stoichiometric expression of nitrogen fixation components targeted to mitochondria

**DOI:** 10.1073/pnas.2305142120

**Published:** 2023-08-16

**Authors:** Jianguo Yang, Nan Xiang, Yiheng Liu, Chenyue Guo, Chenyu Li, Hui Li, Shuyi Cai, Ray Dixon, Yi-Ping Wang

**Affiliations:** ^a^State Key Laboratory of Protein and Plant Gene Research, School of Advanced Agricultural Sciences and School of Life Sciences, Peking University, Beijing 100871, China; ^b^Department of Molecular Microbiology, John Innes Centre, NR4 7UH Norwich, United Kingdom

**Keywords:** nitrogenase, polyprotein, mitochondrial-processing peptidase, synthetic biology

## Abstract

Introducing nitrogen fixation (*nif*   ) genes into eukaryotic genomes and targeting Nif components to mitochondria or chloroplasts is a promising strategy for engineering nitrogen-fixing plants. A prerequisite for achieving nitrogen fixation in crops is stable and stoichiometric expression of each component in organelles. Previously, we designed a polyprotein-based nitrogenase system depending on Tobacco Etch Virus protease (TEVp) to release functional Nif components from five polyproteins. Although this system satisfies the demand for specific expression ratios of Nif components in *Escherichia coli*, we encountered issues with TEVp cleavage of polyproteins targeted to yeast mitochondria. To overcome this obstacle, a version of the Nif polyprotein system was constructed by replacing TEVp cleavage sites with minimal peptide sequences, identified by knowledge-based engineering, that are susceptible to cleavage by the endogenous mitochondrial-processing peptidase. This replacement not only further reduces the number of genes required, but also prevents potential precleavage of polyproteins outside the target organelle. This version of the polyprotein-based nitrogenase system achieved levels of nitrogenase activity in *E. coli*, comparable to those observed with the TEVp-based polyprotein nitrogenase system. When applied to yeast mitochondria, stable and balanced expression of Nif components was realized. This strategy has potential advantages, not only for transferring nitrogen fixation to eukaryotic cells, but also for the engineering of other metabolic pathways that require mitochondrial compartmentalization.

## Significance

Engineering the capacity for biological nitrogen fixation in cereals would have tremendous environmental and socioeconomic benefits, since this process provides a more sustainable alternative to the application of chemical fertilizers. The physiological environment and compartmentalized structure of mitochondria provide a unique location for metabolic pathway reconstitution, including nitrogen fixation. In the current study, we redesigned a polyprotein system linked by engineered peptides cleaved by the intrinsic mitochondrial-processing peptidase that enables stable expression of protein components required for nitrogenase biosynthesis in yeast mitochondria. In addition to engineering nitrogen fixation, we demonstrate that this polyprotein approach expands the potential for utilizing mitochondria as a biotechnology platform through functional reconstitution of the violacein and isobutanol pathways in this organelle.

Nitrogen availability is a critical factor for plant growth. Over the past century, agricultural yield increases, particularly for cereal crops, have been mainly dependent on the use of industrial nitrogen fertilizers, which have contributed to food provision for about half of the world’s population (1). However, the overuse of nitrogen fertilizers results in heavy economic and environmental penalties (2, 3). The conversion of atmospheric abundant dinitrogen gas into bioavailable ammonia occurs naturally in diverse prokaryotic diazotrophs. This process called biological nitrogen fixation, depends on O_2_–sensitive nitrogenase enzymes (4). The most widely studied molybdenum nitrogenase is a complex metalloprotein consisting of two components, the Fe protein and the MoFe protein. Fe protein is a homodimer encoded by the gene *nifH*, which in addition to its function as an obligate electron donor to the MoFe protein, carries out multiple roles in nitrogenase maturation and cofactor biosynthesis (5). Substrate reduction is carried out by the heterotetrameric MoFe protein encoded by the genes *nifD* and *nifK*. During nitrogenase turnover, different types of metalloclusters in these proteins play a pivotal role in electron transfer and active site catalysis to break the triple bond in dinitrogen. These metal clusters include the [4Fe-4S] cluster bridging the two NifH subunits, the P-cluster located at the interface of NifD and NifK, and the FeMo cofactor (FeMo-co) liganded to NifD that forms the active site of nitrogenase. Many Nif components contribute to the biosynthesis of these complex metal clusters and their insertion into nitrogenase apoproteins (6, 7). The expression of the structural subunits, metal cluster biosynthesis and electron transport components are exquisitely regulated to meet specific temporal and stoichiometric requirements, which are essential for assembly of the active form of nitrogenase to accomplish nitrogen fixation (8).

To reduce the requirement for chemical fertilizers, there is considerable focus on engineering nitrogen-fixing crops that can use dinitrogen gas to support growth, through direct introduction of *nif* genes into the plant genome. Both mitochondria and chloroplasts as energy generating organelles have been suggested to provide intracellular locations for engineering functional nitrogenase in eukaryotes to support the high energy requirement for N_2_ reduction and the necessity to protect nitrogenase from oxygen inactivation (9). Direct proof of concept for oxygen protection of nitrogenase in yeast mitochondria has been demonstrated by the observation that Fe protein, the most oxygen sensitive component of the enzyme, can be extracted in an active form from aerobically grown yeast cells (10). Fe proteins have also been purified from plant mitochondria or chloroplasts although not replete with a [4Fe-4S] cluster (11, 12). Biosynthesis of functional nitrogenase MoFe protein is far more complex, since it requires assembly of two metalloclusters: the highly complex active site cofactor FeMo-co [7Fe-9S-C-Mo-homocitrate] and the P cluster [8Fe-7S], combined with their interactions with the structural subunits NifD and NifK and additional maturation factors to form the holoenzyme (13). At least eight *nif* genes are required for stepwise assembly of FeMo-co and insertion of this cofactor into apo-MoFe protein (NifDK) to form the catalytic site of the enzyme (6, 7). Among these *nif* gene products, NifB is a key component responsible for generating a [8Fe-9S-C] cluster called NifB-co, which is the core component of FeMo-co (14). Coexpression of components required for the maturation of NifB-co, including *Azotobacter vinelandii* NifU, NifS, NifX, FdxN together with NifB from the archaea *M. thermautotrophicus*, resulted in the biosynthesis of active NifB-co in vivo, when these proteins were targeted to yeast mitochondria, a crucial step toward synthesis of complete FeMo-co (15). Although the NifDK tetramer has been detected in yeast mitochondria (16), assembly of active MoFe protein replete with metalloclusters has not yet been achieved. One of the issues hindering the achievement of this goal is the apparent instability of the NifD subunit in both yeast and plant mitochondria (16, 17). Further analysis demonstrated that an internal sequence of the NifD protein is accessible to the mitochondrial-processing peptidase (MPP), which is responsible for removing the N-terminal presequences of proteins targeted to mitochondria (18, 19). The instability of NifD can be circumvented by using NifD variants resistant to degradation by MPP that also maintain catalytic activity (17, 19).

Stable and balanced expression of each Nif component is a prerequisite for the biosynthesis of functional nitrogenase. However, it is difficult to achieve the appropriate protein stoichiometry when engineering nitrogen fixation in eukaryotic cells, since the expression output from individual refactored genes is less predictable in eukaryotes (16, 20). To facilitate stochiometric gene expression, we designed a “polyprotein” strategy to combine and rearrange *nif* genes into single “giant” translational units that were subject to posttranslational splicing by Tobacco Etch Virus protease (TEVp), thus releasing the individual protein components (21). The entire *nif* cluster from *Klebsiella oxytoca* was successfully reconstituted into five giant genes, encoding a polyprotein system in *Escherichia coli* that exhibited nitrogenase activity and diazotrophic growth after cleavage with TEVp (21).

Although the TEVp-based Nif polyprotein system reduces the number of translational units and therefore has significant potential for balanced gene expression in eukaryotes, we encountered issues in applying this system to target proteins to yeast mitochondria, since the stability of the polyproteins was dramatically reduced. We also observed that MBP-TEVp is toxic to yeast when overexpressed with polyproteins targeted to mitochondria. To bypass these limitations, we engineered a version of the polyprotein-based nitrogenase system, which is dependent on the MPP for endogenous polyprotein cleavage. By systematically investigating and screening requirements for MPP cleavage, we have identified a 10 residues peptide that can replace the TEVp recognition site in polyproteins to provides efficient internal cleavage by MPP. This has enabled us to engineer a MPP-based Nif polyprotein system in *E. coli* that supports diazotrophic growth. When this system is refactored to support expression and targeting of polyproteins to yeast mitochondria, efficient cleavage by MPP occurs in the mitochondrial matrix, providing release of individual Nif component proteins in stochiometric amounts. As further proof of principle, we have also engineered MPP-based polyprotein systems that introduce biosynthetic pathways for isobutanol and violacein biosynthesis in yeast mitochondria.

## Results

### Testing the TEVp-Based Polyprotein System in Yeast Mitochondria.

**Fig. 1.**
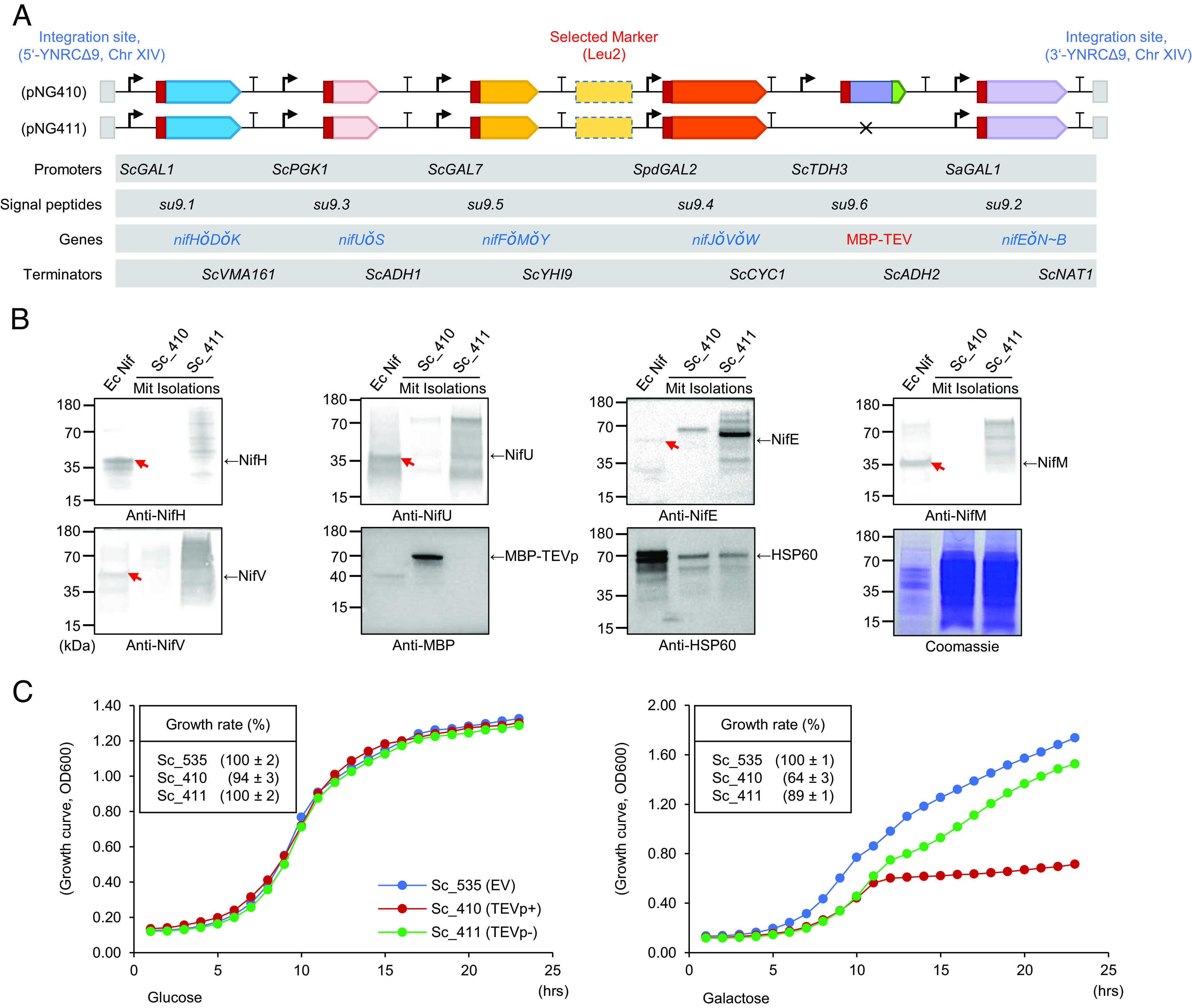
Engineering and assessment of the TEVp-based Nif polyprotein system in yeast mitochondria. (*A*) Schematic diagram of the TEVp-based *nif* constructs for expression in *S. cerevisiae*. Giant genes are highlighted in red color and symbol “ǒ” is used to represent dual TEVp sites (ENLYFQSENLYFQS). The selected marker Leu2 (indicated above in red) is used for auxotrophic selection of transformants. Constructs were integrated in the YNRCΔ9 locus on chromosome XIV of the yeast genome with ~600 bp 5′YNRCΔ9 and 3′YNRCΔ9 flanking regions as homology arms (Dataset S1). (*B*) Immunoblotting of Nif proteins from yeast carrying the TEVp-based Nif polyprotein system. Ec Nif, indicates protein samples prepared from *E. coli* cells carrying the reconstituted operon-based *nif* system (24). Mit Isolations, indicates protein samples prepared from mitochondrial extracts. HSP60 was used as an internal reference and a Coomassie blue stained PAGE gel was used as control for equal loading of each sample. Long-term exposure of these images is provided in *SI Appendix*, Fig. S3. (*C*) Growth curves of yeast strains carrying the TEVp-based Nif polyprotein system. Sc_535, is a yeast strain transformed with empty vector carrying the Leu2 selection marker (EV) assigned as a control. Sc_410 and Sc_411, are yeast strains carrying constructs pNG410 and pNG411 respectively. “Growth rate” represents the relative maximum growth rate of each strain. The maximum growth rate of strain Sc_535 was assigned as 100%. In the panel on the left, glucose was initially present at 2%. For the right hand-panel, 0.4% of glucose was used for pregrowth, and the final ratio of glucose and galactose was 1:4.

In our previous study, we established a polyprotein strategy in which the *K. oxytoca nif* genes were regrouped into five giant genes whose products were expressed and subsequently cleaved by tobacco etch virus protease (TEVp). This polyprotein system enabled nitrogenase activity and supported diazotrophic growth of *E. coli* after polyprotein cleavage by TEVp (21). To follow up on this strategy, we designed *nif-*encoded polyproteins for expression in yeast, each flanked by different promoters and terminators, with variant sequences encoding the signal peptide of subunit 9 (Su9) of the *Neurospora crassa* ATP (Adenosine triphosphate) synthase (F0-ATPase) fused to the 5′ end of the giant *nif* genes to enable import of polyproteins into yeast mitochondria. Constructs were finally assembled with or without sequences designed to express and target a MBP-TEVp fusion protein to mitochondria (plasmids pNG410 and pNG411 respectively), which were subsequently transformed and integrated into chromosome XIV of *Saccharomyces cerevisiae* at the YNRCΔ9 locus to generate stable strains Sc_410 and Sc_411 (Fig. 1*A*). However, when mitochondria extracts were prepared from aerobically grown yeast cultures and analyzed by immunoblotting using antibodies against specific Nif proteins, negligible amounts of Nif components were detected in strain Sc_410, which contains the complete TEVp-based *nif* system, even though MBP-TEVp was clearly imported into mitochondria (Fig. 1*B* and *SI Appendix*, Fig. S3). This could imply that polyproteins are not imported into mitochondria, potentially because they are unstable in the cytoplasm, or once imported into the mitochondrial matrix they are rapidly degraded. In contrast, cross-reaction to the antibodies was notable in extracts from strain Sc_411, which does not encode TEVp, suggesting that polyprotein import may have occurred, but the smearing of bands on the gel suggests they are unstable in mitochondria in the absence of processing, perhaps indicative of misfolded proteins being targeted for degradation (Fig. 1*B*). Although no substantial growth defects have been previously encountered when TEVp is expressed in yeast (22, 23), we observed that when expression of MBP-TEVp was induced with galactose in strain Sc_411, cell growth was arrested (Fig. 1*C*), potential implying that the expression of TEVp in mitochondria might bring about cleavage of unfolded proteins during import. Whatever the mechanism, these issues represent severe challenges to deployment of the TEVp-based *nif* polyprotein system in mitochondria.

### Knowledge-Based Engineering of a Minimal Sequence for Cleavage by the MPP.

**Fig. 2.**
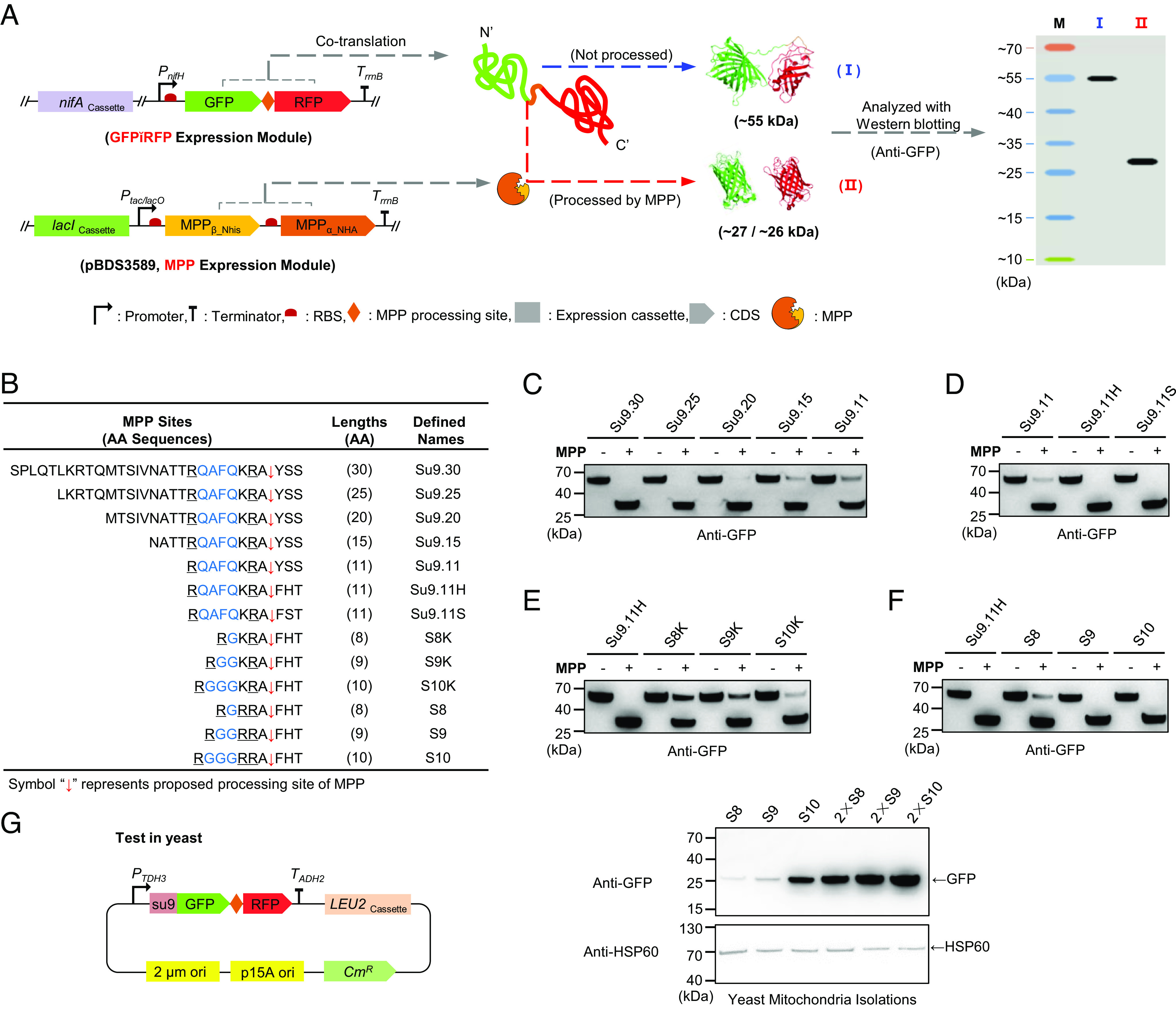
Screening for minimal MPP-processing sites. (*A*) Schematic diagram showing plasmid constructs and design procedure for testing the cleavage efficiency of reconstituted MPP in *E. coli.* GFP-RFP fusions linked by MPP cleavage sites were expressed from the σ^54^-dependent *P_nifH_* promoter from *K. oxytoca* and activated by NifA constitutively expressed from the *P_tet_* promoter. MPP (α subunit labeled with HA-tag and β subunit labeled with His-tag) were controlled by inducible P_tac/lacO_ promoter. The diamond symbol represents the MPP sites. (*B*) Sequences of the designed MPP sites. “AA” is short for amino acid. Arginine residues, which may be important for MPP recognition are underlined. Flexible linker regions are highlighted in blue color. The red symbol “↓” represents the proposed processing site of MPP. Panels (*C–F*) show immunoblotting assays to determine the cleavage efficiency of the various MPP sites listed in (*B*). The symbol “−”, indicates the absence of the MPP expression module; “+”, the presence of the MPP expression module induced with 100 μM IPTG. (*G*) Assessment of the cleavage efficiency of selected MPP sites in mitochondria of *S. cerevisiae*. The “2×” symbol indicates that tandem MPP sites were used. HSP60 was used as an internal reference for mitochondrial specificity.

Given the issues described above with TEVp-based cleavage, we considered the possibility of engineering a polyprotein system based upon an intrinsic mitochondrial protease. We decided to evaluate the use of MPP (25) for this purpose as it efficiently cleaves proteins after translocation into mitochondria, which could be desirable for processing polyproteins after import. Although we and others have encountered problems with MPP-like internal sequences within introduced “foreign” proteins such as NifD, this can be remedied by site-specific amino acid substitutions that retain activity (17, 19). As a first step toward engineering a polyprotein system based on MPP cleavage, we attempted to identify a minimal cleavage site that would be as short as possible, to avoid the negative effects of residual tails on the activity of processed proteins, but would nevertheless satisfy the requirement for efficient processing. To detect cleavage, we engineered a two-plasmid system in *E. coli*, in which a polyprotein with Green Fluorescent Protein fused to Red Fluorescent Protein (GFP-RFP) containing variant MPP sites was coexpressed with an expression module encoding the α and β subunits of *S. cerevisiae* MPP (19) (Fig. 2*A*). Cleavage was identified by western blotting using anti-GFP antibodies. If putative processing sites are recognized and cleaved by MPP, a ~27 kDa GFP peptide will be released, whereas in the absence of processing a ~55 kDa peptide representing the GFP-RFP fusion protein should be detected (Fig. 2*A*). We selected the Su9 presequence (residues 1 to 69 of ATP synthase subunit 9, from *Neurospora crassa*) for screening the efficiency of MPP processing, on the basis that this sequence targets proteins to mitochondria and is efficiently removed by MPPs (15, 26). We also observed previously that su9 is efficiently processed by reconstituted MPPs in *E. coli* (19).

It has been previously demonstrated that the su9 presequence is cleaved at two sites (27). To simplify our analysis, we chose residues 40 to 69 (designated here as Su9.30), which contains the second processing site, as the starting sequence for investigation (Fig. 2*B*). When GFP and RFP were linked by Su9.30, the polyprotein was completely processed by reconstituted Sc MPP in *E. coli* and a single ~27 kDa band was detected with the anti-GFP antibody (Fig. 2*C*). Shortening the sequence by five residues at the N terminus (Su9.25) gave similar results, but further removal of N-terminal residues (Su9.20, Su9.15, and Su9.11) decreases the processing efficiency in proportion to their length (Fig. 2*C*). We then focused on residues at the C terminus of su9.30, which contain the cleavage site itself, particularly the amino acid sequence KRAYSS from residues 25 to 30. Previous analysis has suggested that the most efficient combination of amino acid residues for processing by MPP at positions corresponding to residues 28 to 30 (known as the P1′, P2′, and P3′ positions respectively) is either FHT or FST (28, 29). We therefore replaced the C-terminal YSS sequence in Su9.11, with either of these sequences to form 11 residue peptides designated Su9.11H and Su9.11S respectively (Fig. 2*B*). Both of these substitutions enabled complete processing of the GFP-RFP polyprotein by MPP (Fig. 2*D*). We then turned our attention to sequences immediately upstream of the cleavage site. 80% of presequences contain an arginine residue either at position −2 (−2R) or −3 (−3R) (25) and in addition, a flexible region between this conserved arginine and a more distal arginine, located several residues upstream, is also important for processing (30). In an attempt to further shorten the scissile peptide for MPP cleavage, we substituted the QAFQ sequence of Su9.11H (located between the original positions 21 to 24 in Su9.30) with either one, two, or three glycine residues to generate peptides S8K, S9K, and S10K, respectively (Fig. 2*B*). Unfortunately, none of the three sequences was efficiently cleaved (Fig. 2*E*). However, considering that arginine at the −3 position is widely prevalent among presequences (25), we replaced the lysine at the −3 position with arginine to form peptides S8, S9 and S10 (Fig. 2*B*) and observed that both S9 and S10 could be completely processed in *E. coli* (Fig. 2*F*).

To determine if the artificial processing sites work in yeast mitochondria, GFP-RFP fusions with internal S8, S9, and S10 sites were constructed with the su9 leader sequence for targeting to mitochondria and were expressed under the control of the strong constitutive TDH3 promoter in *S. cerevisiae* (Fig. 2*G*). Previous experiments with TEVp cleavage in yeast (22) and with our TEV protease base polyprotein system (21) suggests that multiple copies of processing sites may improve cleavage efficiency. We therefore additionally included dual copies of the S8, S9, and S10 sequences in the GFP-RFP fusion proteins (Fig. 2*G*). When mitochondrial extracts from yeast strains expressing these constructs were assayed by immunoblotting with anti-GFP antibodies, only one band with similar migration to processed GFP was detectable (Fig. 2*G*), suggesting that unprocessed GFP-RFP polyprotein is susceptible to degradation in mitochondria. This implies that the level of processed GFP observed in the immunoblot is proportional to the processing efficiency. Since the dual copy of S10 gave the strongest GFP signal, suggesting the highest processing efficiency in yeast mitochondria, this double copy of the S10 peptide (RGGGRRAFHT) was selected for further polyprotein construction in yeast (Fig. 2*G*).

### Functional Reconstitution of Molybdenum Nitrogenase in *E. coli* with an MPP-Based Polyprotein System.

**Fig. 3.**
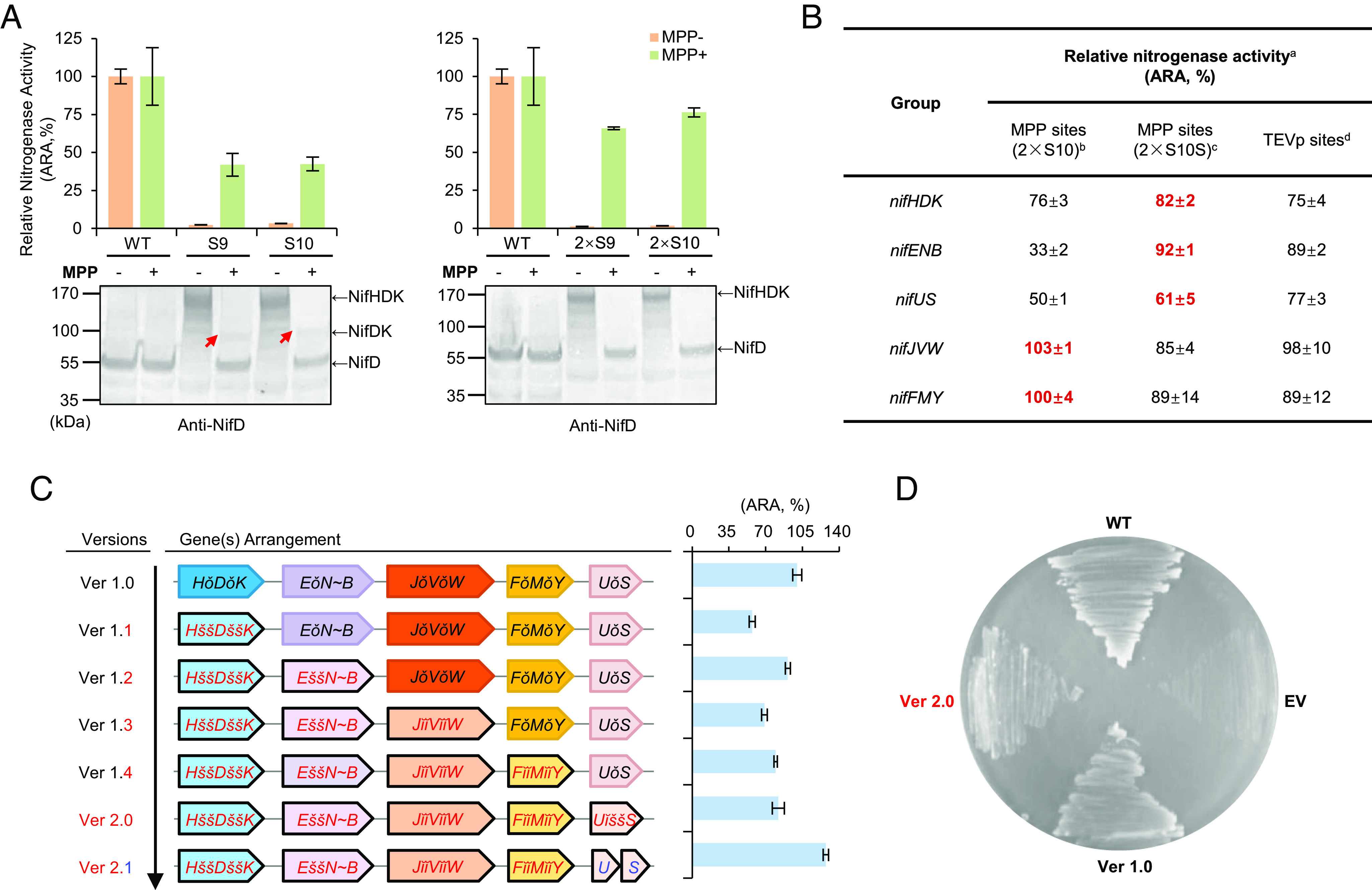
Assembly and characterization of the MPP-based Nif polyprotein system in *E. coli*. (*A*) Example of a dataset for *nifHDK* encoded polyproteins linked by different MPP cleavage sequences (listed in Fig. 2*B*). The acetylene reduction assay (ARA) was used to measure complementation by the *nifHDK* giant gene by the remaining *nif* genes in the operon-based system, either in the absence of MPP (orange bars, plasmid pKU7871) or presence of MPP (green bars, plasmid pG32) after induction with IPTG. WT, indicates complementation by the native *nifHDKTY* operon carrying the NifD-Y100Q substitution and this activity was assigned as 100% (24.2 ± 1.1 nmol C_2_H_4_/min/mg total protein). S9 and S10 indicate single copies of the S9 site (RGGRRAFHT) or the S10 site (RGGGRRAFHT) respectively. Tandem processing sites are indicated by 2 × S9 and 2 × S10, respectively. Red arrows indicate the unprocessed NifDK protein. (*B*) Summary data for the *nifHDK*, *nifENB*, *nifUS*, *nifJVW*, and *nifFMY* encoded polyproteins ^a^Acetylene reduction activities restored by the corresponding native *nif* genes or operons were assigned as 100%. ^b^2 × S10: indicates dual S10 sites (RGGGRRAFHT). ^c^2 × S10S: indicates dual S10S sites (RGGGRRAFST). ^d^Data from Yang et al. (21). (*C*) Schematic diagram showing the process of assembly in which TEVp-based giant genes where replaced by MPP-based giant genes. Each ensemble was assigned a version number (Ver 1.0 to Ver 1.4, Ver 2.0, and Ver 2.1, respectively) listed on the left and analyzed by acetylene reduction. Symbols “ǒ”, “ǐǐ”, and “šš” are used to represent twofold TEVp sites, MPP S10 sites and S10S sites, respectively. In each case, ARA activities are expressed as a percentage of the activity exhibited by the TEVp-based polyprotein system (Ver 1.0), which was assigned as 100%. To detect activities of strains carrying Ver 1.1 to 1.4, both MPP and TEVp were expressed concomitantly (cotransformed with pBDS3668 plasmid). Error bars in panels (*A–C*) indicate the SD observed from at least two biological replicates. (*D*) Diazotrophic growth promoted by TEVp-based and MPP-based polyprotein systems in *E. coli*. WT represents the reconstituted operon-based *nif* system. EV represents empty vector (pBDS1549), used as a negative control. Ver1.0 and Ver 2.0 represent the TEVp- and MPP-based polyprotein system as shown in (*C*), respectively.

To determine if it is feasible to engineer an MPP-based polyprotein system that functions to support biosynthesis and activity of nitrogenase, we reengineered our previous polyprotein system consisting of five giant *nif* genes (21), by sequential replacement of TEVp sites with MPP cleavage sites. We considered it appropriate to maintain the same gene order in each polyprotein as in the TEVp-based system (Fig. 1*A*), since this was previously optimized for Nif component stochiometry, nitrogenase activity, and diazotrophic growth in *E. coli* (21). We first examined the functionality of each individual reengineered polyprotein, both before and after cleavage with reconstituted yeast MPP, by measuring nitrogenase activities obtained from each giant gene when complemented with the remainder of the native *nif* genes in *E. coli.* Since native NifD is susceptible to internal cleavage by MPP (17, 19), we utilized the variant NifD-Y100Q protein, which retains maximum activity (*SI Appendix*, Fig. S4) and is resistant to MPP cleavage (17), for all relevant experiments in this study. To provide a comprehensive assessment of the MPP-based polyprotein strategy, four *nifHDK* giant genes with both single and dual copies of the S9 and S10 sequences (Fig. 2*B*) were constructed. When assayed for acetylene reduction, the giant gene *nifHǐǐDǐǐK* carrying twofold S10 MPP sites (indicated by *ǐǐ*) showed the highest activity (~76% compared to the native *nifHDK* operon) in line with efficient cleavage of the NifHǐǐDǐǐK polyprotein by MPP, as indicated by immunoblotting with anti-NifD antibody (Fig. 3*A*). In contrast, the NifHǐDǐK polyprotein with a single copy of the S9 or S10 site was less well cleaved, conversant with lower nitrogenase activities after cleavage with MPP (Fig. 3*A*). We therefore used dual S10 sequences to examine the functionality of the other giant *nif* genes.

Although the *nifJǐǐVǐǐW*, and *nifFǐǐMǐǐY* assemblies gave similar or slightly higher nitrogenase activities compared with the TEVp-based polyprotein version, lower activities were realized from the *nifEǐǐN~B* and *nifUǐǐS* giant genes containing dual S10 sites (Fig. 3*B*). Immunoblotting of extracts derived from the strain expressing the *nifEǐǐN~B* S10 assembly with anti-NifE antibody revealed only weak signals corresponding to the polyprotein or isolated NifE (*SI Appendix*, Fig. S5*A*), perhaps implying that cleavage of this construct is inefficient and the polyprotein is unstable. In contrast, the NifUǐǐS S10 polyprotein was apparently cleaved appropriately by MPP to release NifU (*SI Appendix*, Fig. S5*B*). In an attempt to improve processing of the NifEǐǐN~B polyprotein, we made a single amino acid substitution in the C terminus of the S10 sequence to replace the histidine residue with a serine, thus converting the C-terminal FHT residues to FST. Although this substitution did not apparently influence cleavage of a 11-residue peptide in our test system (compare sequences designated S9.11H and S9.11S in Fig. 2 *B* and *D*, respectively) a serine residue is prevalent at this position, and we considered that this might influence processing in the context of the flanking protein sequences as well as change the N-terminal tail present on proteins after cleavage. We therefore replaced the S10 sites in the NifENB polyprotein with the sequence designated as the S10S (RGGGRRAFST), to assemble the giant gene *nifEššN~B* (where šš indicates S10S).

Surprisingly, the S10S substitution substantially increased release of NifE from the NifE*šš*N~B polyprotein (*SI Appendix*, Fig. S4*A*) and also increased nitrogenase activity to ~92% of the control value, close to the activity of the TEVp version (Fig. 3*B*). Subsequently, we tested if the S10S sequence could improve the functionality of the other four polyproteins. Accordingly, the giant genes *nifHššDššK*, *nifUššS*, *nifJššVššW*, and *nifFššMššY* were assembled and assayed for activity after cleavage with MPP. A slight improvement for the *nifHDK* and *nifUS* groups was observed, and decreased activities for *nifJVW* and *nifFMY* were detected (Fig. 3*B*). However, in all cases, no obvious difference in protein levels were evident between the S10 and S10S versions (*SI Appendix*, Fig. S4).

To combine the MPP version of polyproteins into a functional Nif system, we sequentially replaced the TEVp-based giant genes with MPP-based versions, selecting the giant genes *nifHššDššK*, *nifEššN~B nifUššS*, *nifJǐǐVǐǐW*, and *nifFǐǐMǐǐY,* which exhibited the highest nitrogenase activity, for the final assembly. After five rounds of replacements, a completely MPP-based polyprotein system was constructed (designated version 2.0), retaining around 82% activity compared to the TEVp-based system (version 1.0) (Fig. 3*C*). As the MPP-based NifUS polyprotein only recovered up to 61% activity when expressed with the remaining native Nif components (Fig. 3*B*), this may represent the key limitation in assembling a highly active polyprotein–based nitrogenase system. We therefore constructed a version with *nifU* and *nifS* expressed as separate genes combined with the other four polyproteins (version 2.1). As anticipated, this version increased activity to ~127% compared with the TEVp version (Fig. 3*C*). As reported previously for the TEVp-based polyprotein system, the MPP-based version also supported diazotrophic growth of *E. coli*, confirming that this nitrogenase system is also fully functional (Fig. 3*D*).

### Stoichiometric Expression of Nif Component Proteins in Yeast Mitochondria Using MPP-Based Polyproteins.

**Fig. 4.**
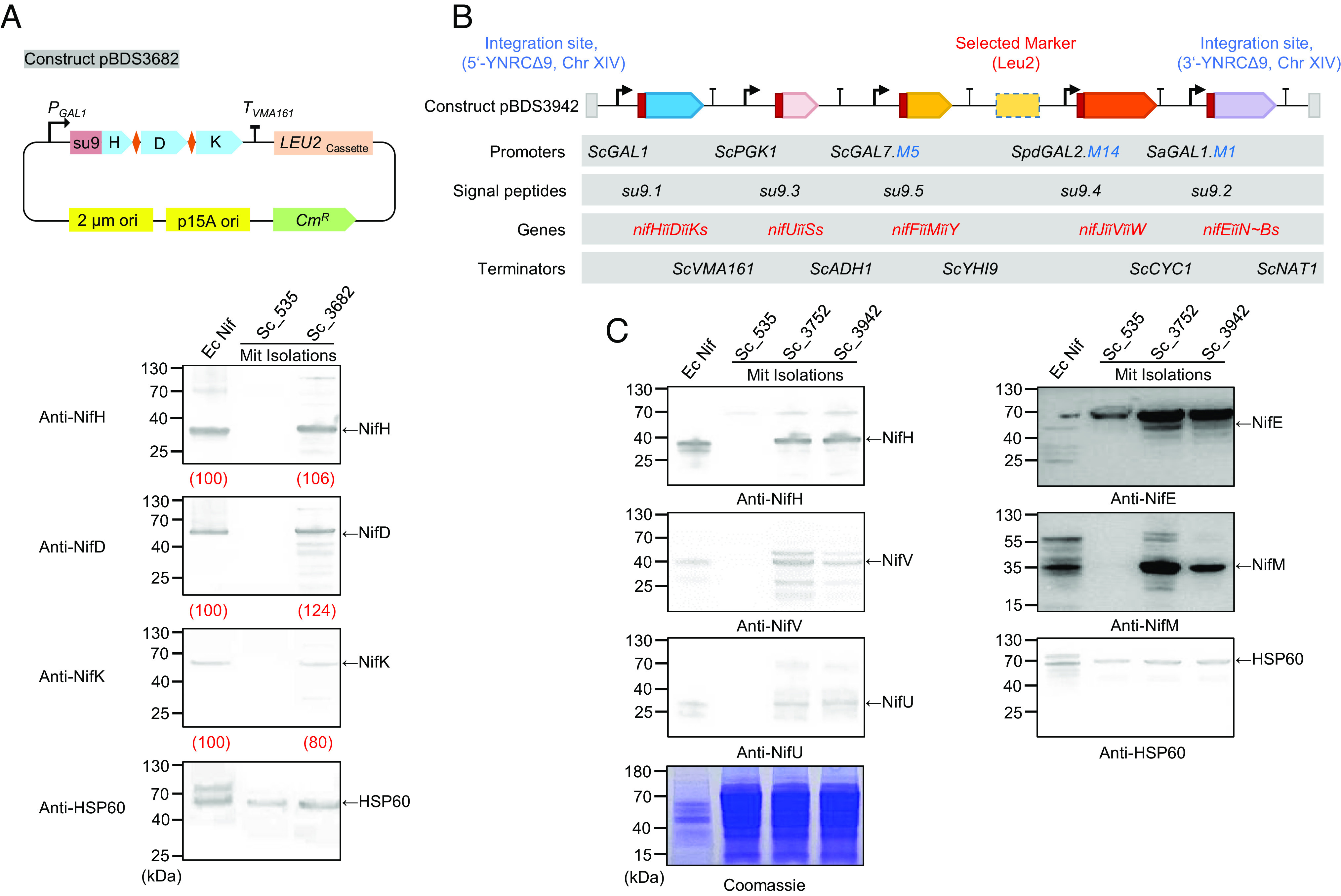
Assessment of MPP-based Nif polyprotein expression in yeast mitochondria. (*A*) Example of the NifHššDššK polyprotein expressed from the P*_ScGAL_*_1_ promoter and targeted to mitochondria with the Su9 leader sequence in the yeast strain Sc_3682. Ec Nif indicates protein samples prepared from *E. coli* cells carrying the reconstituted operon-based *nif* system; Sc_535 is a yeast strain transformed with the empty vector pBDS535, used as a negative control. “Mit Isolations” indicates protein samples prepared from mitochondrial extracts. Image J software was used for protein quantification, and relative expression levels are shown in red (in parentheses as a percentage below lanes) (*B*) Schematic diagram showing parts used to build constructs pBDS3752 and pBDS3942. The only difference between these two constructs is the presence of promoter variants in pBDS3942 highlighted in red. Giant genes are highlighted in blue and the symbols “ĭĭ” and “šš” are used to represent dual MPP S10 and S10S sites respectively. The selection marker and integration site are the same as described in Fig. 1*A*. (*C*) Immunoblotting of yeast strains carrying MPP-based Nif polyprotein systems. One representative protein from each polyprotein was selected for the immunoblot assay. HSP60 was used as an internal reference and a Coomassie blue–stained PAGE gel was used as control for equal loading of each sample.

Since functionality was retained when Nif polyproteins were processed by reconstituted MPP in *E. coli*, we next determined if they could be targeted to *S. cerevisiae* mitochondria and correctly processed in situ. The NifHDK group, representing the key structural components of molybdenum nitrogenase, was first selected as an example. The giant gene *nifHššDššK* in which *nif* genes were codon optimized (Dataset S1) was fused to the ScGAL1 promoter and the Su9 coding sequence for targeting to mitochondria (Fig. 4*A*). When assayed by immunoblotting, mitochondrial extracts from the engineered yeast strain Sc_3682, exhibited bands corresponding to NifH, NifD, and NifK, indicating that the NifHššDššK polyprotein is efficiently cleaved by native MPP after transport into mitochondria (Fig. 4*A*). To evaluate the stoichiometric ratio of the processed proteins, the loading volumes were adjusted to provide a similar band intensity for NifH in both the mitochondrial extract and the extract from *E. coli* expressing native Nif proteins. Quantification of the band intensities revealed that the stochiometry of processed NifH, NifD, and NifK in mitochondria was similar (Fig. 4*A*, values indicated in red). However, NifD was apparently more abundant than NifK in mitochondria compared with the stochiometric level of NifD and NifK expressed from the native *nifHDK* operon in *E. coli* (Fig. 4*A*). This bias potentially results from incomplete translation of the polyprotein caused by ribosome stalling (31).

To assemble the complete MPP-based *nif* polyprotein system in yeast, the five giant *nif* genes shown in Fig. 3*C* version 2.0 (from plasmid pNG653, Dataset S1) were coexpressed using the same promoters and terminators used for the TEVp-based system and were fused to variants of the Su9 signal peptide to target polyproteins to mitochondria (Fig. 4*B* and *SI Appendix*, Fig. S6*A*). The final construct (pBDS3752) was transformed into *S. cerevisiae* to generate stable strain Sc_3752 (*SI Appendix*, Fig. S6). Immunoblotting revealed that the stoichiometry of NifH, NifD, and NifK expressed in mitochondrial extracts from the complete Nif polyprotein system was again similar to the operon-based Nif system in *E. coli* and this was also observed for NifU and NifS (*SI Appendix*, Fig. S6*B*). However, the expression level of NifENB seemed to be higher than in *E. coli*. NifJV, NifM and NifY also were more highly expressed in the mitochondrial extracts (*SI Appendix*, Fig. S6*B*).

In order to mimic the balance of Nif component expression observed in *E. coli* we attempted to fine-tune expression of the NifENB, NifJVW, and NifFMY polyproteins in yeast using promoter variants. Three promoters were subjected to random mutagenesis by error-prone PCR and promoter strength was quantified using an RFP reporter assay (*SI Appendix*, Fig. S7). Three promoter variants derived from each group with broad ranging expression levels were then selected to drive expression of the *nifEššN~B*, *nifJǐǐVǐǐW,* and *nifFǐǐMǐǐY* giant genes and extracts from strains were analyzed for expression of representative Nif proteins in yeast mitochondria (*SI Appendix*, Fig. S7). Notably, promoter strength did not necessarily correlate with the level of protein expression. Nevertheless, reducing promoter strength to ~6% (for the Sa GAL1.M1) promoter and ~7% (for the Spd GAL2.M8 promoter) resulted in levels of NifE and NifV, respectively that matched the expression levels of the corresponding proteins in *E. coli* (*SI Appendix*, Fig. S7 *B* and *C*). For the *nifFMY* encoded polyprotein, the promoter variant of Sc GAL7 with 20% strength showed the lowest NifM output, which was still slightly higher than the protein level in *E. coli* (*SI Appendix*, Fig. S7*D*). As observed previously, with the TEVp-based polyprotein system (1), relatively high levels of NifF, NifM, and NifY expression did not have a significant impact on nitrogenase activity using the MPP-based polyprotein (Fig. 3*B* and *SI Appendix*, Fig. S5*E*). We therefore decided not to further optimize the expression level of the FMY group. By replacing the three promoters in construct pBDS3752 with the corresponding promoter variants to drive expression of the *nifEššN~B*, *nifJǐǐVǐǐW*, and *nifFǐǐMǐǐY* giant genes, we obtained construct pBDS3942 and its derivative yeast strain Sc_3942 (Fig. 4*B*). Immunoblotting of NifH, NifU, NifE, NifV, and NifM, representing each polyprotein group, demonstrated that strain Sc_3942 provided a more appropriate stoichiometric ratio related to the reconstituted operon-based Nif system from *E. coli* in comparison with the original strain Sc_3752 (Fig. 4*C*). However, acetylene reduction assays revealed no detectable nitrogenase activity in this strain irrespective of the presence of oxygen or when measured at different temperatures (*SI Appendix*, Fig. S8). This may reflect the relatively poor solubility of Nif component proteins when expressed in eukaryotes (11, 15) since only NifH and NifV showed detectable amounts of protein in the supernatant and most component proteins were apparently insoluble (*SI Appendix*, Fig. S9).

### Application of the MPP-Based Polyprotein Strategy to Reconstitute Other Heterologous Pathways in Mitochondria.

**Fig. 5.**
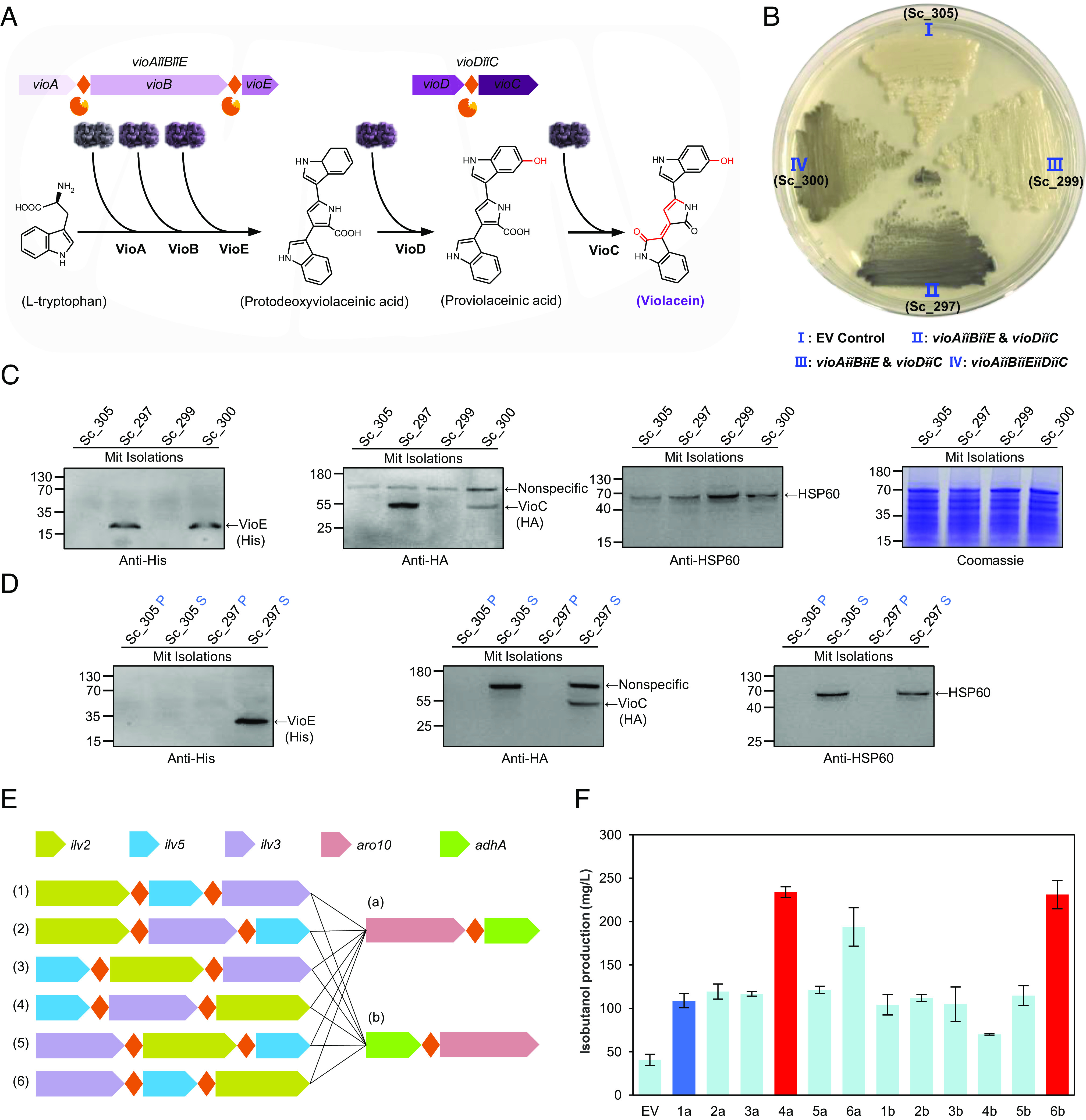
Engineering of the violacein and isobutanol biosynthesis pathways in mitochondria using the MPP-based polyprotein strategy in *S. cerevisiae*. (*A*) Schematic diagram showing the violacein biosynthesis pathway and gene arrangement in the polyproteins. (*B*) Petri dish experiment displaying the violet pigment biosynthesized by the MPP-based Vio polyprotein in yeast mitochondria. I: yeast strain transformed with the empty vector (pNG305). II: yeast strain transformed with the MPP-based *vioAǐǐBǐǐE and vioDǐǐC* polyprotein system carrying dual S10 sites (pNG297), in which five violacein biosynthesis genes were assembled as two polyproteins encoded by giant genes. III: yeast strain transformed with MPP-based *vioAǐǐBǐǐE and vioDǐǐC* polyprotein system carrying noncleavable S10 site variants (pNG299). The symbol “ǐǐ” represents the dual S10 site variant, in which the arginine residues at positions −2 and −3 were replaced by two alanine residues (RGGGAAAFSTRGGGAAAFST), to provide a negative control; IV: yeast strain transformed with the MPP-based *vioAǐǐBǐǐEǐǐDǐǐC* polyprotein system with tandem S10 sites (pNG300), in which five violacein biosynthesis genes were assembled as a giant gene encoding a single polyprotein. Immunoblotting (*C*) and solubility (*D*) analysis of Vio proteins extracted from the corresponding yeast strains indicated in (*B*). VioE and VioC proteins were labeled with hexahistidine and HA tags, respectively. “P” and “S” in blue color represent the insoluble and soluble fraction of proteins isolated from mitochondria respectively. “Nonspecific” indicates a protein present in yeast extracts that cross-reacts with the anti-HA antibody. “Mit Isolations” indicates protein samples prepared from mitochondrial extracts. HSP60 was used as internal reference and a Coomassie blue–stained PAGE gel was used as control for equal loading of each sample. (*E*) Schematic diagram showing the pathway genes and the combinatorial strategy employed to optimize isobutanol biosynthesis in yeast mitochondria using polyproteins. (*F*) Isobutanol production from MPP-based polyprotein combinations shown in (*E*). EV, indicates empty vector (plasmid pNG255), to assess the native level of isobutanol synthesized in yeast. Polyprotein combinations with the highest isobutanol production level are shown in red. Detailed information for each plasmid is provided in Dataset S1.

Having successfully engineered a strain expressing stochiometric levels of Nif component proteins in yeast mitochondria, we investigated whether MPP-based polyproteins can provide a universal strategy for coexpression of functional components in this organelle. Well-established pathways for violacein and isobutanol biosynthesis were selected for this engineering. Violacein is an indolocarbazole pigment synthesized in bacteria by five enzymes encoded by the *vioABCDE* operon using tryptophan as a substrate (32). Since pathway catalysis proceeds in the order VioA, VioB, VioE, followed by VioD, and VioC (Fig. 5*A*), we expressed the enzymes in two polyproteins encoded by the giant genes *vioAǐǐBǐǐE* and *vioDǐǐC*, each driven by the TDH3 promoter and targeted to mitochondria with Su9 leader sequences (*SI Appendix*, Fig. S2*B*). As a control for these experiments, we also expressed VioABE and VioDC polyproteins linked by noncleavable versions of the S10 MPP processing site, in which the arginine residues at position −2 and −3 were replaced by alanine residues. When transformed into yeast, the dark violet-colored violacein pigment was synthesized in yeast carrying S10-linked polyproteins, whereas no pigment was synthesized from polyproteins linked by the noncleavable S10 peptide (Fig. 5*B*). We also assembled a giant gene, *vioAǐǐBǐǐEǐǐDǐǐC*, to express a longer polyprotein encompassing the complete violacein biosynthesis pathway and also observed synthesis of the violet-colored pigment, albeit lighter in color compared with yeast carrying the two separate giant genes (Fig. 5*B*). Immunoblotting assays demonstrated that Vio polyproteins linked with S10 sites were correctly processed, in contrast to polyproteins fused with noncleavable S10 peptides, which were undetectable (Fig. 5*C*). In agreement with the degree of pigmentation detected in vivo, we observed that the level of VioC processed from the longer VioAǐǐBǐǐEǐǐDǐǐC polyprotein was less than that from the shorter VioDC polyprotein (Fig. 5*C*). Differing from the Nif proteins, Vio proteins released from polyproteins expressed in mitochondria were soluble (Fig. 5*D*).

In yeast, isobutanol can be synthesized from pyruvate by three genes *ILV2*, *ILV3*, and *ILV5*, encoding proteins located in mitochondria and two genes *ARO10* and *ADHA*, encoding proteins located in the cytosol (33). A previous study revealed that compartmentalization of all proteins in this pathway in mitochondria remarkably increased isobutanol production (34). To examine if this could also be achieved by expressing the entire pathway as polyproteins targeted to mitochondria, we followed a similar strategy used for reconstitution of the violacein biosynthetic pathway by engineering constructs expressing *ILV2*, *ILV3*, and *ILV5* on one polyprotein, with *ARO10* and *ADHA* expressed as a separate group (*SI Appendix*, Fig. S2*B*). To optimize expression levels and take into account tailing tolerance (since processing of the S10 sequence by MPP results in the addition of a seven residue C-terminal tail to proteins located upstream of the cleavage site), we permuted the gene order in constructs to obtain a library of polyproteins with multiple rearrangements of the coding sequences (Fig. 5*E*). Although most permutations resulted in isobutanol synthesis, optimal levels were produced by construct 4a (*ILV5ǐǐ3ǐǐ2* combined with *ARO10ǐǐADHA*) or construct 6b (*ILV3ǐǐ5ǐǐ2* combined with *ADHAǐǐARO10*), which yielded around 230 mg/L isobutanol after 48-h fermentation (Fig. 5*F*).

## Discussion

The “fusion and cut” feature of the polyprotein strategy enables coexpression of protein components in eukaryotes at stoichiometric levels, thus enabling the engineering of protein complexes and intricate biochemical pathways requiring balanced gene expression. This strategy also dramatically reduces the number of expression parts for synthetic biology, thus decreasing the overall DNA cargo load and the requirement to diversify promoters and terminators in order to avoid homologous recombination. Although the complexity of eukaryotic gene regulation and the consequent DNA burden can be reduced through the deployment of minimal orthologous synthetic promoters and terminators (35), there is a lack of well-characterized expression parts in higher eukaryotes, especially plants (36). The polyprotein strategy therefore has significant advantages for engineering complex biological pathways in eukaryotes, since it not only provides balanced protein expression, but it also decreases the combinatorial complexity when selecting suitable expression parts for engineering multiple protein-coding sequences. Although coexpression of multiple Nif components, has already been achieved in both yeast and plants (10–12, 15, 16, 37–40) polyprotein systems provide unique opportunities to achieve coordinated and stoichiometric expression of nif genes in eukaryotic hosts as exemplified here.

In our previous study, the TEV protease–based cleavage strategy enabled successful reassembly of the *K. oxytoca nif* genes to encode polyproteins that supported the diazotrophic growth of *E. coli* after processing by the protease (21). However, this processing strategy does not appear to be suitable for polyproteins targeted to eukaryotic mitochondria. Although no previous issues have been reported with TEVp expression in yeast (22, 23, 41) and TEVp targeted to mitochondria has been utilized to process fumarase (42), we observe growth arrest when TEV is co-expressed and targeted to mitochondria with our polyproteins. Since Nif polypeptides are undetectable under these conditions, cleavage and release of misfolded proteins may result in proteotoxic stress (43). The endogenous MPP-based polyprotein strategy clearly bypasses the issues observed with the TEV-based system, potentially because each Nif component is cleaved and folded discretely as the polyprotein passes through the inner mitochondrial membrane. Sequential processing by MPP and separate folding of each protein component immediately after transport may have substantial advantages over the transport of full-length polyproteins into the mitochondrial matrix, which may fold incorrectly prior to subsequent cleavage by TEVp. Accordingly, induction of Nif polyprotein expression by galactose in the MPP-based polyprotein yeast strains Sc_3752 and Sc_3942 had minimal impacts on growth compared with induced expression of the TEVp-based system (*SI Appendix*, Fig. S10). Moreover, polyprotein cleavage by MPP after transport to mitochondria already occurs in nature. The enzymes N-acetylglutamate kinase and N-acetyl-γ-glutamyl-phosphate reductase are expressed as a polyprotein precursor, which is imported into mitochondria and subsequently processed by MPP to release two functional components in fungi (44). In addition, in rice the ribosomal protein S14 forms a polyprotein with succinate dehydrogenase B that is processed by MPP when targeted to mitochondria (45). Therefore the synthetic polyprotein approach should be applicable not only to yeast, but also to plants since the recognition and processing pattern of the MPPs from yeast and plants are very similar (46). However larger engineered polyproteins may present issues with components assigned to the C terminus of the polyprotein, as we detected decreased protein levels with components located at this position (Figs. 4*A* and 5*C*). We suggest this phenomenon may result from incomplete translation of the polyprotein caused by ribosome stalling (31). This situation should be taken into consideration when engineering multiple gene systems using polyprotein designs. Hence protein components with lower expression requirements could be assigned to the C terminus of the polyprotein.

In the current case, we have characterized a minimal cleavage sequence for processing by MPP to develop a polyprotein strategy for targeting to mitochondria that can be applied to the complex Nif system and mimics the protein stoichiometry observed with the analogous system expressed in *E. coli*, which is fully functional. However, we acknowledge that it is difficult to precisely measure protein stoichiometry after import into mitochondria given potential stability and solubility issues. No nitrogenase activity was detected in yeast, which is not unexpected considering the low level of protein solubility and the probable requirement for additional components to support nitrogen fixation in mitochondria. The problem of Nif component solubility in this organelle has been encountered previously both in yeast and plants (11, 12, 15, 39, 40). Since almost all Nif proteins from *K. oxytoca* are insoluble in mitochondria, physiological conditions within the mitochondrial matrix may be a key factor, including pH, metabolite concentration, or incompatibility of chaperones with Nif proteins. Exploiting biodiversity has provided a useful solution to this bottleneck, since for example, screening Nif component proteins from diverse diazotrophs, has enabled identification of *Hydrogenobacter thermophilus* (11) and NifB proteins from thermophilic archaea (15, 39, 40) with higher solubility in both yeast and plant mitochondria. After purification, these proteins function together with heterologous Nif components to fulfill their respective functions in vitro. *H. thermophilus* NifH purified from mitochondria retains the three important functions of nitrogenase Fe protein, supporting P cluster maturation, FeMo-co assembly and ATP-dependent electron transfer to Mo-Fe protein (11, 12). NifB plays a crucial role in nitrogenase active site cofactor biosynthesis, but NifB proteins from mesophilic diazotrophs are notoriously insoluble when expressed in eukaryotic mitochondria (15, 37). Nevertheless, two NifB proteins from the archaea *Methanocaldococcus infernus* and *Methanothermobacter thermautotrophicus* have been expressed in mitochondria in a soluble form when coexpressed with *A. vinelandii* accessory components and support FeMo-co biosynthesis in vitro (15, 37, 39, 40). However, the compatibility of heterologous Nif components derived from diverse diazotrophs may decrease efficiency when attempting to reconstruct a functional *nif* system. This is particularly pertinent when using thermophilic enzymes operating at suboptimal temperatures for catalysis (11). Alternative strategies to the biodiversity approach could potentially be deployed to solve solubility issues, including the deployment of additional chaperones and the use of machine learning methods for protein design, for example, the introduction of rationally engineered peptide tags to improve solubility (47).

Although the minimal *K. oxytoca* gene set comprising 13 *nif* genes, clearly enables diazotrophic growth of *E. coli*, some of the functions required to support nitrogen fixation are likely to be provided by housekeeping components encoded by the host genome, which are not present in plant organelles. One aspect that may affect the function of classical MoFe nitrogenase in mitochondria is the availability of the trace element molybdenum, which may be lacking in organelles since no molybdenum containing enzymes have been identified either in plant mitochondria or chloroplasts (48). Heterologous expression of a suitable molybdenum transporter targeted to mitochondria may be required to resolve this issue. In addition, the NifQ protein, which is involved in Mo mobilization and carries a Mo Fe-S cluster to donate Mo to the NifEN scaffold for FeMoco biosynthesis may also be required (49). Alternatively, this issue could be circumvented by engineering the iron-only nitrogenase, which is simpler and requires fewer genes than the Mo or V nitrogenase systems. Targeting of the Anf structural subunits to yeast mitochondria revealed that AnfH has functional Fe protein activity and AnfDK could be activated for substrate reduction by addition of FeMo-co in vitro. This implies that yeast mitochondrial apo-AnfDK is replete with P-clusters, even though coexpression of NifU and NifS was not required for apo-AnfDK activation (50). However, our previous study indicated that the Anf system may not be suitable for the polyprotein approach since AnfH, AnfG, and AnfK are intolerant to the presence of the C-terminal tail resulting from protease TEVp cleavage (21). In addition to the molybdenum issue, other accessory proteins may also be required to ensure efficient synthesis of nitrogenase active site cofactors. Although the *fdxN* gene, which is commonly associated with *nifB* in several diazotrophs, is not present in the *K. oxytoca* nif gene cluster, deletion of this gene in *A. vinelandii* impairs diazotrophic growth and FdxN is important for the activity of *A. vinelandii* NifB in yeast mitochondria (15). The precise role of FdxN in NifB-co biosynthesis is currently unknown, but it is possible that an analogous function is provided by gene products encoded elsewhere in the *K. oxytoca* and *E. coli* genomes.

There is growing interest in the subcellular compartmentalization of metabolic pathways within organelles, which can offer unique physiological environments separate from the cytosol. Organelle compartmentalization can provide increased concentrations of substrates and enzymes to enhance pathway efficiency and avoid metabolic crosstalk. This strategy has been applied not only to chloroplasts and mitochondria, but also to other organelles including peroxisomes, vacuoles, the endoplasmic reticulum (ER) and the Golgi (51). We have demonstrated that our targeted polyprotein approach, based on processing by the mitochondrial matrix peptidase can simplify metabolic pathway reconstruction in yeast by engineering giant genes that functionally confine biosynthesis of the bacterial pigment violacein and the biofuel isobutanol to mitochondria. This strategy could also be generalized to other subcellular components, such as the chloroplast, ER and vacuoles, since all these organelles utilize a specific processing peptidase for signal peptide cleavage that could be deployed for polyprotein processing (52), particularly in the case of complex pathways that require many components and balanced expression for function.

## Materials and Methods

### Strains and Media.

*E. coli* strains JM109 and DH5α were used for routine cloning and plasmid propagation. JM109 was used to screen functional MPP-processing sites and measure nitrogenase activity by the acetylene reduction assay. *E. coli* strain NCM3722 was used as host for diazotrophic growth experiments. Media for *E. coli* growth, nitrogen fixation assays, or diazotrophic growth were used as described previously (21). The *S. cerevisiae* strain W303-1a (MATα ade2-1 leu2-3,112 trp1-1 his3-11,15 ura3-1) was used as the host to express polyprotein-based *nif* systems. The *S. cerevisiae* S288C URA3 minus strain (*KanMX::URA3*) was used as the host to express the polyprotein-based violacein biosynthesis and isobutanol biosynthesis pathways. Media for *S. cerevisiae* growth and induction of Nif protein expression were used as previously described (19).

### Plasmids Construction.

Plasmids used in this study and all gene sequences used for expression of the violacein biosynthesis pathway, isobutanol biosynthetic pathway, and the *nif* system in yeast are listed in the Dataset S1. All plasmids were verified by sequencing before use for further experiments. The MPP expression modules used for MPP site screening and reconstruction of the MPP-based *nif* system were constructed in a previous study (19). Genes encoding violacein biosynthesis were amplified from a plasmid kindly provided by Bingzhi Li (Tianjin University). The genes *ILV2*, *ILV3*, *ILV5,* and *ARO10* encoding for the isobutanol biosynthetic pathway were directly amplified from the *S. cerevisiae* genome and gene *ADHA* from *Lactococcus lactis* was chemically synthesized. Genes encoding each Nif component were chemically synthesized by the GenScript Company according to the codon bias of S. *cerevisiae* nuclear genes. All giant genes and corresponding expression cassettes were assembled via Golden Gate assembly, with the coding sequences for MPP recognition sites added by PCR. Schematic diagrams of the detailed assembly process is provided in *SI Appendix*, Figs. S1 and S2. Sequences of the MPP-based polyprotein *nif* system with optimal nitrogenase activity in *E. coli* (ver2.0 in Fig. 3*C*, construct pNG653) and the MPP-based polyprotein *nif* system with optimal stoichiometric ratio (construct pBDS3942 in Fig. 4*B*) are shown in Dataset S2.

### MPP Site Screening in *E. coli*.

Strains carrying corresponding plasmids were incubated in LB medium in 24-well plates, at 30 °C, for 24 h, and 100 μM of IPTG was used to induce the expression of the MPP protein. Then 800 μL of bacteria cells were collected and washed once with PBS buffer. The subsequent pellets were resuspended in 240 μL of PBS buffer and 60 μL of 5× SDS loading buffer (Sangon; C508320) was added. Samples were boiled for 30 min and centrifuged for 2 min at 12,000 rpm. The supernatants were used for further immunoblotting assays.

### Acetylene Reduction and Diazotrophic Growth Assays of Reconstituted *E. coli* Strains.

Methods for measuring acetylene reduction (ARA) and assessing diazotrophic growth were deployed as described previously (19). Isopropyl-β-d-thiogalactoside (IPTG) was used to induce the expression of MPP for ARA. Immediately after the acetylene reduction assay, 4 mL (two parallel samples, OD_600_ = ∼1.0) of *E. coli* cells carrying the appropriate plasmids were collected and suspended in 300 μL of PBS buffer prior to the addition of 75 μL of 5× SDS loading buffer. Samples were boiled for 30 min and centrifuged for 2 min at 12,000 rpm. To determine the capacity for diazotrophic growth, MPP was expressed from a constitutively activated promoter (Dataset S1).

### Yeast Transformation and Mitochondria Extraction.

Yeast transformations were carried out according to the lithium acetate method (53), and transformants were selected on solid synthetic dropout medium. For extraction from mitochondria, *S. cerevisiae* carrying the corresponding constructs were grown in flasks in YPDG medium (containing 20 g/L peptone, 10 g/L yeast extract, 10 g/L glucose, 10 g/L galactose, and 100 mg/L adenine) at 30 °C and 200 rpm for 36 h. Yeast cells were collected and mitochondrial enriched protein extracts were prepared as described previously (19, 54) and purity was verified using actin as cytosolic marker (*SI Appendix*, Fig. S11). Total proteins were extracted by directly resuspending the crude mitochondria in PBS to a final concentration of 40 mg/mL. For soluble protein extraction, the crude mitochondria were resuspended in nondenaturing lysis buffer (containing 50 mM Tris-HCl, pH8.0, 150 mM NaCl, and 1% Triton X-100) to a final concentration of 40 mg/mL. The mixtures were then vortexed for 15 s at maximum speed, and incubated on ice for 10 min. Subsequently, the mixtures were vortexed for 60 s and centrifuged at 20,000 g for 10 min at 4 °C. The supernatants were collected representing the soluble fraction, and the pellets were resuspended in PBS buffer to represent insoluble proteins. After addition of the appropriate amount of 5× SDS loading buffer, the samples were boiled for 30 min and centrifuged for 2 min at 12,000 rpm.

### Western Blotting.

Immunoblotting was performed according to the method described previously (19). Briefly, samples were loaded on 10% SDS-polyacrylamide gels (Thermo Fisher Scientific; NP0301, NP0302) with 5 μL of PageRuler Prestained Protein Ladder (Thermo Fisher Scientific; 26616) as a marker. Proteins on the gels were subsequently transferred to PVDF membranes (Thermo Fisher Scientific; IB24002) using an iBolt 2 (Thermo Fisher Scientific). The membranes were blocked with 5% skim milk (BD Difco 232100; BD Biosciences) in PBS buffer and then incubated with primary antibodies for 4 to 8 h according to the sensitivity of the antibody for each Nif protein and labeled-tag. The secondary antibody goat anti-rabbit IgG-HRP (ZSGB Biotech; ZB-2301) or goat anti-mouse IgG-HRP (ZSGB Biotech; ZB-2305) was used at 1:3,000 dilution and incubated for 2 h before development. The primary antibodies used for immunoblotting and quantification in this study were as described previously (21), with the exception of different antibodies against GFP (ZSGB Biotech, TA-06), HSP60 (Proteintech, 66041-1-Ig) and Actin (HUABIO, HA601082).

### Violacein and Isobutanol Biosynthesis Assays.

To assess violacein biosynthesis, yeast carrying the corresponding plasmids were picked and streaked onto Yeast Peptone Dextrose (YPD) solid plates. Subsequently, the plates were incubated at 30°C for 3 d. To measure isobutanol biosynthesis, yeast carrying the corresponding plasmids were grown overnight in synthetic uracil minus medium with 2% glucose at 30 °C for 24 h. Five milliliter of each overnight culture was then centrifuged at 1,500 g for 3 min, the supernatant was discarded and the cells were resuspended in 5 mL of synthetic uracil minus medium with 10% glucose in sterile 50-mL conical tubes. Cells were grown in this medium for 24 h at 30 °C with 220 rpm agitation, after which they were centrifuged at 1,500 g for 3 min. The supernatant was filtered through a 0.2-µm filter membrane and used for isobutanol quantification. Isobutanol was quantified using a gas chromatograph (Shimadzu GC-2014C) equipped with a flame ionization detector and a FFAP column (30 m × 0.25 mm × 0.25 µm), with nitrogen as the carrier gas. The injector and detector were maintained at 250 and 280 °C, respectively. The column temperature was initially maintained at 70 °C for 1 min. It was then increased to 200 °C at a rate of 15 °C/min, and then maintained at this temperature for 2 min.

## Supplementary Material

Appendix 01 (PDF)

Dataset S01 (XLS)

Dataset S02 (PDF)

## Data Availability

All data, DNA sequences, and strain information are available in the main text, *SI Appendix*, and Datasets S1 and S2. Plasmids have been deposited at Addgene (https://www.addgene.org) (ID: 204447–204452) (55). All other data are included in the manuscript and/or supporting information.
