# Supplementary material for: Organelle-dependent polyprotein designs enable stoichiometric expression of nitrogen fixation components targeted to mitochondria
Source: Proc Natl Acad Sci U S A. 2023 Aug 16;120(34):e2305142120. doi: 10.1073/pnas.2305142120 (PMC10450427; doi:10.1073/pnas.2305142120)
Supplement: Supplementary file 1 — Appendix 01 (PDF) [file pnas.2305142120.sapp.pdf]

PNAS

Supporting Information for

Organelle-dependent polyprotein designs enable stoichiometric expression of nitrogen fixation components targeted to mitochondria

Jianguo Yang[a,1,2], Nan Xiang[a.1], Yiheng Liu[a], Chenyue Guo[a], Chenyu Li[a], Hui Li[a], Shuyi Cai[a], Ray Dixon[b,2], Yi-Ping Wang[a,2]

[a] State Key Laboratory of Protein and Plant Gene Research, School of Advanced Agricultural Sciences and School of Life Sciences, Peking University, Beijing 100871, China;
[b] Department of Molecular Microbiology, John Innes Centre, NR4 7UH Norwich, United Kingdom

[1] These two authors contributed equally to this work
[2] To whom correspondence may be addressed. Email: yangjg@pku.edu.cn, ray.dixon@jic.ac.uk, or wangyp@pku.edu.cn

This PDF file includes:

Supporting text
Figures S1 to S11

Other supporting materials for this manuscript include the following:

Datasets S01 to S02

Supporting Information Text

Yeast promoter random mutation and promoter strength quantification

Promoter sequences carrying random mutations were obtained by using the error prone PCR kit (Mei5 Biotechnology, MF298-01) with the corresponding original promoter sequence as template. Error prone PCR products were then assembled with the RFP reporter gene and the Sc ADH1 terminator on pBDS1549 vector by golden gate assembly. The subsequent mixture was directly transformed into W303-1a and incubated at 30℃ for 48 hrs. Single colonies were picked and inoculated in modified Leu DO medium (containing 13.4 g/L yeast nitrogen base [BD Biosciences; 291920], 0.69 g/L dropout mixture [-Leu Do supplement; BD Biosciences, 630414], 5 g/L glucose and 5 g/L galactose) in 96-well plates, at 30℃, for 36 hrs in a microplate reader (BIOTEK, SynergyH1). Real-time OD_{600} and fluorescence intensity of RFP were detected by the microplate reader. Strains with reduced fluorescence intensity (e.g., ~40%, 20%, or 6%) compared to strains carrying the original promoter were selected and used as template to amplify the mutated promoter.

Acetylene Reduction Assay for *S. cerevisiae* strains.

Yeast strains carrying corresponding constructs were incubated aerobically in YPD medium (containing 20 g/L peptone, 10 g/L yeast extract, 20 g/L glucose, and 100 mg/L adenine) for 24 hrs. The cell cultures were then centrifuged for 5 min at 1500 g, washed once with YPG medium (with glucose replaced by galactose in YPD medium) and then resuspended in YPG medium supplemented with 26 mg/L of $CaCl_2 \cdot 2H_2O$, 30 mg/L of $MgSO_4$, 0.3 mg/L of $MnSO_4$, 36 mg/L of ferric citrate, and 300 μM Na_2MoO_4 (tenfold higher than used for the nitrogenase activity assay in *E. coli*) to a final OD_{600} of 0.8. Subsequently, 10 mL of the cell culture was transferred into a 100 mL anaerobic bottle which was repeatedly evacuated and flushed with argon. 10 mL of C_2H_2 was injected as substrate for the acetylene reduction assay. For aerobic assays an additional 20 mL of oxygen was injected. After incubation at either 20°C or 30°C for 48 hrs, the gas phase was analyzed with a Shimadzu GC-2014 gas chromatograph.

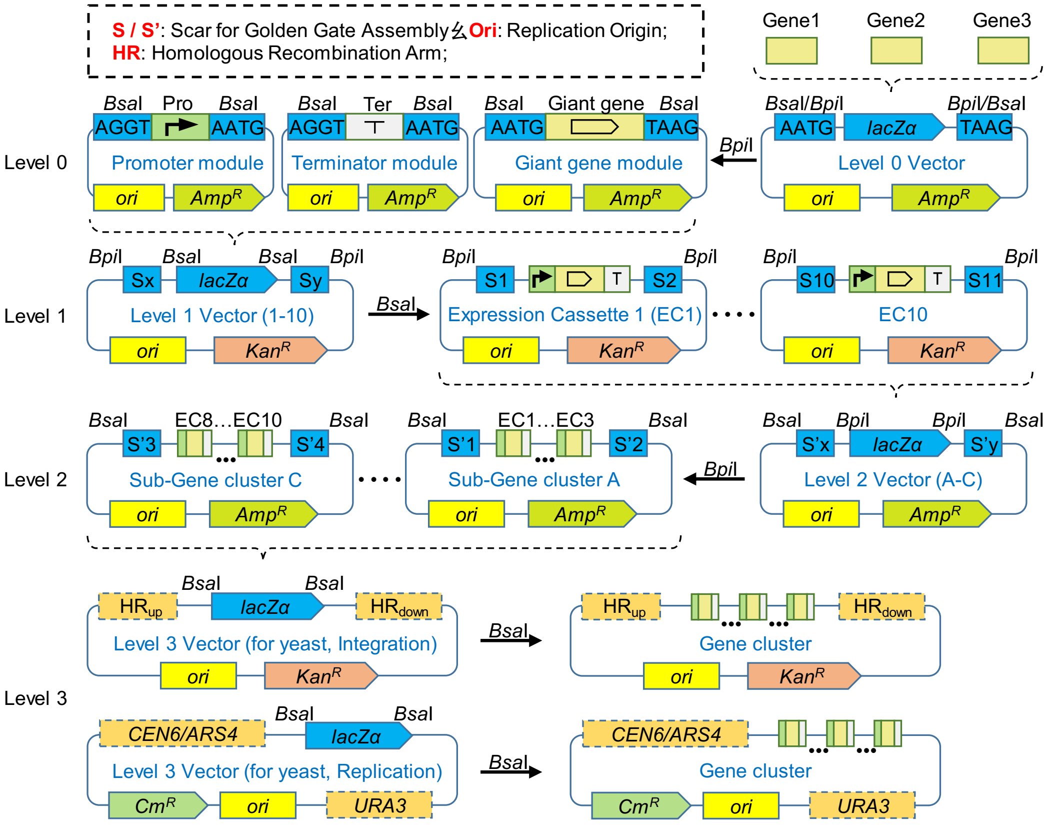

Fig. S1. Vectors for hierarchical assembly of multiple parts. The type IIS restriction enzymes *Bsa*I and *Bpi*I were used for hierarchical Golden Gate assembly. *Bsa*I and *Bpi*I sites located inside the sequences were removed either with point mutations method or overlap PCR. Identical *Bsa*I scars were assigned for all level 0 promoter modules (AGGT/AATG), terminator modules (TAAG/GCTT), and the giant gene modules (ORF module, AATG/TAAG). Ten Level 1 vectors (1-10) were constructed for assembling the promoter module, ORF module and terminator module into the general expression cassettes (EC). Three Level 2 vectors A-C were constructed for assembling EC1 to EC10 into sub-gene clusters. Vector A, B, and C was used for assembling EC1-3; EC4-7, and EC8-10 respectively. Ten plasmids carrying gap1-10 sequences were also constructed with identical *Bpi*I scars to the corresponding Level 1 vectors 1-10. Three plasmids carrying gapA-C sequences were also constructed with identical *Bsa*I scars to the corresponding Level 2 vectors A-C. These gap sequences were used when no corresponding expression cassette or sub-gene cluster existed. Two Level 3 vectors were constructed. One of these, carrying homologous recombination arms was used for Nif polyprotein system engineering, and the other one carrying the CEN6/ARS4 replication origin and the *URA3* expression cassette was used for violacein biosynthetic pathway engineering. The detailed information for each plasmid is provided in Dataset_S01.

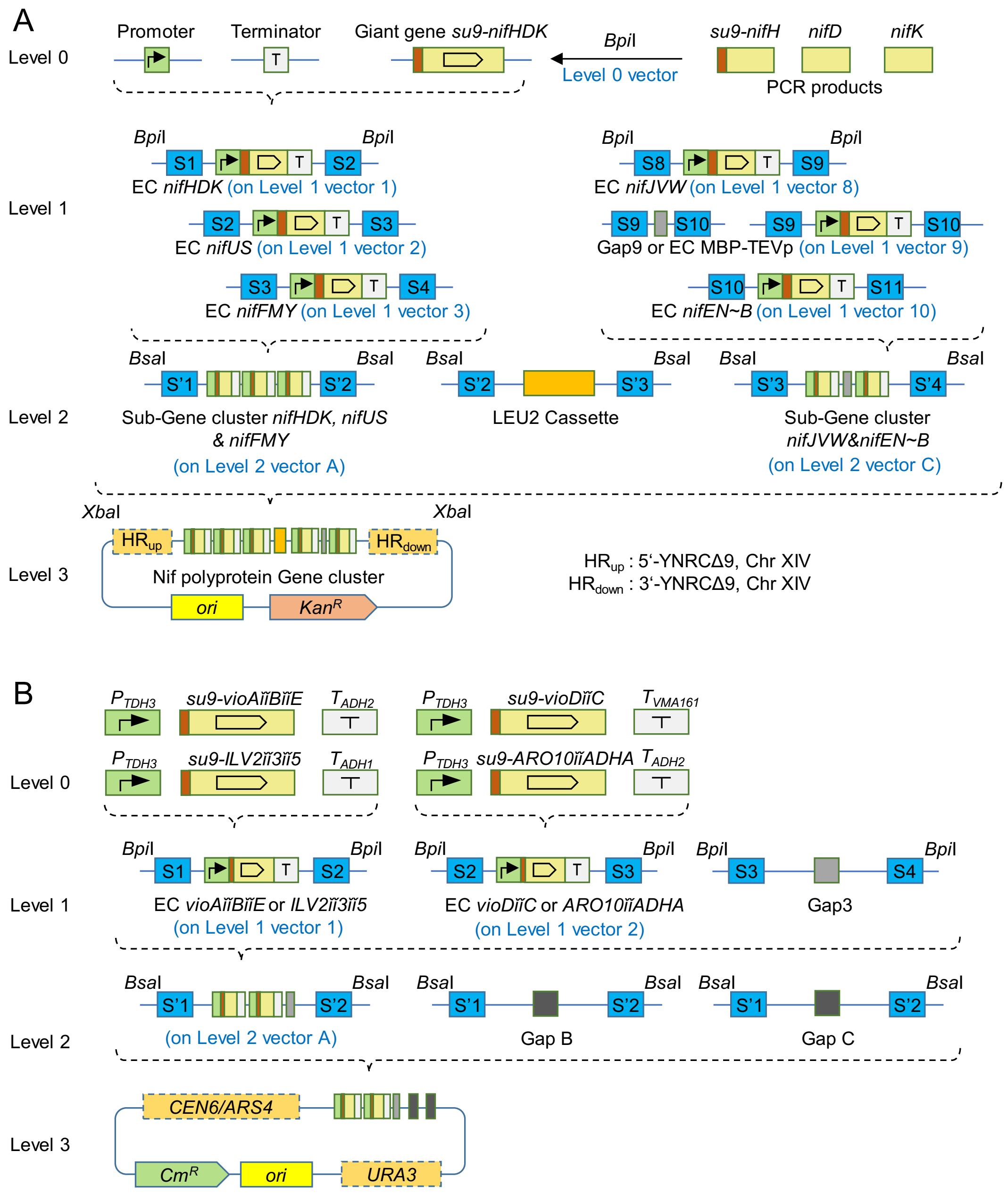

Fig. S2. Schematic diagram for plasmid assembly in this study. (A) Schematic diagram for assembly of the Nif polyprotein system. The su9 sequence was added to the first gene by overlap PCR. Each PCR gene product flanked with coding sequences for specific processing sites was assembled on a Level 0 vector as giant gene modules. Next, promoter modules, terminator modules and giant gene modules were assembled on Level 1 vectors. The *nifHDK*, *nifUS*, *nifFMY*, *nifJVW*, and *nifENB* giant genes were assigned to the Level 1 vectors 1, 2, 3, 8, and 10 respectively. The MBP-TEVp gene module was assigned to Level 1 vector 9. The LEU2 expression cassette with identical *Bsa*I scars was assigned to Level 2 vector B and subsequently used for assembly to provide auxotrophic selection of positive transformants. The restriction enzyme *Xba*I was used for linearization of the plasmids prior to transformation. (This panel relates to Fig. 1 and Fig. 4). (B) Assembly of the violacein biosynthesis and isobutanol biosynthetic pathways for expression in *S. cerevisiae* mitochondria. (This panel relates to Fig. 5).

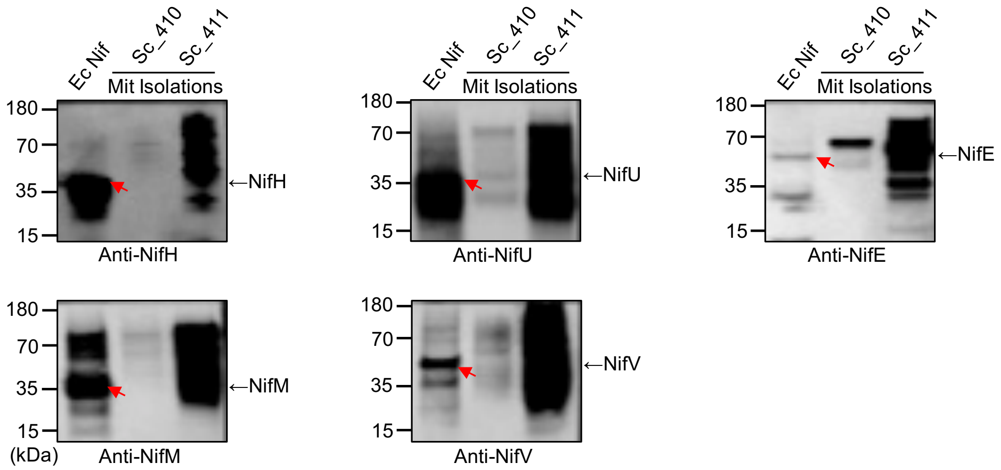

Fig. S3. Long term exposure of Fig. 1*B*. Ec Nif, indicates protein samples prepared from *E. coli* cells carrying the reconstituted operon-based *nif* system. “Mit Isolations”, indicates protein samples prepared from mitochondrial extracts.

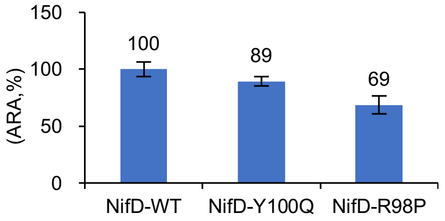

Fig. S4. Acetylene reduction assay (ARA) of NifD and its variants in the operon-based Nif system in *E. coli.* The activity of the *nif* system carrying wild type NifD was assigned as 100%. Relative activities for *nif* systems carrying NifD variants are shown above the bar.

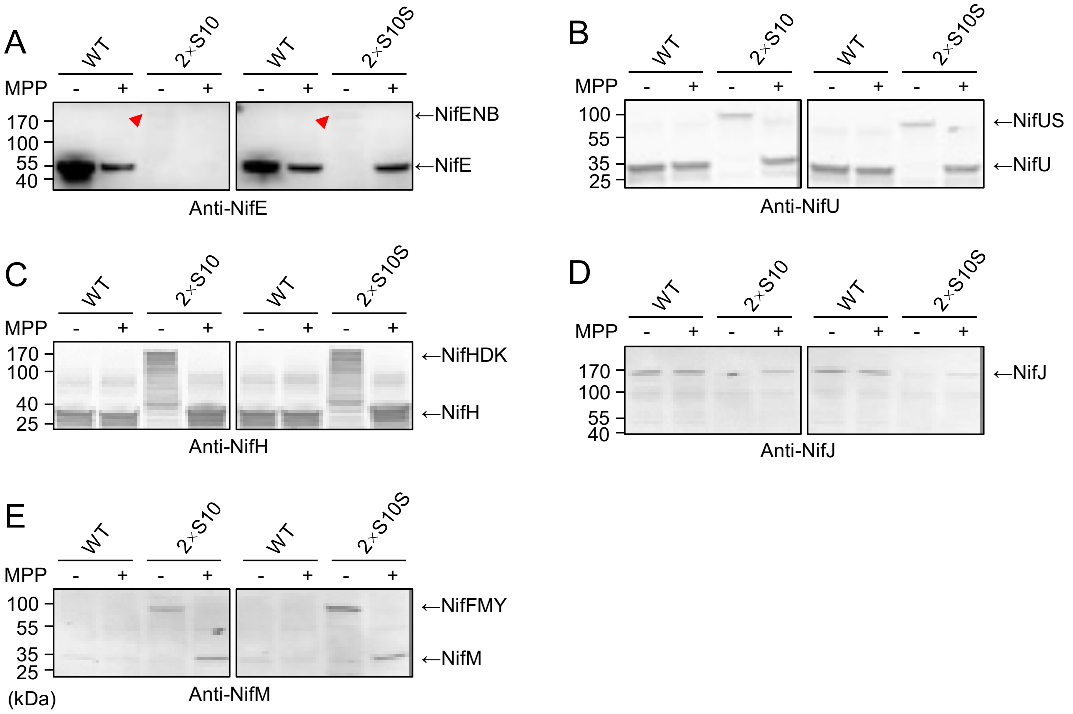

Fig. S5. Immunoblotting of S10 and S10S linked Nif polyproteins expressed in *E. coli*. In each case, a single MPP based giant gene was co-transformed with a plasmid containing the remaining *nif* genes in the operon-based system and grown under nitrogen-fixing conditions to assay acetylene reduction as in Figure 3B. Samples were immediately collected after the acetylene reduction assay and subjected to immunoblotting with Nif protein specific antibodies. "2× S10" indicates that dual S10 sites (RGGGRRAFHT) were present and "2× S10S" that dual S10S sequences (RGGGRRAFST) were present. WT, indicates the complete operon-based *nif* system. "-" indicates that the MPP coding sequence was absent in the strain and "+" that expression of MPP was induced with 100 μM of IPTG. Giant genes tested were (A) *nifEiĩN~B* or *nifEššN~B* (B) *nifUiĩS* or *nifUššS* (C) *nifHiĩDiĩK* or *nifHššDššK* (D) *nifJiĩViĩW or nifJššVššW* (E) *nifFiĩMiĩY* or *nifFššMššY*. Details of the plasmids used in here are provided in Dataset_S01. (This figure relates to Fig. 3)

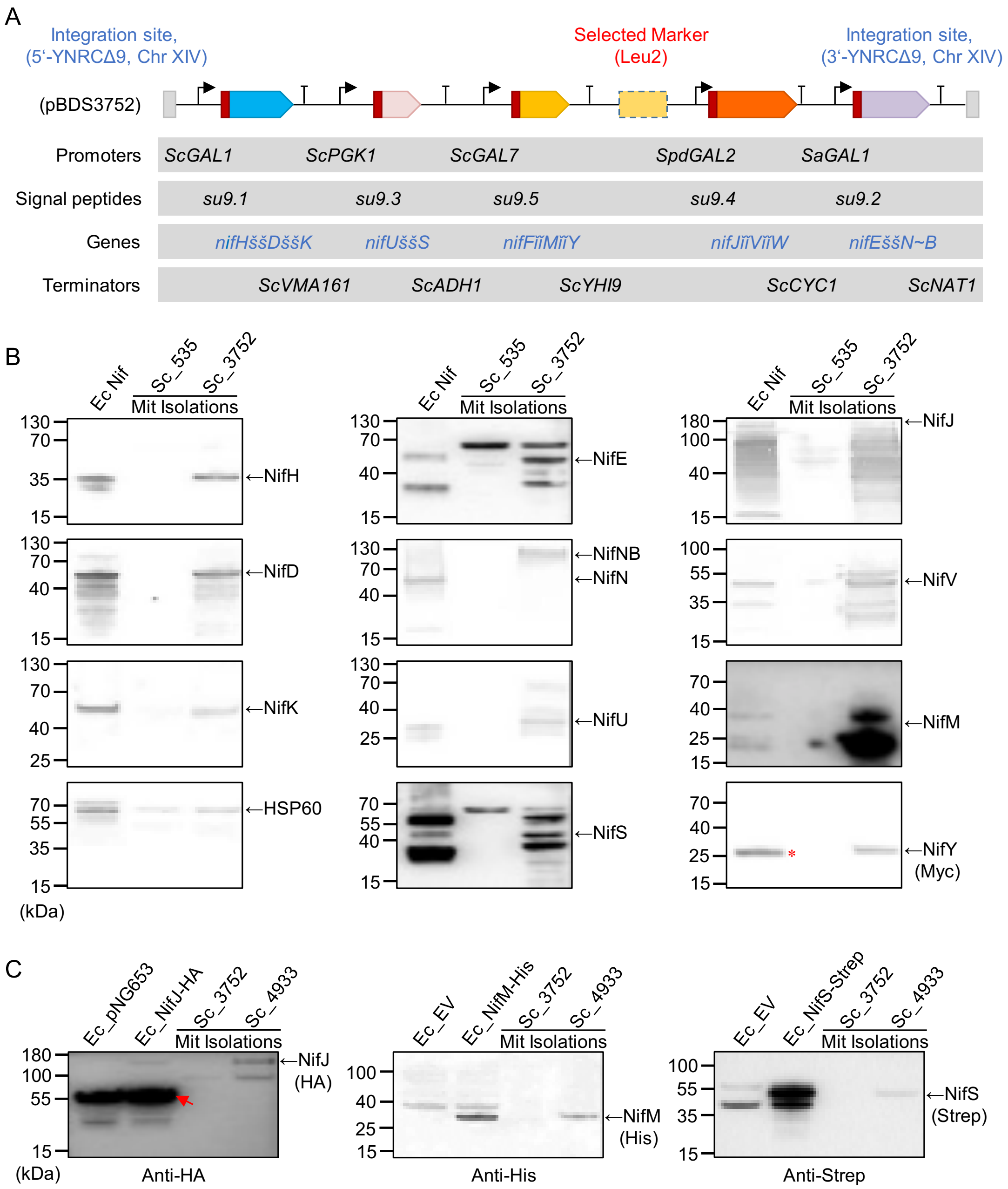

Fig. S6. Details of the MPP-based polyprotein assembly encoded on construct pBDS3752 and immunoblotting of proteins expressed in mitochondria by the corresponding Sc_3752 strain. (A) Schematic diagram showing the genetic parts present on pBDS3752. Giant genes are highlighted in blue and the symbol "ïï" and "šš" are used to represent twofold of MPP sites S10 and S10S respectively. The selection marker Leu2 (in red above the figure) was used for auxotrophic selection of the transformants. Constructs were integrated into the YNRCΔ9 loci on chromosome XIV of the yeast genome with ~600 bp 5'YNRCΔ9 and 3'YNRCΔ9 homology arms (Dataset_S01). (B) Immunoblotting of strains indicated above each panel with Nif specific antibodies. Ec Nif indicates protein samples prepared from *E. coli* cells carrying the reconstituted operon-based *nif* system; Sc_535 is a yeast strain transformed with the empty vector pBDS535, used as a negative control. The red colored star in the bottom right-hand panel indicates the protein sample prepared from *E. coli* cells which express the Myc labeled NifY protein. (C) Immunoblotting of NifJ, NifM, and NifS proteins with antibodies specific to different tags. Ec_pNG653, indicates protein samples

prepared from *E. coli* cells carrying the complete MPP based polyprotein; Ec_NifJ-HA, indicates protein samples prepared from *E. coli* cells carrying the complete MPP based polyprotein with NifJ replaced by HA tag labeled NifJ. The ~55 kDa bands from the *E. coli* samples are the HA-tag labeled beta subunit of MPP from yeast (marked with a red arrow). Ec_Ev, indicates protein samples prepared from *E. coli* cells carrying an empty vector; Ec_NifM-His, and Ec_NifS-Strep indicate extracts prepared from *E. coli* cells which express His labeled NifM, and Strep labeled NifS proteins respectively. "Mit Isolations" indicates protein samples prepared from mitochondrial extracts. Detailed information for plasmids used in this figure are provided in Dataset_S01. (This figure relates to Figure 4).

A

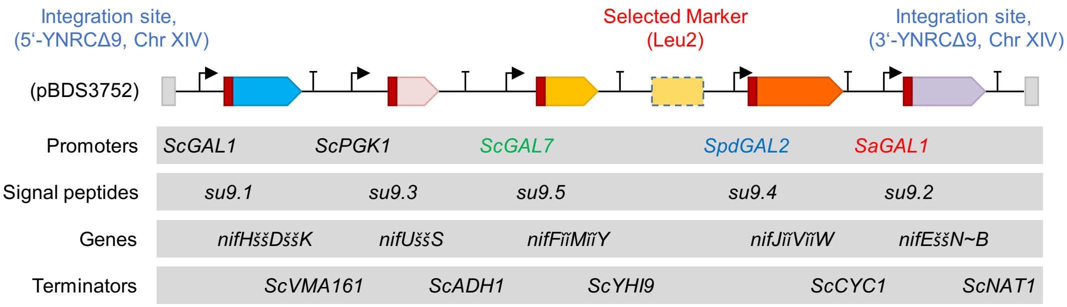

B

Plasmid No.	Promoter	Relative strength
pBDS3752	P_{SaGAL1}	100%
pNG500	$P_{SaGAL1.M11}$	40%
pNG501	$P_{SaGAL1.M4}$	20%
pNG502	$P_{SaGAL1.M1}$	6%

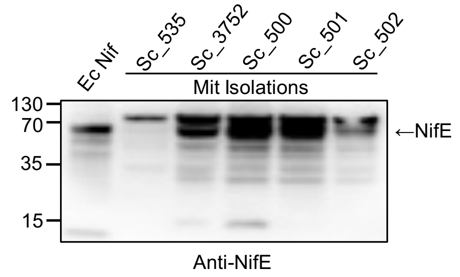

C

Plasmid No.	Promoter	Relative strength
pBDS3752	$P_{SpdGAL2}$	100%
pNG497	$P_{SpdGAL2.M8}$	41%
pNG498	$P_{SpdGAL2.M12}$	22%
pNG499	$P_{SpdGAL2.M14}$	7%

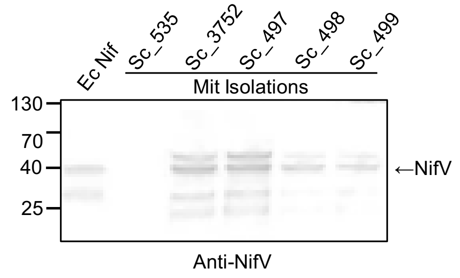

D

Plasmid No.	Promoter	Relative strength
pBDS3752	P_{ScGAL7}	100%
pNG528	$P_{ScGAL7.M15}$	22%
pNG529	$P_{ScGAL7.M5}$	10%
pNG530	$P_{ScGAL7.M4}$	6%

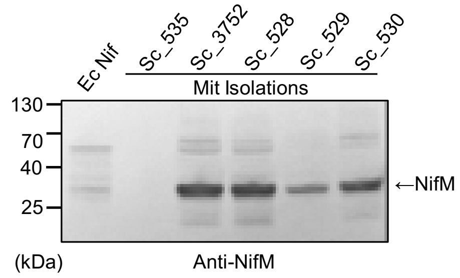

Fig. S7. Expression level optimization for the *nifENB*, *nifJVW*, and *nifFMY* groups in *S. cerevisae.* (A) Schematic diagram showing the parts present on plasmid pBDS3752. Derivatives of this plasmid with variant promoters driving expression of either the ENB polyprotein (highlighted in red), the JVW polyprotein (highlighted in blue) or the FMY polyprotein (highlighted in green) and their relative promoter strengths are listed on the left-hand tables in panels (B), (C) and (D) respectively. Detailed information for each plasmid is listed in Dataset_S01 and methods for measuring promoter strength are provided in the Supplementary Materials and Methods. Immunoblots of mitochondrial extracts from the corresponding yeast strains using antibodies against NifE, NifV and NifM are shown on the right-hand side of panels C, D and E respectively Ec Nif, indicates protein samples prepared from *E. coli* cells carrying the reconstituted operon-based *nif* system; Sc_535, indicates protein samples extracted from mitochondria of strain W303-1a transformed with empty vector. "Mit isolations" indicates extraction from mitochondria. (Related to Fig. 4).

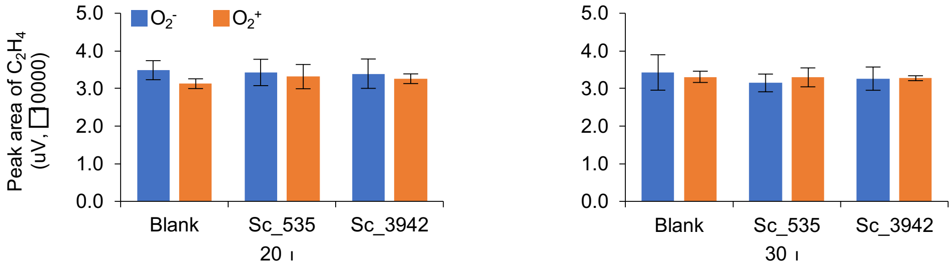

Fig. S8. Acetylene reduction assay of the Sc_3942 strain. One-way ANOVA showed no significant differences amongst the samples (P-value in each group > 0.05). Blank, indicates medium without inoculating yeast cells. Sc_535 indicates *S. cerevisiae* transformed with the empty vector pNG535, assigned as negative control. Sc_3942 is the yeast strain transformed with construct pBDS3942 (carrying the expression optimized MPP based Nif polyprotein system). Detailed strain information is provided in the Dataset_S01. (Related to Fig. 4)

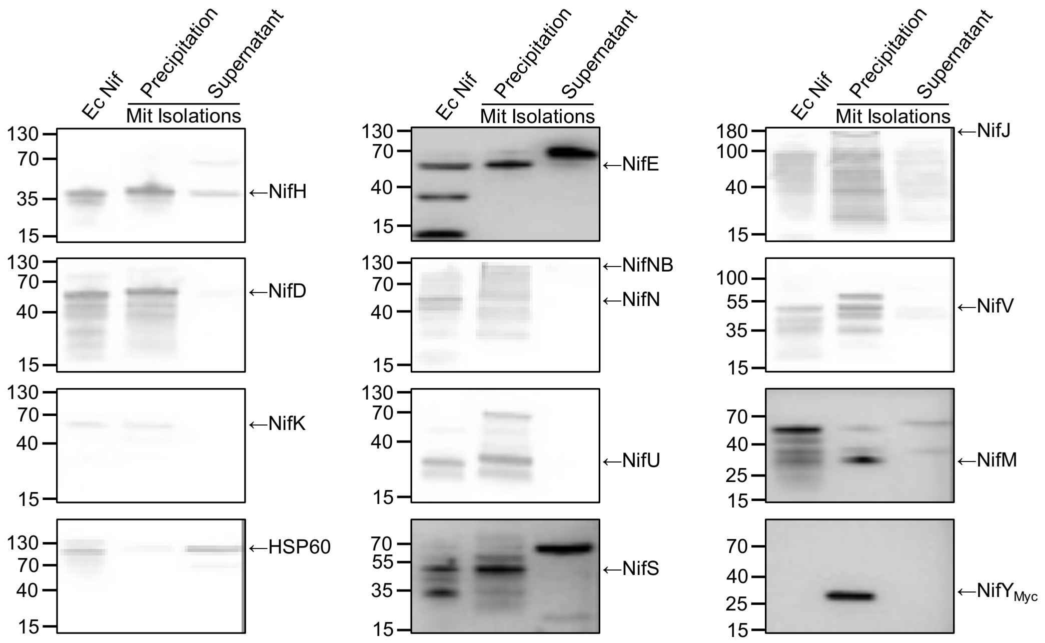

Fig. S9. Solubility of Nif proteins extracted from the Sc_3942 strain. Ec Nif, indicates protein samples prepared from *E. coli* cells carrying the reconstituted operon-based *nif* system. Precipitation and supernatant represent the insoluble and soluble fraction of proteins isolated from mitochondria respectively. “Mit Isolations”, indicates protein samples prepared from mitochondrial extracts. HSP60 was used as internal reference. (Related to Fig. 4)

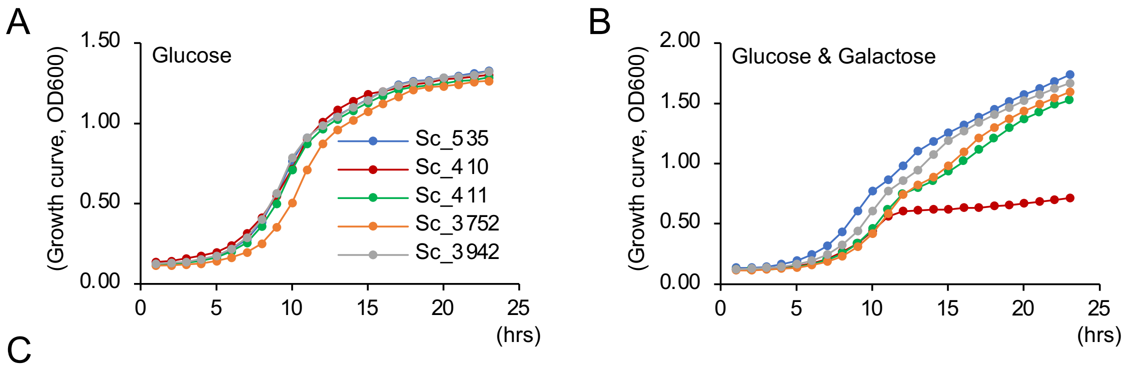

C

Strain	Genotype	Relative Maximum growth rate (%)[a]	
		Glucose	Glucose & Galactose
Sc_535	Empty vector	100 ± 2	100 ± 1
Sc_3752	MPP based polyprotein ver 1.0	100 ± 0[b]	91 ± 5
Sc_3942	MPP based polyprotein ver 2.0	100 ± 2	97 ± 1
Sc_410	TEVp based polyprotein (TEVp$^+$)	94 ± 3	64 ± 3
Sc_411	TEVp based polyprotein (TEVp$^-$)	100 ± 2	89 ± 1

Fig. S10. Comparative growth analysis of yeast strains carrying MPP-based and TEV-based Nif polyprotein systems. (A) growth curves of strains grown in YPD (final concentration of glucose was 2%). (B) growth curves of strains grown in YPDG (final concentrations of glucose and galactose were 0.4% and 1.6% respectively) (C) Table showing the strain information and the maximum growth rate for each strain in panels (A) and (B). [a.] The maximum growth rate of the Sc_535 strain was assigned as 100%. [b.] Indicates SD values lower than 0.5. Detailed strain information is provided in Dataset_S01.

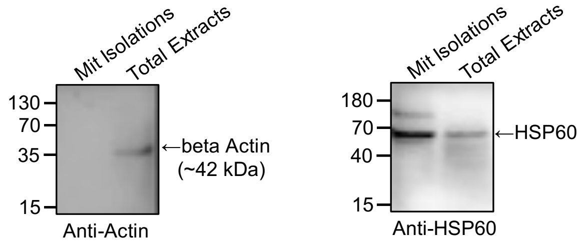

Fig. S11. Immunoblotting of cytosolic beta actin to validate the purity of isolated mitochondria. “Mit Isolations”, indicates protein samples prepared from mitochondrial extracts of yeast strain Sc_535 which carrying an empty vector, “Total Extracts”, indicates total protein samples prepared from the same yeast cells.
