# Supplementary material for: Organelle-dependent polyprotein designs enable stoichiometric expression of nitrogen fixation components targeted to mitochondria
Source: Proc Natl Acad Sci U S A. 2023 Aug 16;120(34):e2305142120. doi: 10.1073/pnas.2305142120 (PMC10450427; doi:10.1073/pnas.2305142120)
Supplement: Supplementary file 3 — Dataset S02 (PDF) [file pnas.2305142120.sd02.pdf]

Complete sequences of construct pNG653

```
     source          1..27299
                     /organism="synthetic DNA construct"
                     /mol_type="other DNA"
     rep_origin      46..591
                     /direction=RIGHT
                     /note="p15A ori"
                     /note="Plasmids containing the medium-copy-number p15A
                     origin of replication can be propagated in E. coli cells
                     that contain a second plasmid with the ColE1 origin."
     promoter        1117..1219
                     /note="cat promoter"
                     /note="promoter of the E. coli cat gene"
     CDS             1220..1879
                     /codon_start=1
                     /gene="cat"
                     /product="chloramphenicol acetyltransferase"
                     /note="CmR"
                     /note="confers resistance to chloramphenicol"

/translation="MEKKITGYTTVDISQWHRKEHFEAFQSVAQCTYNQTVQLDITAFL
KTVKKNKHKFYPAFIHILARLMNAHPEFRMAMKDGELVIWDSVHPCYTVFHEQTETFSS
LWSEYHDDFRQFLHIYSQDVACYGENLAYFPKGFIENMFFVSANPWVSFTSFDLNVANM
DNFFAPVFTMGKYYTQGDKVLMPLAIQVHHAVCDGFHVGRMLNELQQYCDEWQGGA"
     CDS             complement(2913..4103)
                     /codon_start=1
                     /gene="tet"
                     /product="tetracycline efflux protein"
                     /note="TcR"
                     /note="confers resistance to tetracycline"

/translation="MKSNNALIVILGTVTLDAVGIGLVMPVLPGLLRDIVHSDSIASHY
GVLLALYALMQFLCAPVLGALSDRFGRRPVLLASLLGATIDYAIMATTPVLWILYAGRI
VAGITGATGAVAGAYIADITDGEDRARHFGLMSACFGVGMVAGPVAGGLLGAISLHAPF
LAAAVLNGLNLLLGCFLMQESHKGERRPMPLRAFNPVSSFRWARGMTIVAALMTVFFIM
QLVGQVPAALWVIFGEDRFRWSATMIGLSLAVFGILHALAQAFVTGPATKRFGEKQAII
AGMAADALGYVLLAFATRGWMAFPIMILLASGGIGMPALQAMLSRQVDDDHQGQLQGSL
AALTSLTSITGPLIVTAIYAASASTWNGLAWIVGAALYLVCLPALRRGAWSRATST"
     promoter        complement(4150..4184)
                     /note="tet promoter"
                     /note="E. coli promoter for tetracycline efflux protein
                     gene"
     terminator      4257..4276
                     /note="Terminator-nifB"
```

```
     CDS             complement(4314..4343)
                     /codon_start=1
                     /product="13 tandem Myc epitope tags"
                     /note="Myc"
                     /translation="EQKLISEEDL"
     CDS             complement(4344..5003)
                     /codon_start=1
                     /note="nifY"

/translation="MSDNDTLFWRMLALFQSLPDLQPAQIVDWLAQESGETLTPERLAT
LTQPQLAASFPSATAVMSPARWSRVMASLQGALPAHLRIVRPAQRTPQLLAAFCSQDGL
VINGHFGQGRLFFIYAFDEQGGWLYDLRRYPSAPHQQEANEVRARLIEDCQLLFCQEIG
GPAAARLIRHRIHPMKAQPGTTIQAQCEAINTLLAGRLPPWLAKRLNRDNPLEERVF"
     CDS             complement(5004..5066)
                     /codon_start=1
                     /note="su9_9AA_MY"
                     /translation="RGGGRRAFHTRGGGRRAFHT"
     CDS             complement(5067..5864)
                     /codon_start=1
                     /note="nifM"

/translation="MNPWQRFARQRLARSRWNRDPAALDPADTPAFEQAWQRQCHMEQT
IVARVPEGDIPAALLENIAASLAIWLDEGDFAPPERAAIVRHHARLELAFADIARQAPQ
PDLSTVQAWYLRHQTQFMRPEQRLTRHLLLTVDNDREAVHQRILGLYRQINASRDAFAP
LAQRHSHCPSALEEGRLGWISRGLLYPQLETALFSLAENALSLPIASELGWHLLWCEAI
RPAAPMEPQQALESARDYLWQQSQQRHQRQWLEQMISRQPGLCG"
     CDS             complement(5865..5927)
                     /codon_start=1
                     /note="su9_9AA_FM"
                     /translation="RGGGRRAFHTRGGGRRAFHT"
     CDS             complement(5928..6455)
                     /codon_start=1
                     /note="nifF"

/translation="MANIGIFFGTDTGKTRKIAKMIHKQLGELADAPVNINRTTLDDFM
AYPVLLLGTPTLGDGQLPGLEAGCESESWSEFISGLDDASLKGKTVALFGLGDQRGYPD
NFVSGMRPLFDALSARGAQMIGSWPNEGYEFSASSALEGDRFVGLVLDQDNQFDQTEAR
LASWLEEIKRTVL"
     terminator      6845..6876
                     /note="Terminator-nifJ"
     CDS             complement(6926..7186)
                     /codon_start=1
                     /note="nifW"

/translation="MMEWFYQIPGVDELRSAESFFQFFAVPYQPELLGRCSLPVLATFH
```

RKLRAEVPLQNRLEDNDRAPWLLARRLLAESYQQQFQESGT"
CDS complement(7187..7249)
/codon_start=1
/note="su9_10AA_VW"
/translation="RGGGRRAFHTRGGGRRAFHT"
CDS complement(7250..8389)
/codon_start=1
/note="nifV"

/translation="MERVLINDTTLRDGEQSPGVAFRTSEKVAIAEALYAAGITAMEVG
TPAMGDEEIARIQLVRRQLPDATLMTWCRMNALEIRQSADLGIDWVDISIPASDKLRQY
KLREPLAVLLERLAMFIHLAHTLGLKVCIGCEDASRASGQTLRAIAEVAQQCAAARLRY
ADTVGLLDPFTTAAQISALRDVWSGEIEMHAHNDLGMATANTLAAVSAGATSVNTTVLG
LGERAGNAALETVALGLERCLGVETGVHFSALPALCQRVAEAAQRAIDPQQPLVGELVF
THESGVHVAALLRDSESYQSIAPSLMGRSYRLVLGKHSGRQAVNGVFDQMGYHLNAAQI
NQLLPAIRRFAENWKRSPKDYELVAIYDELCGESALRARG"
CDS complement(8390..8452)
/codon_start=1
/note="su9_10AA_JV"
/translation="RGGGRRAFHTRGGGRRAFHT"
CDS complement(8453..11965)
/codon_start=1
/note="nifJ"

/translation="MSGKMKTMDGNAAAAWISYAFTEVAAIYPITPSTPMAENVDEWAA
QGKKNLFGQPVRLMEMQSEAGAAGAVHGALQAGALTTTYTASQGLLLMIPNMYKIAGEL
LPGVFHVSARALATNSLNIFGDHQDVMAVRQTGCAMLAENNVQQVMDLSAVAHLAAIKG
RIPFVNFFDGFRTSHEIQKIEVLEYEQLATLLDRPALDSFRRNALHPDHPVIRGTAQNP
DIYFQEREAGNRFYQALPDIVESYMTQISALTGREYHLFNYTGAADAERVIIAMGSVCD
TVQEVVDTLNAAGEKVGLLSVHLFRPFSLAHFFAQLPKTVQRIAVLDRTKEPGAQAEPL
CLDVKNAFYHHDDAPLIVGGRYALGGKDVLPNDIAAVFDNLNKPLPMDGFTLGIVDDVT
FTSLPPRQQTLAVSHDGITACKFWGMGSDGTVGANKSAIKIIGDKTPLYAQAYFSYDSK
KSGGITVSHLRFGDRPINSPYLIHRADFISCSQQSYVERYDLLDGLKPGGTFLLNCSWS
DAELEQHLPVGFKRYLARENIHFYTLNAVDIARELGLGGRFNMLMQAAFFKLAAIIDPQ
TAADYLKQAVEKSYGSKGAAVIEMNQRAIELGMASLHQVTIPAHWATLDEPAAQASAMM
PDFIRDILQPMNRQCGDQLPVSAFVGMEDGTFPSGTAAWEKRGIALEVPVWQPEGCTQC
NQCAFICPHAAIRPALLNGEEHDAAPVGLLSKPAQGAKEYHYHLAISPLDCSGCGNCVD
ICPARGKALKMQSLDSQRQMAPVWDYALALTPKSNPFRKTTVKGSQFETPLLEFSGACA
GCGETPYARLITQLFGDRMLIANATGCSSIWGASAPSIPYTTNHRGHGPAWANSLFEDN
AEFGLGMMLGGQAVRQQIADDMTAALALPVSDELSDAMRQWLAKQDEGEGTRERADRLS
ERLAAEKEGVPLLEQLWQNRDYFVRRSQWIFGGDGWAYDIGFGGLDHVLASGEDVNILV
FDTEVYSNTGGQSSKSTPVAAIAKFAAQGKRTRKKDLGMMAMSYGNVYYVAQVAMGADKD
QTLRAIAEAEAWPGPSLVIAYAACINHGLKAGMRCSQREAKRAVEAGYWHLWRYHPQRE
AEGKTPFMLDSEEPEESFRDFLLGEVRYASLHKTTPHLADALFSRTEEDARARFAQYRR
LAGEE"

CDS 12666..14153
/codon_start=1
/note="nifL"

/translation="MTLNMMLDNAVPEAIAGALTQQHPGLFFTMVEQASVAISLTDARA
NITYANPAFCRQTGYSLAQLLNQNPRLLASSQTPREIYQEMWQTLLQRQPWRGQLINQA
RDGGLYLVDIDITPVLNPQGELEHYLAMQRDISVSYTLEQRLRNHMTLMEAVLNNIPAA
VVVVDEQDRVVMDNLAYKTFCADCGGKELLVELQVSPRKMGPGAEQILPVVVRGAVRWL
SVTCWALPGVSEEASRYFVDSAPARTLMVIADCTQQRQQQEQGRLDRLKQQMTAGKLLA
AIRESLDAALIQLNCPINMLAAARRLNGEGSGNVALDAAWREGEEAMARLQRCRPSLEL
ESNAVWPLQPFFDDLYALYRTRFDDRARLQVDMASPHLVGFGQRTQLLACLSLWLDRTL
ALAAELPSVPLEIELYAEEDEGWLSLYLNDNVPLLQVRYAHSPDALNSPGKGMELRLIQ
TLVAYHRGAIELASRPQGGTSLVLRFPLFNTLTGGEQ"
CDS 14150..15727
/codon_start=1
/note="nifA"
/translation="MIHKSDSDTTVRRFDLSQQFTAMQRISVVLSRATEASKTLQEVLS
VLHNDAFMQHGMICLYDSQQEILSIEALQQTEDQTLPGSTQIRYRPGEGLVGTVLAQGQ
SLVLPRVADDQRFLDRLSLYDYDLPFIAVPLMGPHSRPIGVLAAHAMARQEERLPACTR
FLETVANLIAQTIRLMILPTSAAQAPQQSPRIERPRACTPSRGFGLENMVGKSPAMRQI
MDIIRQVSRWDTTVLVRGESGTGKELIANAIHHNSPRAAAAFVKFNCAALPDNLLESEL
FGHEKGAFTGAVRQRKGRFELADGGTLFLDEIGESSASFQAKLLRILQEGEMERVGGDE
TLRVNVRIIAATNRHLEEEVRLGHFREDLYYRLNVMPIALPPLRERQEDIAELAHFLVR
KIAHSQGRTLRISDGAIRLLMEYSWPGNVRELENCLERSAVLSESGLIDRDVILFNHRD
NPPKALASSGPAEDGWLDNSLDERQRLIAALEKAGWVQAKAARLLGMTPRQVAYRIQIM
DITMPRLS"
terminator 15806..15832
/note="Terminator2-nifH"
CDS 15992..17362
/codon_start=1
/note="nifE"

/translation="MKGNEILALLDEPACEHNHKQKSGCSAPKPGATAGGCAFDGAQIT
LLPIADVAHLVHGPIGCAGSSWDNRGSASSGPTLNRLGFTTDLNEQDVIMGRGERRLFH
AVRHIVTRYHPAAVFIYNTCVPAMEGDDLEAVCQAAQTATGVPVIAIDAAGFYGSKNLG
NRLAGDVMVKRVIGQREPAPWPESTLFAPEQRHDIGLIGEFNIAGEFWHIQPLLDELGI
RVLGSLSGDGRFAEIQTMHRAQANMLVCSRALINVARALEQRYGTPWFEGSFYGIRATS
DALRQLAALLGDDDLRQRTEALIAREEQAAELALQPWREQLRGRKALLYTGGVKSWSVV
SALQDLGMTVVATGTRKSTEEDKQRIRELMGEEAVMLEEGNARTLLDVVYRYQADLMIA
GGRNMYTAYKARLPFLDINQEREHAFAGYQGIVTLARQLCQTINSPIWPQTHSRAPWR"
CDS 17363..17425
/codon_start=1
/note="su9_10AA_2F_EN"
/translation="RGGGRRAFSTRGGGRRAFST"
CDS 17426..18808

/codon_start=1
/note="nifN"

/translation="MADIFRTDKPLAVSPIKTGQPLGAILASLGIEHSIPLVHGAQGCS
AFAKVFFIQHFHDPVPLQSTAMDPTSTIMGADGNIFTALDTLCQRNNPQAIVLLSTGLS
EAQGSDISRVVRQFREEYPRHKGVAILTVNTPDFYGSMENGFSAVLESVIEQWVPPAPR
PAQRNRRVNLLVSHLCSPGDIEWLRRCVEAFGLQPIILPDLAQSMDGHLAQGDFSPLTQ
GGTPLRQIEQMGQSLCSFAIGVSLHRASSLLAPRCRGEVIALPHLMTLERCDAFIHQLA
KISGRAVPEWLERQRGQLQDAMIDCHMWLQGQRMAIAAEGDLLAAWCDFANSQGMQPGP
LVAPTGHPSLRQLPVERVVPGDLEDLQTLLCAHPADLLVANSHARDLAEQFALPLVRAG
FPLFDKLGEFRRVRQGYSGMRDTLFELANLIRERHHHLAHYRSPLRQNPESSLSTGGAY
AAD"
CDS 18809..18853
/codon_start=1
/note="3GS"
/translation="GGGGSGGGGSGGGGS"
CDS 18854..20257
/codon_start=1
/note="nifB"

/translation="TSCSSFSGGKACRPADDSALTPLVADKAAAHPCYSRHGHHRFARM
HLPVAPACNLQCNYCNRKFDCSNESRPGVSSTLLTPEQAVVKVRQVAQAIPQLSVVGIA
GPGDPLANIARTFRTLELIREQLPDLKLCLSTNGLMLPDAVDRLLDVGVDHVTVTINTL
DAEIAAQIYAWLWLDGERYSGREAGEILIARQLEGVRRLTAKGVLVKINSVLIPGINDS
GMADVSRALRASGAFIHNIMPLIARPEHGTVFGLNGQPEPDAETLAATRSRCGEVMPQM
THCHQCRADAIGMLGEDRSQQFTQLPAPESLPAWLPILHQRAQLHASIATRGESEADDA
CLVAVASSRGDVIDCHFGHADRFYIYSLSAAGMVLVNERFTPKYCQGRDDCEPQDNAAR
FAAILELLADVKAVFCVRIGHTPWQQLEQEGIEPCVDGAWRPVSEVLPAWWQQRRGSWP
AALPHKGVA"
terminator 20280..20310
/note="Terminator-nifL"
promoter 20350..20612
/note="PnifH"
CDS 20613..21491
/codon_start=1
/note="nifH"

/translation="MTMRQCAIYGKGGIGKSTTTQNLVAALAEMGKKVMIVGCDPKADS
TRLILHAKAQNTIMEMAAEVGSVEDLELEDVLQIGYGDVRCAESGGPEPGVGCAGRGVI
TAINFLEEEGAYEDDLDFVFYDVLGDVVCGGFAMPIRENKAQEIYIVCSGEMMAMYAAN
NISKGIVKYAKSGKVRLGGLICNSRQTDREDELIIALAEKLGTQMIHFVPRDNIVQRAE
IRRMTVIEYDPACKQANEYRTLAQKIVNNTMKVVPTPCTMDELESLLMEFGIMEEEDTS
IIGKTAAEENAA"
CDS 21492..21551
/codon_start=1

/note="su9_10AA_2F_HD"
/translation="RGGGRRAFSTRGGGRRAFST"
CDS 21552..23000
/codon_start=1
/note="nifD"

/translation="MMTNATGERNLALIQEVLEVFPETARKERRKHMMVSDPEMESVGK
CIISNRKSQPGVMTVRGCAYAGSKGVVFGPIKDMAHISHGPVGCGQYSRAGRRNQYTGV
SGVDSFGTLNFTSDFQERDIVFGGDKKLSKLIEEMELLFPLTKGITIQSECPVGLIGDD
ISAVANASSKALDKPVIPVRCEGFRGVSQSLGHHIANDVVRDWILNNREGQPFETTPYD
VAIIGDYNIGGDAWASRILLEEMGLRVVAQWSGDGTLVEMENTPFVKLNLVHCYRSMNY
IARHMEEKHQIPWMEYNFFGPTKIAESLRKIADQFDDTIRANAEAVIARYEGQMAAIIA
KYRPRLEGRKVLLYMGGLRPRHVIGAYEDLGMEIIAAGYEFAHNDDYDRTLPDLKEGTL
LFDDASSYELEAFVKALKPDLIGSGIKEKYIFQKMGVPFRQMHSWDYSGPYHGYDGFAI
FARDMDMTLNNPAWNELTAPWLKSA"
misc_feature 21849..21851
/note="Y100Q"
CDS 23001..23060
/codon_start=1
/note="su9_10AA_2F_DK"
/translation="RGGGRRAFSTRGGGRRAFST"
CDS 23061..24623
/codon_start=1
/note="nifK"

/translation="MSQTIDKINSCYPLFEQDEYQELFRNKRQLEEAHDAQRVQEVFAW
TTTAEYEALNFQREALTVDPAKACQPLGAVLCSLGFANTLPYVHGSQGCVAYFRTYFNR
HFKEPIACVSDSMTEDAAVFGGNNNMNLGLQNASALYKPEIIAVSTTCMAEVIGDDLQA
FIANAKKDGFVDSSIAVPHAHTPSFIGSHVTGWDNMFEGFAKTFTADYQGQPGKLPKLN
LVTGFETYLGNFRVLKRMMEQMAVPCSLLSDPSEVLDTPADGHYRMYSGGTTQQEMKEA
PDAIDTLLLQPWQLLKSKKVVQEMWNQPATEVAIPLGLAATDELLMTVSQLSGKPIADA
LTLERGRLVDMMLDSHTWLHGKKFGLYGDPDFVMGLTRFLLELGCEPTVILSHNANKRW
QKAMNKMLDASPYGRDSEVFINCDLWHFRSLMFTRQPDFMIGNSYGKFIQRDTLAKGKA
FEVPLIRLGFPLFDRHHLHRQTTWGYEGAMNIVTTLVNAVLEKLDSDTSQLGKTDYSFD
LVR"
terminator 24643..24661
/note="Terminator1-nifH"
terminator 24760..24786
/note="Terminator2-nifH"
promoter 24877..25113
/note="PnifU"
CDS 25114..25935
/codon_start=1
/note="nifU"

/translation="MWNYSEKVKDHFFNPRNARVVDNANAVGDVGSLSCGDALRLMLRV
DPQSEIIEEAGFQTFGCGSAIASSSALTELIIGHTLAEAGQITNQQIADYLDGLPPEKM
HCSVMGQEALRAAIANFRGESLEEEHDEGKLICKCFGVDEGHIRRAVQNNGLTTLAEVI
NYTKAGGGCTSCHEKIELALAEILAQQPQTTPAVASGKDPHWQSVVDTIAELRPHIQAD
GGDMALLSVTNHQVTVSLSGSCSGCMMTDMTLAWLQQKLMERTGCYMEVVAA"
     CDS   25936..25995
                     /codon_start=1
                     /note="su9_10AA_US"
                     /translation="RGGGRRAFSTRGGGRRAFST"
     CDS   25996..27198
                     /codon_start=1
                     /note="nifS"

/translation="MKQVYLDNNATTRLDPMVLEAMMPFLTDFYGNPSSIHDFGIPAQA
ALERAHQQAAALLGAEYPSEIIFTSCATEATATAIASAIALLPERREIITSVVEHPATL
AACEHLERQGYRIHRIAVDSEGALDMAQFRAALSPRVALVSVMWANNETGVLFPIGEMA
ELAHEQGALFHCDAVQVVGKIPIAVGQTRIDMLSCSAHKFHGPKGVGCLYLRRGTRFRP
LLRGGHQEYGRRAGTENICGIVGMGAACELANIHLPGMTHIGQLRNRLEHRLLASVPSV
MVMGGGQPRVPGTVNLAFEFIEGEAILLLLNQAGIAASSGSACTSGSLEPSHVMRAMNI
PYTAAHGTIRFSLSRYTREKEIDYVVATLPPIIDRLRALSPYWQNGKPRPADAVFTPVY
G"
     terminator   27219..27241
                     /note="Terminator-nifUF"
ORIGIN
        1 atgtcttcac tagaaatatt ttatctgatt aataagatga tcttcttgag atcgttttgg
       61 tctgcgcgta atctcttgct ctgaaaacga aaaaaccgcc ttgcagggcg gtttttcgaa
      121 ggttctctga gctaccaact ctttgaaccg aggtaactgg cttggaggag cgcagtcacc
      181 aaaacttgtc ctttcagttt agccttaacc ggcgcatgac ttcaagacta actcctctaa
      241 atcaattacc agtggctgct gccagtggtg cttttgcatg tctttccggg ttggactcaa
      301 gacgatagtt accggataag gcgcagcggt cggactgaac ggggggttcg tgcatacagt
      361 ccagcttgga gcgaactgcc tacccggaac tgagtgtcag gcgtggaatg agacaaacgc
      421 ggccataaca gcggaatgac accggtaaac cgaaaggcag gaacaggaga gcgcacgagg
      481 gagccgccag ggggaaacgc ctggtatctt tatagtcctg tcgggtttcg ccaccactga
      541 tttgagcgtc agatttcgtg atgcttgtca ggggggcgga gcctatggaa aaacggcttt
      601 gccgcggccc tctcacttcc ctgttaagta tcttcctggc atcttccagg aaatctccgc
      661 cccgttcgta agccatttcc gctcgccgca gtcgaacgac cgagcgtagc gagtcagtga
      721 gcgaggaagc ggaatatatc ctgtatcaca tattctgctg acgcaccggt gcagcctttt
      781 ttctcctgcc acatgaagca cttcactgac accctcatca gtgccaacat agtaagccag
      841 tatacactcc gcttgcgctg atgtccggcg gtgcttttgc cgttacgcac caccccgtca
      901 gtagctgaac aggagggaca gctgatagaa acagaagcca ctggagcacc tcaaaaacac
      961 catcatacac taaatcagta agttggcagc atcacccgac gcactttgcg ccgaataaat
     1021 acctgtgacg gaagatcact tcgcagaata aataaatcct ggtgtccctg ttgataccgg
     1081 gaagccctgg gccaactttt ggcgaaaatg agacgttgat cggcacgtaa gaggttccaa
     1141 ctttcaccat aatgaaataa gatcactacc gggcgtattt tttgagttat cgagattttc
     1201 aggagctaag gaagctaaaa tggagaaaaa aatcactgga tataccaccg ttgatatatc

1261 ccaatggcat cgtaaagaac attttgaggc atttcagtca gttgctcaat gtacctataa
1321 ccagaccgtt cagctggata ttacggcctt tttaaagacc gtaaagaaaa ataagcacaa
1381 gttttatccg gcctttattc acattcttgc ccgcctgatg aatgctcatc cggaattccg
1441 tatggcaatg aaagacggtg agctggtgat atgggatagt gttcaccctt gttacaccgt
1501 tttccatgag caaactgaaa cgttttcatc gctctggagt gaataccacg acgatttccg
1561 gcagtttcta cacatatatt cgcaagatgt ggcgtgttac ggtgaaaacc tggcctattt
1621 ccctaaaggg tttattgaga atatgttttt cgtctcagcc aatccctggg tgagtttcac
1681 cagttttgat ttaaacgtgg ccaatatgga caacttcttc gcccccgttt tcaccatggg
1741 caaatattat acgcaaggcg acaaggtgct gatgccgctg gcgattcagg ttcatcatgc
1801 cgtctgtgat ggcttccatg tcggcagaat gcttaatgaa ttacaacagt attgcgatga
1861 gtggcagggc ggggcgtaat ttttttaagg cagttattgg tgcccttaaa cgcctggtgc
1921 tacgcctgaa taagtgataa taagcggatg aatggcagaa attcgaaagc aaattcgacc
1981 cggtcgtcgg ttcagggcag ggtcgttaaa tagccgctta tgtctattgc tggtttaccg
2041 gtttattgac taccggaagc agtgtgaccg tgtgcttctc aaatgcctga ggccagtttg
2101 ctcaggctct ccccgtggag gtaataattg acgatatgat catttattct gcctcccaga
2161 gcctgataaa aacggttagc gcttcgttaa tacagatgta ggtgttccac agggtagcca
2221 gcagcatcct gcgatgcaga tccggaacat aatggtgcag ggcgcttgtt tcggcgtggg
2281 tatggtggca ggccccgtgg ccgggggact gttgggcgct gccggcacct gtcctacgag
2341 ttgcatgata aagaagacag tcataagtgc ggcgacgata gtcatgcccc gcgcccaccg
2401 gaaggagcta ccggacagcg gtgcggactg ttgtaactca gaataagaaa tgaggccgct
2461 catggcgttg actctcagtc atagtatcgt ggtatcaccg gttggttcca ctctctgttg
2521 cgggcaactt cagcagcacg taggggactt ccgcgtttcc agactttacg aaacacggaa
2581 accgaagacc attcatgttg ttgctcaggt cgcagacgtt ttgcagcagc agtcgcttca
2641 cgttcgctcg cgtatcggtg attcattctg ctaaccagta aggcaacccc gccagcctag
2701 ccgggtcctc aacgacagga gcacgatcat gcgcacccgt ggccaggacc caacgctgcc
2761 cgagatgcgc cgcgtgcggc tgctggagat ggcggacgcg atggatatgt tctgccaagg
2821 gttggtttgc gcattcacag ttctccgcaa gaattgattg gctccaattc ttggagtggt
2881 gaatccgtta gcgaggtgcc gccggcttcc attcaggtcg aggtggcccg gctccatgca
2941 ccgcgacgca acgcggggag gcagacaagg tatagggcgg cgcctacaat ccatgccaac
3001 ccgttccatg tgctcgccga ggcggcataa atcgccgtga cgatcagcgg tccagtgatc
3061 gaagttaggc tggtaagagc cgcgagcgat ccttgaagct gtccctgatg gtcgtcatct
3121 acctgcctgg acagcatggc ctgcaacgcg ggcatcccga tgccgccgga agcgagaaga
3181 atcataatgg ggaaggccat ccagcctcgc gtcgcgaacg ccagcaagac gtagcccagc
3241 gcgtcggccg ccatgccggc gataatggcc tgcttctcgc cgaaacgttt ggtggcggga
3301 ccagtgacga aggcttgagc gagggcgtgc aagattccga ataccgcaag cgacaggccg
3361 atcatcgtcg cgctccagcg aaagcggtcc tcgccgaaaa tgacccagag cgctgccggc
3421 acctgtccta cgagttgcat gataaagaag acagtcataa gtgcggcgac gatagtcatg
3481 ccccgcgccc accggaagga gctgactggg ttgaaggctc tcaagggcat cggtcgacgc
3541 tctcccttat gcgactcctg cattaggaag cagcccagta gtaggttgag gccgttgagc
3601 accgccgccg caaggaatgg tgcatgcaag gagatggcgc ccaacagtcc cccggccacg
3661 gggcctgcca ccatacccac gccgaaacaa gcgctcatga gcccgaagtg gcgagcccga
3721 tcttccccat cggtgatgtc ggcgatatag gcgccagcaa ccgcacctgt ggcgccggtg
3781 atgccggcca cgatgcgtcc ggcgtagagg atccacagga cgggtgtggt cgccatgatc
3841 gcgtagtcga tagtggctcc aagtagcgaa gcgagcagga ctgggcggcg gccaaagcgg
3901 tcggacagtg ctccgagaac gggtgcgcat agaaattgca tcaacgcata tagcgccagc

3961 agcacgccat agtgactggc gatgctgtcg gaatggacga tatcccgcaa gaggcccggc
4021 agtaccggca taaccaagcc tatgcctaca gcatccaggg tgacggtgcc gaggatgacg
4081 atgagcgcat tgttagattt catacacggt gcctgactgc gttagcattt aactgtgata
4141 aactaccgca ttaaagctag agcttatcga tgataagctg tcaaacatga gaattacaac
4201 ttatatcgta tggggctgac tagaatttaa attaatcaca tttgatgatg tgaaaagccg
4261 gagggaaacc tccggctttt ttgtttgcag cgcccccttc gggaaacggc ttacaaatct
4321 tcctcggaga ttagcttttg ttcaaaaacg cgttcttcca gagggttatc cctgttaagc
4381 cgcttcgcca gccacggcgg caaacggccg gccagcagcg tattgatcgc ctcgcactgt
4441 gcctgaatcg tcgtcccggg ctgcgctttc atcgggtgga tgcgatggcg gatcagccgc
4501 gcggcggcgg gcccgcctat ctcctggcaa aacagcagct gacagtcctc aataagccgg
4561 gcgcgcactt cgttggcctc ctgctggtgg ggggcggagg gatagcggcg cagatcgtac
4621 aaccagccgc cttgttcatc gaacgcgtag ataaaaaaca gtcgtccctg gccgaaatgg
4681 ccgttaatca ccagcccatc ctgggagcaa aatgccgcca gcagctgcgg cgtgcgctgg
4741 gcagggcgaa cgatgcgtaa atgggcgggc agcgcgccct gcaggctcgc catcacccgc
4801 gaccagcgag cgggggacat caccgccgtc gcggagggaa agctggcggc cagctgcggc
4861 tgggtcaggg tcgccagacg ctctggcgtc agcgtctcgc cgctctcctg cgccagccag
4921 tcgacgattt gcgccggctg taggtccggc agagactgaa acagcgccag catacgccag
4981 aatagggtat cgttgtcgga catcgtatga aatgcacgac gaccaccgcc acgggtatgg
5041 aaagcgcgac gaccaccccc gcgcgccccg cacagtcccg gctgacggga aatcatctgt
5101 tccagccact ggcgctgatg gcgctgctgg ctctgctgcc aaagataatc gcgcgcgctc
5161 tccagcgcct gctgcggctc catgggcgcg gcggggcgaa tcgcttcgca ccataaaaga
5221 tgccagccca gttcgctggc gatgggaagg cttagcgcgt tttccgccag tgaaaacagc
5281 gcggtttcga gctgcggata gagcaggcca cggctaatcc agcctaaacg accctcttcc
5341 agcgcgctcg ggcagtggga atggcgctgg gccagcggcg cgaaagcgtc ccgcgaggcg
5401 ttgatttgcc gatacaggcc gaggatccgc tggtgcacgg cttcgcggtc gttatcgacc
5461 gtcagcagta aatggcgggt cagacgctgt tccgggcgca taaactgcgt ctggtggcgc
5521 agataccatg cctgtaccgt ggagagatcc ggctgcggcg cctggcgggc gatatcggcg
5581 aaggcgagtt ccagccgggc gtgatggcgc acgatggcag cgcgctcggg cggcgcaaaa
5641 tccccctcgt cgagccagat ggcaagggag gcagcgatat tctccagcaa cgccgccgga
5701 atatcgcctt cagggacccg cgcgacgatc gtctgctcca tatggcactg gcgttgccag
5761 gcctgttcaa aagccggcgt atcggccgga tccagggccg ccggatcgcg attccagcgg
5821 ctgcgcgcca gccgctgccg ggcaaaacgt tgccacgggt tcatagtgtg aaacgcacgg
5881 cgacccccac cacgggtatg aaaagcacgg cgaccgccac cacgtgccag cacggtgcgt
5941 ttaatctcct caagccagct cgccagacgc gcttcggtct ggtcgaactg gttatcctga
6001 tccagcacca gcccaacaaa gcggtcgcct tccagcgccg aggacgcgct gaattcataa
6061 ccctcatttg gccagctgcc aatcatctgc gcgccgcgcg cgctcagggc gtcgaacagc
6121 gggcgcatcc cgctgacgaa gttgtccgga tagcctctct gatcgccgag gccgaacagc
6181 gccacggttt tccctttcag gctggcgtcg tcgaggccgc tgataaattc gctccatgac
6241 tcgctttcgc atccggcctc cagccccggc agctggccgt cgccgagcgt cggcgtgccc
6301 agcagcagca ccggataggc cataaagtcg tccagcgtcg tgcggttaat gttgaccggg
6361 gcatccgcca gctcgcccag ttgcttatgg atcattttcg cgattttgcg ggttttaccg
6421 gtatcggtgc caaagaaaat accaatgttc gccatgttgc gctcctgtcg gaaaaggggg
6481 ttgaaaatac gcgttctcgc aggggtattg cgaaggctgt gccaggttgc tttgcactac
6541 cgcggcccat ccctgcccca aaacgatcgc ttcagccctc tcccgccgcg cgcggcgggg
6601 ctggcggggc gcttaaaatg caaaaagcgc ctgcttttcc cctaccggat caatgtttct

6661 gcacatcacg ccgataaggg cgcacggttt gcatggttat caccgttcgg aaaacaccgc
6721 ggcgtccctg tcacggtgtc ggacaaattg tcataactgc gacacaggag tttgcgatga
6781 ccctgaaatt taaatactag aagtactcag cgcccacagg ggcgggagaa cttgatttcg
6841 ctgcgccggg aagatttaga cgcgcgcttt cccggcctgc tggatacgct gcttaaggtc
6901 atgcagcagg agaactaaag gccccttatg ttccgctctc ctgaaactgt tgctgatagc
6961 tctccgcgag cagtctgcgc gccagcagcc agggcgcgcg gtcgttatcc tcgagccggt
7021 tttgcagcgg cacctccgcg cggagtttgc gatgaaacgt tgccagcacc ggcaggctgc
7081 agcggccaag cagctcgggc tgataggga cggcgaaaaa ctgaaaaaaa gattcggcgg
7141 agcgaagttc gtccacgccg ggaatttgat aaaaccactc catcatggtg tggaaagcac
7201 gacggccacc gccacgagta tgaaacgcac ggcgacctcc gccgcgggcc cccctcgccc
7261 gcagagcgga ttcaccgcac agctcgtcgt agatagccac cagctcgtaa tctttcgggc
7321 tgcgcttcca gttctcggcg aagcggcgga tggcgggcag cagctggtta atctgcgcgg
7381 cgttgaggtg atagcccatc tggtcaaaaa cgccgttgac cgcctgacgc ccggagtgtt
7441 tgcccagcac cagccggtag ctgcggccca tcagggaagg ggcgatggac tggtagctct
7501 cgctgtcgcg cagcagcgcc gccacgtgga cacctgactc atgggtaaac accagctcgc
7561 cgaccagcgg ctgctgcggg tcgatggcgc gctgcgcggc ttccgcgacc ctctgacaga
7621 gcgcgggcag cgccgaaaaa tgcacgccgg tctccacgcc caggcagcgt tcaaggccca
7681 gcgcgacggt ttccagcgcc gcgttgcccg cccgctcgcc gagaccgagg accgtcgtat
7741 tcacgctggt ggccccggcg cttaccgccg ccagcgtatt ggcggtcgcc atacccagat
7801 cgttatgggc atgcatttcg atttcgccgg accagacgtc gcgcagggcc gagatttgcg
7861 ccgcggtggt aaaagggtcg agcaggccga ccgtatcggc atagcgcagg cgggcggcgg
7921 cgcattgctg cgcgacctcg gcgatagcgc gcagggtctg gccgctggcc cgcgaggcgt
7981 cctcgcagcc gatgcatacc ttcaggccga gggtatgcgc aagatggata aacatcgcca
8041 gccgctccag cagcaccgcc agcggctcgc gcagtttgta ctgccgcagc ttatccgaag
8101 ccggaatcga gatatccacc cagtcgatgc ccagatcggc gctctggcgg atctccagcg
8161 cgttcatccg acaccaggtc atcagggtcg cgtcgggcag ctggcgacgc accagctgga
8221 tccgcgcgat ctcctcgtcg cccatcgccg gggtgccgac ctccatcgcc gttattcctg
8281 cggcgtaaag cgcctcggca atggcgacct tttcgctggt gcgaaaggcg acgccggggc
8341 tctgctcgcc gtcgcgcagg gtggtatcgt taatcagcac gcgttccatg gtgtggaatg
8401 cacgacgacc cccaccacga gtgtgaaagg cacggcgacc gccaccacgc gcctcctcgc
8461 cggccagccg ccgatactgg gcaaagcggg cccgcgcgtc ctcctcggtt cggctaaaga
8521 gcgcatccgc cagatgcggc gtcgttttgt gcagcgaggc gtagcgcact tcgccaagca
8581 aaaagtcgcg gaagctctcc tccggctctt cggaatcgag cataaacggc gtcttacctt
8641 ccgcttcccg ctgcggatga tagcgccaca ggtgccagta tcccgcctca accgcccgtt
8701 tcgcctcgcg ctggctgcag cgcataccgg ctttcagccc gtggttaatg caggcggcgt
8761 aggcaatcac cagcgacggt cccggccagg cttcggcctc ggcgatcgcc cgtagggtct
8821 gatctttatc agcgcccatc gcgacctggg ccacgtacac attgccgtag ctcatcgcca
8881 tcatgccgag atcttttttc cgcgtgcgtt tgccctgcgc ggcaaacttc gcgatggccg
8941 ccaccggggt cgatttagac gactggccgc cggtattgga gtaaacctcg gtgtcaaaca
9001 ccagaatatt gacgtcttcc ccgctcgcca gcacgtgatc gagaccgccg aagccgatat
9061 cgtaggccca gccgtcgccg ccgaaaatcc actgcgaacg acgaacaaaa tagtcgcggt
9121 tctgccacag ctgctccaac agcggcacgc cctctttttc cgccgccagc cgttcgctga
9181 gccggtccgc gcgctcgcgg gtgccctcgc cttcatcctg cttcgccagc cactggcgca
9241 ttgcgtcgct aagttcgtcg ctgaccggta gcgccagcgc ggcggtcata tcatcggcga
9301 tttgttgacg caccgcctgg ccgccgagca tcatgccgag gccaaactcc gcattatcct

9361 caaacagcga gttcgcccat gccgggccat ggccgcggtg gttggtggta tagggaatcg
9421 acggcgcgct ggctccccag atagaagagc agccggtggc gttagcgatc agcatccggt
9481 cgccaaacag ctgggttatc aggcgggcat aaggcgtttc accgcatccc gcgcaggcgc
9541 cggaaaactc cagcagcggg gtttcaaact ggctgccttt gaccgtcgtc ttacgaaacg
9601 gattgctctt cggcgtcagc gccagcgcat agtcccagac cggcgccatc tgacgctggc
9661 tatcgagaga ctgcattttt aacgccttgc cgcgcgcggg acagatatcc acgcagttgc
9721 cgcagccgga acaatccagc ggcgagatag ccagatggta gtgatactcc ttcgctccct
9781 gcgcgggttt gctcagcagc ccaaccggcg cggcgtcatg ctcttcgccg ttgagcagcg
9841 ccgggcggat cgccgcatgc gggcagataa aggcgcactg gttacactgc gtgcagccct
9901 ccggctgcca gaccggcact tccagcgcga tcccgcgttt ctcccacgcg gcggtgcccg
9961 aaggaaaggt cccgtcctcc ataccgacga acgcgctcac cggcagctgg tcgccgcact
10021 ggcggttcat cggctgcaga atatcgcgga tgaaatccgg catcatggct gatgcttgcg
10081 ccgcgggttc atccagcgtc gcccagtgcg ccggaatcgt cacctgatgc agcgaggcca
10141 tgcccagctc gatcgcccgc tggttcatct caatcaccgc cgcccctttg ctgccgtagc
10201 tttttcaac cgcctgcttg aggtaatccg ccgcggtctg cgggtcgata atcgccgcca
10261 gcttaaagaa cgccgcctgc atcagcatat taaagcgccc gcccagcccg agctcgcggg
10321 cgatatccac ggcgttcagg gtataaaaat ggatattttc ccgcgccaga tagcgtttaa
10381 agccgaccgg cagatgctgc tccagctccg catcggacca gctgcagttg agtaaaaagg
10441 tcccgcccgg ctttaatccg tccagcagat cgtagcgctc aacgtaggac tgctgcgaac
10501 aggagataaa atcggcccga tggatcaggt agggcgaatt gatcggccgg tcgccgaagc
10561 gtaaatgtga aacggtaatg ccgccggatt ttttcgagtc ataagaaaag taggcctgcg
10621 cgtagagcgg cgttttatcg ccgataattt tgatcgcgct tttattggcc ccgacggtgc
10681 cgtccgagcc catgccccaa aatttacagg cggtgatgcc gtcatgcgag accgccagcg
10741 tctgctggcg cggcggtaac gaagtaaagg ttacatcatc gacaatcccg agggtaaacc
10801 cgtccatcgg cagcggttta ttgaggttat caaagacggc cgcgatatcg ttgggcagaa
10861 catccttccc gccaagcgca tagcggccgc cgacgattag cggcgcatcg tcgtggtggt
10921 agaaggcgtt tttcacatcc aggcacagcg gttcagcctg agcgccgggc tctttggtac
10981 ggtcaaggac ggcaatccgc tgcacggttt tcggcagctg ggcgaagaag tgggccagcg
11041 aaaaagggcg aaacagatgc acgctgagca gcccgacctt ctctcccgcc gcgttcagcg
11101 tatccaccac ttcctgaacg gtatcgcaga ccgatcccat tgcgataatc acccgttcgg
11161 catccgccgc gccggtatag ttaaacagat gatactcccg gccggtgagc gcgctgattt
11221 gcgtcatata gctttcgaca atgtcgggca gcgcctgata aaaacggttg cccgcctccc
11281 gctcctggaa gtagatatcc gggttctgcg ccgttccgcg gatgaccgga tgatccggat
11341 gcagcgcgtt acggcggaag ctgtcgagcg cgggccggtc cagcagcgtc gccagctgct
11401 catattccaa cacctcgatt ttttgaattt cgtgcgaggt gcgaaaaccg tcgaagaagt
11461 taacaaacgg gatgcgtccc ttaatcgccg ccagatgcgc caccgccgac aaatccatca
11521 cctgctgcac gttgttctcc gccagcatcg cgcagccggt ctggcggacc gccatcacat
11581 cctggtgatc gccaaaaata ttcagcgaat tggtcgccag cgcccgggcg ctgacgtgaa
11641 agacgcccgg cagcagttca ccggcgattt tgtacatgtt ggggatcatc agcagcagcc
11701 cctgggaggc cgtataggtg gtggtgagcg ccccggcctg cagcgcgccg tggaccgcgc
11761 ctgccgcgcc ggcctccgac tgcatctcca ttaagcgcac cggctggcca aaaaggttct
11821 ttttcccctg cgccgcccac tcgtcgacgt tttccgccat cggcgtggag gggttatgg
11881 ggtaaatcgc cgcgacctcg gtaaaggcat aagagatcca ggccgccgcg gcgttgccat
11941 ccattgtttt catttttccg gacattgttc aatcctcgaa ggtgagaggc atcttcgccg
12001 cctcaaataa gcggcaaacc cagttgttgc ctcaagcaca gcctgtgcca gctcgcggat

12061 gacagaagag ttagcgcgaa ttcaacgcgt tatgaagaga gtcgccgcgc agcgcgccaa
12121 gagattgcgt ggaataagac acagggggcg acaagctgtt gaacaggcga caaagcgcca
12181 ccatggcccc ggcaggcgca attgttctgt ttcccacatt tggtcgcctt attgtgccgt
12241 tttgttttac gtcctgcgcg gcgacaaata actaacttca taaaaatcat aagaatacat
12301 aaacaggcac ggcagtacta ctagagctag cactagactt aagtgttgcg ctcctgtcgg
12361 aaaagggggt tgaaaatacg cgttctcgca ggggtattgc gaaggctgtg ccaggttgct
12421 ttgcactacc gcggcccatc cctgccccaa aacgatcgct tcagccctct cccgccgcgc
12481 gcggcggggc tggcggggcg cttaaaatgc aaaaagcgcc tgcttttccc ctaccggatc
12541 aatgtttctg cacatcacgc cgataagggc gcacggtttg catggttatc accgttcgga
12601 aaacaccgcg gcgtccctgt cacggtgtcg gacaaattgt cataactgcg acacaggagt
12661 ttgcgatgac cctgaatatg atgctcgata acgccgtacc cgaggcgatt gccggtgcgc
12721 tgactcaaca acatccgggg ctgtttttta caatggtcga acaggcatcg gtagcgattt
12781 ccctcaccga tgcccgggcg aatattacct acgccaaccc ggcgttttgc cgccagactg
12841 gatactcgct ggcgcaattg ctcaatcaaa acccgcgcct gctggccagc agccagacgc
12901 cgcgcgagat ctaccaggag atgtggcaaa ccctgctcca gcgccagccg tggcgcggtc
12961 agctaattaa tcaggcccgc gacggcggcc tgtatctggt agatatcgat atcacgccgg
13021 tgctgaatcc gcagggcgag ctggagcatt atctggcgat gcagcgggat atcagcgtca
13081 gctataccct ggaacagcgg ctgcgcaatc atatgacgct aatggaagcg gtgctcaata
13141 acatccccgc cgccgtggtc gtggtcgatg agcaggatcg ggtggtgatg gataatctcg
13201 cctacaaaac gttctgcgcg gactgcggcg ggaaagagct gctggtcgag ctccaggttt
13261 ccccgcgcaa aatggggccc ggcgcggagc aaatcctgcc ggtggtggtt cgcggcgcgg
13321 tccgctggct gtcggtaacc tgctgggcgc tgcccggcgt gagtgaagaa gccagccgct
13381 acttcgtcga cagcgccccg gcgcgcacgc tgatggtgat cgccgactgt acccagcagc
13441 gccagcagca ggagcagggc cggctcgacc gtctgaaaca gcaaatgacc gccggtaagc
13501 tgctggccgc gattcgcgag tcgctggacg cggcgctgat tcagcttaat tgcccaatca
13561 atatgctggc ggcggcccgc cggctgaacg gcgaaggcag cggcaacgtg gcgctggacg
13621 cggcgtggcg cgaaggtgaa gaggccatgg cgcgcctgca gcgctgccgc ccttctcttg
13681 agctggaaag caatgccgtc tggccgcttc agcccttttt tgacgacctg tacgccctct
13741 accgcacccg ctttgacgat cgcgcgcggc tgcaggtgga catggcatcg ccgcatctgg
13801 tcggcttcgg ccagcgtacc cagctgctgg cctgcttgag tttatggctc gaccggacgc
13861 tggccctcgc cgccgagctg ccctccgtac cgctggagat cgagctttac gccgaagagg
13921 acgagggctg gctctctttg tatctcaacg acaatgtccc gctgctgcag gtgcgctacg
13981 cccactcccc cgatgcccta aactctcccg gcaaagggat ggagctgcgg ctgatccaaa
14041 cgctggtcgc ctaccaccgc ggcgcgattg aactggcttc gcgaccgcag ggaggcacca
14101 gcctggttct gcgtttcccg ctctttaata ccctgaccgg aggtgagcaa tgatccataa
14161 atccgattcg gacaccaccg tcagacgttt cgatctctcc cagcagttta ccgccatgca
14221 gcggataagc gtggtcctga gtcgcgccac cgaagcgagc aaaaccctgc aggaggttct
14281 gagcgtgcta cataacgatg cctttatgca gcacgggatg atttgcctgt acgacagcca
14341 gcaggagatc ctgagcatcg aagcgctgca gcaaacggaa gatcagacgc tgcccggcag
14401 tacgcaaatt cgctaccggc cgggggaagg attagtcggt accgtgctgg cgcagggcca
14461 gtcgctggtg ctgccgcgcg tcgccgacga ccagcgtttt ctcgatcgtc tgagcctgta
14521 cgactatgac ctgccgttta tcgccgttcc gctgatgggc ccccactccc ggcccatcgg
14581 cgtactggcg gcgcacgcga tggcgcgtca ggaagagcgg ctgcccgcct gcacgcgctt
14641 tctcgaaacc gtcgccaatc tgatcgccca gacgattcgc ctgatgatcc tgccaacctc
14701 cgccgcgcag gcgccgcagc agagccccag aatagagcgc ccgcgcgcct gtaccccttc

14761 gcgcggtttc ggcctggaaa atatggtcgg taaaagcccg gcgatgcggc agattatgga
14821 tattattcgt caggtttccc gctgggatac cacggtgctg gtacgcggcg agagcggcac
14881 cgggaaagag ctcatcgcca acgccatcca ccataattct ccgcgcgccg ccgcggcgtt
14941 cgtcaaattt aactgcgcgg cgctgccgga caacctgctg gagagcgagc tgtttggtca
15001 tgagaaaggc gcgtttaccg gcgcggtgcg ccagcggaaa ggccgctttg agctggcgga
15061 cggcggcacc ttattcctcg atgagatcgg cgaaagcagc gcctcgtttc aggctaagct
15121 actgcgtatt ctgcaagagg gggagatgga gcgcgtcggc ggcgacgaaa ccctgcgggt
15181 caacgtgcgc attatcgcgg cgaccaaccg ccatctggaa gaggaggtgc ggctgggtca
15241 tttccgcgag gatctatact accgcctgaa cgtaatgcct atcgcgctgc cgccgctgcg
15301 cgagcgccag gaggatatcg ccgagctggc gcactttctg gtgcgaaaaa tcgcccacag
15361 ccaggggcga acgctgcgca tcagcgatgg ggcgattcgc ctgctgatgg agtacagctg
15421 gccgggaaac gtgcgcgaac tggaaaactg tctcgaacgt tcggcggtgc tgtcggaaag
15481 cggcctgata gaccgggacg tgattctgtt caaccatcgc gataacccgc cgaaagcgct
15541 cgccagcagc ggcccggcgg aggacggctg gctcgataac agcctcgacg agcgccagcg
15601 gctgatcgcc gccctggaaa aagcgggctg ggtgcaggcc aaagcggcgc ggctgctcgg
15661 catgaccccg cgccaggtgg cgtatcgcat tcagattatg gatatcacca tgccgcgact
15721 gtcttaagac tagtggatcc cccgggctgc aggaattcga tggacctagt aagcttgctt
15781 cttgatgcgc ctaacccccct cgctgccagc ctttcatcaa caaatagcca tcccagcgcg
15841 ataggtcata aagcatcaca tgccgccatc ccttgtccga ttgttggctt tgtcgcaaag
15901 ccaacaacct cttttcttta aaaatcaagg ctccgcttct ggagcgcgaa ttgcatcttc
15961 cccctcatcc cccaccgtca acgaggtcac tatgaaggga aatgaaattc tggcgctgct
16021 ggatgaaccg gcctgtgaac acaaccataa acaaaaatcc ggctgcagcg cgcccaaacc
16081 cggcgccacc gccggcggct gcgcgttcga cggcgcgcag ataaccctgc tgcccatcgc
16141 cgacgtggcg catctggtcc acggccccat cggctgcgcc ggaagctcat gggataaccg
16201 cggcagcgcc agctccggcc ccacccttaa tcggctcggg ttcaccaccg atctcaacga
16261 acaggacgtg attatgggcc gcggcgaacg ccgcttgttt cacgccgtgc gccatatcgt
16321 cacccgctat catccggcgg cggtctttat ctacaacacc tgcgtaccgg ccatggaggg
16381 cgatgacctg gaagcggtat gccaggccgc gcagaccgcc accggcgtac cggttatcgc
16441 tattgacgcc gccggtttct acggcagtaa aaatctcggt aaccggctgg cgggcgacgt
16501 catggtcaaa cgggtcatcg gccagcgcga gcccgccccc tggccggaga gcacgctctt
16561 tgccccggag cagcgtcacg atattggcct gattggcgaa ttcaatattg ccggcgagtt
16621 ctggcatatt cagccgctgc tcgacgaact ggggatccgc gtgctcggca gcctctccgg
16681 tgatggccgc ttcgccgaga tccagaccat gcaccgggcg caggccaata tgctggtctg
16741 ctcgcgggcg ttaattaacg tcgccagagc cctggagcag cgctacggca cgccgtggtt
16801 cgaaggcagc ttttacggga tccgcgccac ctctgacgcc ctgcgccagc tggcggcgct
16861 gctgggcgac gacgaccttc gccagcgcac cgaagcgctg attgcgcggg aggaacaggc
16921 ggcggaactg gcgctacagc cgtggcgcga acagctgcgc ggccgcaaag cgctgctcta
16981 taccggcggg gtgaaatcct ggtcggtggt atcggcgctg caggatttgg gcatgaccgt
17041 ggtggcaacc ggcacgcgta aatccaccga agaggataaa cagcggatcc gcgagctgat
17101 gggcgaagag gcggtaatgc tggaagaggg caacgcccgc acgctgctgg atgtggtcta
17161 tcgctatcag gccgacctga tgattgccgg cggacgcaat atgtacaccg cctataaagc
17221 caggctgccg tttctcgata tcaatcagga gcgcgaacac gccttcgctg gctatcaggg
17281 gatcgtcacc ctcgcccgcc agctgtgtca gaccatcaac agccccatct ggccgcaaac
17341 ccattctcgc gccccgtggc gcgcacgtgg tggaggtcgt cgtgcattct ctactcgtgg
17401 tggcggtcgt cgcgccttct ctactatggc agacattttc cgcaccgata agccgctggc

17461 ggtcagcccc atcaaaaccg gccagccgct cggcgcaatc ctcgccagcc tcgggatcga
17521 acacagcatc cctctggtcc acggcgcgca ggggtgcagc gccttcgcca aagtcttttt
17581 tattcaacat ttccacgacc cggttcccct gcagtcgacg gcgatggacc ccacgtcgac
17641 gattatgggc gcggacggca atatttttac cgccctggat accctctgcc agcgcaacaa
17701 tccgcaggct atcgtactgc tcagcaccgg gctgtcggag gcccagggca gcgatatttc
17761 ccgcgtggtt cgccagtttc gcgaagagta tccccggcat aagggggtgg cgatattgac
17821 ggttaacacg ccggattttt atggctccat ggagaacggc ttcagcgcgg tgttagagag
17881 cgtcattgag cagtgggtgc cgccggcgcc gcgcccggct cagcgcaatc gccgggtcaa
17941 tctgctggtc agccatctct gttcgccggg cgatatcgag tggctgcgcc gatgcgtcga
18001 agcctttggt ctgcagccga taatcctgcc ggacctggcg caatcgatgg acggccacct
18061 ggcgcagggc gatttctcgc cgctgaccca gggcgggacg ccgctgcgcc agatagagca
18121 gatggggcaa agcctgtgca gcttcgccat tggcgtctcc cttcatcgcg cctcatcgct
18181 gctggccccg cgctgccgcg gcgaggttat cgccctgccg cacctgatga ccctcgaacg
18241 ctgcgacgcc tttattcatc aactggcgaa aatttccgga cgcgccgttc ccgagtggct
18301 ggaacgccag cgcggccagc tacaggatgc gatgatcgac tgccatatgt ggctccaggg
18361 ccagcgcatg gcgatagcgg cggaaggcga tttgctggcg gcgtggtgtg atttcgccaa
18421 cagccagggg atgcagcccg gcccgctggt ggcccctacc ggtcatccca gcctgcgcca
18481 gctgccggtg gaacgggtgg tgccggggga tctggaggat ctgcaaaccc tgctgtgcgc
18541 gcatcccgcc gacctgctgg tggcgaactc gcacgcccgc gacctggcgg agcagtttgc
18601 gctgccgctg gtgcgcgcgg gttttccgct ctttgacaag ctcggcgaat tccgccgggt
18661 gcgacagggg tatagcggga tgcgcgatac gctgtttgag ctggcaaacc tgatacgcga
18721 gcgtcaccac cacctcgccc actaccgatc gccgctgcgc cagaaccccg aatcgtcact
18781 ctccacgggt ggcgcgtacg cagcggacgg tggaggtggt tctggaggtg gaggttccgg
18841 aggtggaggt tctacttcct gctcctcttt ttctggcggc aaagcctgcc gcccggcgga
18901 tgacagcgca ttgacgccgc ttgtggccga taaagctgcc gcgcacccct gctactctcg
18961 ccatgggcat caccgtttcg cgcggatgca tctgcccgtc gcgcccgcct gcaatttgca
19021 gtgcaactac tgtaatcgca aattcgattg cagcaacgag tcccgccccg ggtatcgtc
19081 aacgctgctg acgcctgaac aggcggtcgt gaaagtgcgt caggtcgcgc aggcgatccc
19141 gcagctttcg gtggtgggca tcgccgggcc cggcgatccg ctcgccaata tcgcccgcac
19201 ctttcgcacc ctggagctga tccgcgaaca gctgccggac ctgaaattat gtctgtcgac
19261 caacggactg atgctgcctg acgcggtgga ccgcctgctg gatgtcggcg ttgaccacgt
19321 cacggtcacc attaacaccc tcgacgcgga gattgccgcg caaatctacg cctggctatg
19381 gctggacggc gaacgctaca gcgggcgcga agcgggagag atcctgattg cccgtcagct
19441 tgagggcgta cgcaggctga ccgccaaagg cgtgctggtg aaaataaatt cggtgctgat
19501 ccccggtatc aacgatagcg gcatggccga cgtgagccgc gcgctgcggg ccagcggcgc
19561 gtttatccat aatattatgc cgctgatcgc caggccggag cacggcacgg tgtttggcct
19621 caacggccag ccggagccgg acgccgagac gctcgccgcc acccgcagcc ggtgcggcga
19681 agtgatgccg cagatgaccc actgccacca gtgtcgcgcc gacgccattg ggatgctcgg
19741 cgaggaccgc agccagcagt ttacccagct tccggcgcca gagagtctcc cggcctggct
19801 gccgatcctc caccagcgcg cgcagctgca cgccagcatt gcgacccgcg gcgaatctga
19861 agccgatgac gcctgcctgg tcgccgtggc gtcaagccgc ggggacgtca ttgattgtca
19921 ctttggtcac gccgaccggt tctacattta cagcctctcg gccgccggta tggtgctggt
19981 caacgagcgc tttacgccca aatattgtca ggggcgcgat gactgcgagc cgcaggataa
20041 cgcagcccgg tttgcggcga tcctcgaact gctggcggac gttaaagccg tattctgcgt
20101 gcgtatcggc catacgccgt ggcaacagct ggaacaggaa ggcattgaac cctgcgttga

20161 cggcgcgtgg cggccggtgt ccgaagtgct gcccgcgtgg tggcaacagc gtcgggggag
20221 ctggcctgcc gcgttgccgc ataagggggt cgcctaaggc cttatgtgag attcaggaca
20281 ttgtcgccag cgcggcggaa ttgcgacaat tcagggacgc gggttgccaa gctttctagt
20341 tctagttacg tacagcgcgc caagagattg cgtggaataa gacacagggg gcgacaagct
20401 gttgaacagg cgacaaagcg ccaccatggc cccggcaggc gcaattgttc tgtttcccac
20461 atttggtcgc cttattgtgc cgttttgttt tacgtcctgc gcggcgacaa ataactaact
20521 tcataaaaat cataagaata cataaacagg cacggctggt atgttccctg cacttctctg
20581 ctggcaaaca ctcaacaaca ggagaagtca ccatgaccat gcgtcaatgc gctatttacg
20641 gtaaaggcgg tatcggtaaa tccaccacca cgcagaacct cgtcgccgcg ctggcggaga
20701 tgggtaagaa agtgatgatc gtcggctgcg atccgaaggc ggactccacc cgtctgattc
20761 tgcacgccaa agcacagaac accattatgg agatggccgc ggaagtcggc tcggtcgagg
20821 acctcgaact cgaggacgtg ctgcaaattg gctacggcga tgtgcgctgc gcggaatccg
20881 gcggcccgga gccaggcgtc ggctgcgcgg gacgcggcgt gatcacggcg atcaactttc
20941 ttgaagaaga aggcgcctac gaggacgatc tcgatttcgt gttctatgac gtgctcggcg
21001 acgtggtctg cggcggcttc gccatgccga tccgcgaaaa caaagcccag gagatctaca
21061 tcgtctgctc cggcgaaatg atggcgatgt acgcggccaa caatatctcc aaagggatcg
21121 ttaaatacgc caaatccggc aaggtgcgcc tcggcggcct gatctgtaac tcacgtcaga
21181 ccgaccgtga agatgaactg attattgccc tggcggaaaa gctcggtacc cagatgatcc
21241 actttgtgcc ccgcgacaac atcgtgcagc gcgcggagat ccgccgcatg acggttatcg
21301 agtacgaccc cgcctgtaaa caggccaacg aataccgcac cctggcacag aagatcgtca
21361 acaacaccat gaaagtggtg ccgacgccct gcaccatgga tgagctggaa tcgctgctga
21421 tggagttcgg catcatggaa gaggaggaca ccagcatcat tggcaaaacc gccgccgaag
21481 aaaacgcggc ccgtggcggt ggtcgtcgtg catttagcac tcgtggtgga ggtcgtcgtg
21541 ccttctctac tatgatgacc aacgcaacgg gcgaacgtaa tctggcgctg atccaggaag
21601 tcctggaggt gttcccggaa accgcgcgaa aagagcgcag aaagcacatg atggtcagcg
21661 atccggaaat ggagagcgtc ggcaagtgca ttatctctaa ccgcaaatca caacccggcg
21721 taatgaccgt acgcggctgc gcctacgccg gttccaaagg ggtggtattt gggccgatta
21781 aggatatggc ccatatttcg cacggaccgg tcggctgcgg ccagtattcc cgcgccggac
21841 gacgaaacca gtacaccgga gtcagcggcg tcgatagctt cggcacgctg aacttcacct
21901 ctgattttca ggagcgcgac atcgtgttcg gcggcgataa aaagctcagc aagctgattg
21961 aagagatgga gttgctgttc ccgctcacca aagggatcac cattcagtcg gaatgcccgg
22021 tggggctgat cggtgatgat atcagcgcgg tggccaacgc cagcagcaag gcgctggata
22081 aaccggtgat cccggtacgc tgcgaaggct ttcgcggcgt gtcgcagtct ctggggcacc
22141 atatcgccaa cgacgtggtg cgcgactgga tcctgaacaa tcgcgaagga cagccgtttg
22201 aaaccacccc ttacgatgtg gcgatcatcg gcgactacaa catcggcggc gacgcctggg
22261 cctcgcgcat tctgctggaa gagatggggc tacgggtagt cgcgcagtgg tccggcgacg
22321 gcacgctggt ggagatggag aataccccat tcgtcaagct gaacctggtt cactgctacc
22381 gttcgatgaa ctatatcgcc cgccatatgg aggagaaaca tcagattccg tggatggagt
22441 acaacttctt cgggccgacc aaaatcgccg aatcgctgcg caaaatcgcc gaccagttcg
22501 acgataccat tcgcgcgaac gccgaagcgg tgatcgcccg gtatgagggg cagatggcgg
22561 cgattatcgc caaatatcgc ccgcgcctgg aggggcgtaa ggtgctgctc tatatgggcg
22621 gcctgcggcc gcgccacgtt attggcgcct atgaggatct cgggatggag atcatcgccg
22681 ccggctacga gtttgcccat aacgatgatt acgaccgcac cctgccggat ctgaaagagg
22741 gcacgctgct gttcgatgac gccagcagct acgagctgga agcgttcgtc aaggcgctga
22801 agcccgacct tatcggctcc ggcatcaagg aaaaatatat cttccagaaa atgggcgtgc

22861 cgttccgcca gatgcactcg tgggactatt ccggcccgta ccacggctac gatggtttcg
22921 ccattttcgc ccgcgatatg gatatgaccc tgaacaaccc ggcgtggaac gaactgaccg
22981 ctccgtggct gaagtctgcg cgtggcggag gtcgtcgtgc attctcaacc cgtggtggtg
23041 gtcgtcgcgc tttcagtaca atgagccaaa cgattgataa aattaatagc tgttatccgc
23101 tattcgaaca ggatgaatac caggagctgt tccgcaataa gcggcagctg gaagaggcgc
23161 acgatgcgca gcgcgtgcag gaggtctttg cctggaccac caccgccgag tatgaagcgc
23221 tgaatttcca gcgcgaggcg ctgaccgttg acccggcgaa agcctgccag ccgcttggcg
23281 cggtgctttg ctcgctggga tttgccaaca ccctgccgta tgtgcacggc tctcaggggt
23341 gcgtggccta ctttcgcacc tattttaacc gccatttcaa agagccgatc gcctgcgtct
23401 ccgactcgat gaccgaagat gcggcggtgt tcggcggcaa caacaatatg aacctgggcc
23461 tgcagaacgc cagcgcgctg tacaaaccgg agatcattgc ggtgtccacc acctgcatgg
23521 cggaagttat cggcgatgac ctgcaggcgt ttatcgccaa cgctaaaaaa gatggcttcg
23581 tcgacagcag catcgccgtg ccccacgccc atacgcctag ctttatcggc agccacgtca
23641 ccggctggga taacatgttt gaaggcttcg ccaaaacctt cactgcggac taccaggggc
23701 agccgggcaa attgccgaag ctcaatctgg tgaccggctt tgaaacctat ctcggcaact
23761 tccgcgtatt aaagcggatg atggaacaga tggcggtgcc gtgcagcctg ctctccgatc
23821 cgtcggaagt tctcgacacg cccgccgacg gccactatcg gatgtattcc ggcggcacca
23881 cgcagcagga gatgaaagag gcccctgacg ccatcgatac gctgctcctg cagccgtggc
23941 agctgctgaa gagcaaaaaa gtggtgcagg agatgtggaa ccagcccgcc accgaggtcg
24001 ccattccgct ggggctggcc gccaccgatg aactgctgat gaccgtcagc cagcttagcg
24061 gcaagccgat tgccgacgcc ctcacccttg agcgcggccg gctggttgac atgatgctcg
24121 actcccacac ctggctgcac ggcaagaagt ttggcctgta cggcgatccg gacttcgtga
24181 tgggcctcac ccgcttcctg ctggagctgg gctgcgagcc aacggtgatc ctgagccata
24241 acgccaacaa acgctggcaa aaagcgatga acaaaatgct cgatgcctcg ccgtacgggc
24301 gcgatagcga agtgtttatc aactgcgatt tgtggcactt ccgttcgctg atgttcaccc
24361 gtcagccgga ctttatgatc ggcaactcct acggcaagtt tatccagcgc gataccctgg
24421 cgaagggtaa agcctttgaa gtgccgctta tccgcctcgg ctttccgctg ttcgaccgcc
24481 accatctgca ccgccagaca acctggggtt atgaaggggc gatgaacatt gtgacgacgc
24541 tggtgaacgc cgtgctggag aaactggata gcgataccag ccagctgggc aaaaccgatt
24601 acagcttcga tctcgtccgt taatccctgt tttgtgcttg ttgcccgctg accccgcggg
24661 ctttttttcg cgtatggacg ctcttcccca cgttacgctc aggggaatat tccgttcacg
24721 gttgttccgg gcttcttgat gcgcctaacc ccctcgctgc cagcctttca tcaacaaata
24781 gccatcccag cgcgataggt catatacgta tctagtgttt aaactctaga gaagacattg
24841 tcttgattgc tcccaaagta tataaggtgt ttaaaccagg agcaggagtg gcatggggat
24901 ccggacccgc gccgctagcc gtgtcgtttc tgtgacaaag cccacaaaac atcgcgacac
24961 tgtaggacga accttgtcag gactaataca caaccatttg aaaaatatta attttattct
25021 ctggtatcgc aattgctagt tcgttatcgc caccgcgctt ccgcggtgaa ccgcgccccg
25081 gcgttttccg tcaacatccc tggagctgac agcatgtgga attactccga gaaagtaaaa
25141 gaccattttt ttaacccccg caatgcgcgc gtggtggaca acgccaacgc ggtaggcgac
25201 gtcggttcgt taagctgcgg cgacgccctg cgcctgatgc tgcgcgtcga cccgcaaagc
25261 gaaatcattg aggaggcggg cttccagacc ttcggctgcg gcagcgccat cgcctcctcc
25321 tccgcgctga cggagctgat tatcggccat accctcgccg aagccgggca gataaccaat
25381 cagcagattg ccgattatct cgacggactg ccgccggaga aaatgcactg ctcggtgatg
25441 ggccaggagg ccctgcgcgc ggccatcgcc aactttcgcg gcgaaagcct tgaagaggag
25501 cacgacgagg gcaagctgat ctgcaaatgc ttcggcgtcg atgaagggca tattcgccgc

25561 gcggtacaga acaacgggct gaccacccctt gccgaggtga tcaactacac caaagcgggc
25621 ggcggctgca cctcttgcca cgaaaaaatc gagctggccc tggcggagat cctcgcccag
25681 cagccgcaga cgacgccagc cgtggccagc ggcaaagatc cgcactggca gagcgtcgtc
25741 gataccatcg cagaactgcg gccgcatatt caggccgacg gcggcgatat ggcgctactc
25801 agcgtcacca accaccaggt gaccgtcagc ctctccggca gctgtagcgg ctgcatgatg
25861 accgatatga ccctggcctg gctgcagcaa aaactgatgg aacgtaccgg ctgttatatg
25921 gaagtggtgg cggcccgcgg tggaggtcgc cgtgcatttt cgacccgtgg tggaggccgc
25981 cgtgcattca gcaccatgaa acaggtttat ctcgataaca acgccaccac ccgtctggac
26041 ccgatggtcc tggaagcgat gatgcccttt ttgaccgatt tttacggcaa cccctcgtcg
26101 atacacgatt ttggcattcc ggcccaggcg gctctggaac gcgcgcatca gcaggctgcg
26161 gcgctgctgg gcgcggagta tcccagcgag atcatcttta cctcctgcgc caccgaagcc
26221 accgccaccg ccatcgcctc ggcgatcgcc ctgctgcctg agcgtcgcga aatcatcacc
26281 agcgtggtcg aacatccggc gacgctggcg gcctgcgagc acctggagcg ccagggctac
26341 cggattcatc gcatcgcggt ggatagcgag gggcgctgg acatggcgca gttccgcgcg
26401 gcgctcagcc cgcgcgtcgc gttggtcagc gtgatgtggg cgaataacga aaccggggtg
26461 cttttcccga tcggcgaaat ggcggagctg gcccatgaac aaggggcgct gtttcactgc
26521 gatgcggtgc aggtggtcgg gaaaataccg atcgccgtgg gccagacccg catcgatatg
26581 ctctcctgct cggcgcataa gttccacggg ccaaaaggcg taggctgtct ttatctgcgg
26641 cggggaacgc gctttcgccc gctgctgcgc ggcggtcacc aggagtacgg tcggcgagcc
26701 gggacagaaa atatctgcgg aatcgtcggc atgggcgcgg cctgcgagct ggcgaatatt
26761 catctgccgg gaatgacgca tatcggccaa ttgcgcaaca ggctggagca tcgcctgctg
26821 gccagcgtgc cgtcggtcat ggtgatgggc ggcggccagc cgcgggtgcc cggcacggtg
26881 aatctggcct ttgagtttat tgaaggtgaa gccattctgc tgctgttaaa ccaggccggg
26941 atcgccgcct ccagcggcag cgcctgcacc tcaggctcgc tggaaccctc ccacgtgatg
27001 cgggcgatga atatccccta caccgccgcc cacggcacca tccgcttttc tctctcgcgc
27061 tacacccggg agaaagagat cgattacgtc gtcgccacgc tgccgccgat tatcgaccgg
27121 ctgcgcgcgc tgtcgcccta ctggcagaac ggcaagccgc gcccggcgga cgccgtattc
27181 acgccggttt acggctaatc ccgtcagccg ggactgtgcg ggtagcctcg gcggctaccc
27241 gttaacgcct acagcacggt ggtttaaacg ctttatatag attgggacga aaaaacgct

//

Complete sequences of construct pBDS3942

```
     source          1..28582
                     /organism="synthetic DNA construct"
                     /mol_type="other DNA"
     misc_feature    118..744
                     /note="5'-YNRC-Delta-9 (Chr XIV)"
     promoter        1017..1620
                     /note="ScGAL1 promoter"
     CDS             1622..1828
                     /codon_start=1
                     /note="su9.1"

/translation="MASTRVLASRLASQMAASAKVARPAVRVAQVSKRTIQTGSPLQTL
                     KRTQMTSIVNATTRQAFQKRAYSS"
     CDS             1829..1900
                     /codon_start=1
                     /product="peptide that binds Strep-Tactin(R), an engineered
form of streptavidin"
                     /note="Strep-Tag II"
                     /translation="MGSSWSHPQFEKGGGSGGGSGGSA"
     CDS             1901..1933
                     /codon_start=1
                     /product="peptide that binds Strep-Tactin(R), an engineered
form of streptavidin"
                     /note="Strep-Tag II"
                     /translation="WSHPQFEKSSG"
     CDS             1934..2812
                     /codon_start=1
                     /note="Sc_KoNifH"

/translation="MTMRQCAIYGKGGIGKSTTTQNLVAALAEMGKKVMIVGCDPKADS
TRLILHAKAQNTIMEMAAEVGSVEDLELEDVLQIGYGDVRCAESGGPEPGVGCAGRGVI
TAINFLEEEGAYEDDLDFVFYDVLGDVVCGGFAMPIRENKAQEIYIVCSGEMMAMYAAN
NISKGIVKYAKSGKVRLGGLICNSRQTDREDELIIALAEKLGTQMIHFVPRDNIVQRAE
IRRMTVIEYDPACKQANEYRTLAQKIVNNTMKVVPTPCTMDELESLLMEFGIMEEEDTS
IIGKTAAEENAA"
     CDS             2813..2872
                     /codon_start=1
                     /note="S10S_HD"
                     /translation="RGGGRRAFSTRGGGRRAFST"
     CDS             2873..4321
                     /codon_start=1
                     /note="Sc_KoNifD"
```

/translation="MMTNATGERNLALIQEVLEVFPETARKERRKHMMVSDPKMKSVGK
CIISNRKSQPGVMTVRGCAYAGSKGVVFGPIKDMAHISHGPAGCGQYSRAERRNQYTGV
SGVDSFGTLNFTSDFQERDIVFGGDKKLSKLIEEMELLFPLTKGITIQSECPVGLIGDD
ISAVANASSKALDKPVIPVRCEGFRGVSQSLGHHIANDVVRDWILNNREGQPFETTPYD
VAIIGDYNIGGDAWASRILLEEMGLRVVAQWSGDGTLVEMENTPFVKLNLVHCYRSMNY
IARHMEEKHQIPWMEYNFFGPTKIAESLRKIADQFDDTIRANAEAVIARYEGQMAAIIA
KYRPRLEGRKVLLYIGGLRPRHVIGAYEDLGMEIIAAGYEFAHNDDYDRTLPDLKEGTL
LFDDASSYELEAFVKALKPDLIGSGIKEKYIFQKMGVPFRQMHSWDYSGPYHGYDGFAI
FARDMDMTLNNPAWNELTAPWLKSA"

CDS 3170..3172
/note="Y100Q"

CDS 4322..4381
/codon_start=1
/note="S10S_DK"
/translation="RGGGRRAFSTRGGGRRAFST"

CDS 4382..5944
/codon_start=1
/note="Sc_KoNifK"

/translation="MSQTIDKINSCYPLFEQDEYQELFRNKRQLEEAHDAQRVQEVFAW
TTTAEYEALNFRREALTVDPAKACQPLGAVLCSLGFANTLPYVHGSQGCVAYFRTYFNR
HFKEPIACVSDSMTEDAAVFGGNNNMNLGLQNASALYKPEIIAVSTTCMAEVIGDDLQA
FIANAKKDGFVDSSIAVPHAHTPSFIGSHVTGWDNMFEGFAKTFTADYQGQPGKLPKLN
LVTGFETYLGNFRVLKRMMEQMAVPCSLLSDPSEVLDTPADGHYRMYSGGTTQQEMKEA
PDAIDTLLLQPWQLLKSKKVVQEMWNQPATEVAIPLGLAATDELLMTVSQLSGKPIADA
LTLERGRLVDMMLDSHTWLHGKKFGLYGDPDFVMGLTRFLLELGCEPTVILSHNANKRW
QKAMNKMLDASPYGRDSEVFINCDLWHFRSLMFTRQPDFMIGNSYGKFIQRDTLAKGKA
FEVPLIRLGFPLFDRHHLHRQTTWGYEGAMNIVTTLVNAVLEKLDSDTSQLGKTDYSFD
LVR"

terminator 5946..6188
/note="ScVMA161 ter"

promoter 6257..6835
/note="ScPGK1 promoter"

sig_peptide 6836..7042
/note="su9.3"

CDS 7043..7861
/codon_start=1
/note="Sc_KonifU"

/translation="WNYSEKVKDHFFNPRNARVVDNANAVGDVGSLSCGDALRLMLRVD
PQSEIIEEAGFQTFGCGSAIASSSALTELIIGHTLAEAGQITNQQIADYLDGLPPEKMH
CSVMGQEALRAAIANFRGESLEEEHDEGKLICKCFGVDEGHIRRAVQNNGLTTLAEVIN
YTKAGGGCTSCHEKIELALAEILAQQPQTTPAVASGKDPHWQSVVDTIAELRPHIQADG
GDMALLSVTNHQVTVSLSGSCSGCMMTDMTLAWLQQKLMERTGCYMEVVAA"

CDS 7862..7921
/codon_start=1
/note="S10S_US"
/translation="RGGGRRAFSTRGGGRRAFST"
CDS 7922..9124
/codon_start=1
/note="Sc_KonifS"

/translation="MKQVYLDNNATTRLDPMVLEAMMPFLTDFYGNPSSIHDFGIPAQA
ALERAHQQAAALLGAEYPSEIIFTSCATEATATAIASAIALLPERREIITSVVEHPATL
AACEHMEREGYRIHRIAVDGEGALDMAQFRAALSPRVALVSVMWANNETGVLFPIGEMA
ELAHEQGALFHCDAVQVVGKIPIAVGQTRIDMLSCSAHKFHGPKGVGCLYLRRGTRFRP
LLRGGHQEYGRRAGTENICGIVGMGAACELANIHLPGMTHIGQLRNRLEHRLLASVPSV
MVMGGGQPAVPGTVNLAFEFIEGEAILLLLNQAGIAASSGSACTSGSLEPSHVMRAMNI
PYTAAHGTIRFSLSRYTREKEIDYVVATLPPIIDRLRALSPYWQNGKPRPADAVFTPVY
G"
terminator 9126..9226
/note="T1-ScADH1 ter"
promoter 9295..10159
/note="ScGAL7.M5 promoter"
sig_peptide 10160..10366
/note="su9.5"
CDS 10367..10891
/codon_start=1
/note="Sc_KonifF"

/translation="ANIGIFFGTDTGKTRKIAKMIHKQLGELADAPVNINRTTLDDFMA
YPVLLLGTPTLGDGQLPGLEAGCESESWSEFISGLDDASLKGKTVALFGLGDQRGYPDN
FVSGMRPLFDALSARGAQMIGSWPNEGYEFSASSALEGDRFVGLVLDQDNQFDQTEARL
ASWLEEIKRTVL"
CDS 10892..10954
/codon_start=1
/note="S10_FM"
/translation="RGGGRRAFHTRGGGRRAFHT"
CDS 10955..11752
/codon_start=1
/note="Sc_KoNifM"

/translation="MNPWQRFARQRLARSRWNRDPAALDPADTPAFEQAWQRQCHMEQT
IVARVPEGDIPAALLENIAASLAIWLDEGDFAPPERAAIVRHHARLELAFADIARQAPQ
PDLSTVQAWYLRHQTQFMRPEQRLTRHLLLTVDNDREAVHQRILGLYRQINASRDAFAP
LAQRHSHCPSALEEGRLGWISRGLLYPQLETALFSLAENALSLPIASELGWHLLWCEAI
RPAAPMEPQQALESARDYLWQQSQQRHQRQWLEQMISRQPGLCG"
CDS 11753..11815
/codon_start=1

/note="S10_MY"
/translation="RGGGRRAFHTRGGGRRAFHT"
CDS 11816..12475
/codon_start=1
/note="Sc_KoNifY"

/translation="MSDNDTLFWRMLALFQSLPDLQPAQIVDWLAQESGETLTPERLAT
LTQPQLAASFPSATAVMSPARWSRVMASLQGALPAHLRIVRPAQRTPQLLAAFCSQDGL
VINGHFGQGRLFFIYAFDEQGGWLYDLRRYPSAPHQQEANEVRARLIEDCQLLFCQEIG
GPAAARPIRHRIHPMKAQPGTTIQAQCEAINTLLAGRLPPWLAKRLNRDNPLEERVF"
CDS 12476..12508
/codon_start=1
/product="Myc (human c-Myc oncogene) epitope tag"
/note="Myc"
/translation="EQKLISEEDL"
terminator 12510..12748
/note="ScYHI9 ter"
promoter 12791..13196
/gene="S. cerevisiae LEU2"
/note="LEU2 promoter"
CDS 13197..14291
/codon_start=1
/gene="S. cerevisiae LEU2"
/product="3-isopropylmalate dehydrogenase, required for leucine biosynthesis"
/note="LEU2"
/note="yeast auxotrophic marker"

/translation="MSAPKKIVVLPGDHVGQEITAEAIKVLKAISDVRSNVKFDFENHL
IGGAAIEATGVPLPDEALEASKKADAVLLGAVGGPKWGTGSVRPEQGLLKIRKELQLYA
NLRPCNFASDSLLDLSPIKPQFAKGTDFVVVRELVGGIYFGKRKEDDGDGVAWDSEQYT
VPEVQRITRMAAFMALQHEPPLPIWSLDKANVLASSRLWRKTVEETIKNEFPTLKVQHQ
LIDSAAMILVKNPTHLNGIIITSNMFGDIISDEASVIPGSLGLLPSASLASLPDKNTAF
GLYEPCHGSAPDLPKNKVNPIATILSAAMMLKLSLNLPEEGKAIEDAVKKVLDAGIRTG
DLGGSNSTTEVGDAVAEEVKKILA"
misc_feature 14292..14392
/note="LEU2 DOWN"
promoter 14427..15125
/note="SpdGAL2.M14 Pro (S. paradoxus)"
sig_peptide 15127..15333
/note="su9.4"
CDS 15334..18843
/codon_start=1
/note="Sc_KonifJ"

/translation="SGKMKTMDGNAAAAWISYAFTEVAAIYPITPSTPMAENVDEWAAQ
GKKNLFGQPVRLMEMQSEAGAAGAVHGALQAGALTTTYTASQGLLLMIPNMYKIAGELL
PGVFHVSARALATNSLNIFGDHQDVMAVRQTGCAMLAENNVQQVMDLSAVAHLAAIKGR
IPFVNFFDGFRTSHEIQKIEVLEYEQLATLLDRPALDSFRRNALHPDHPVIRGTAQNPD
IYFQEREAGNRFYQALPDIVESYMTQISALTGREYHLFNYTGAADAERVIIAMGSVCDT
VQEVVDTLNAAGEKVGLLSVHLFRPFSLAHFFAQLPKTVQRIAVLDRTKEPGAQAEPLC
LDVKNAFYHHDDAPLIVGGRYALGGKDVLPNDIAAVFDNLNKPLPMDGFTLGIVDDVTF
TSLPPRQQTLAVSHDGITACKFWGMGSDGTVGANKSAIKIIGDKTPLYAQAYFSYDSKK
SGGITVSHLRFGDRPINSPYLIHRADFISCSQQSYVERYDLLDGLKPGGTFLLNCSWSD
AELEQHLPVGFKRYLARENIHFYTLNAVDIARELGLGGRFNMLMQAAFFKLAAIIDPQT
AADYLKQAVEKSYGSKGAAVIEMNQRAIELGMASLHQVTIPAHWATLDEPAAQASAMMP
DFIRDILQPMNRQCGDQLPVSAFVGMEDGTFPSGTAAWEKRGIALEVPVWQPEGCTQCN
QCAFICPHAAIRPALLNGEEHDAAPVGLLSKPAQGAKEYHYHLAISPLDCSGCGNCVDI
CPARGKALKMQSLDSQRQMAPVWDYALALTPKSNPFRKTTVKGSQFETPLLEFSGACAG
CGETPYARLITQLFGDRMLIANATGCSSIWGASAPSIPYTTNHRGHGPAWANSLFEDNA
EFGLGMMLGGQAVRQQIADDMTAALALPVSDELSDAMRQWLAKQDEGEGTRERADRLSE
RLAAEKEGVPLLEQLWQNRDYFVRRSQWIFGGDGWAYDIGFGGLDHVLASGEDVNILVF
DTEVYSNTGGQSSKSTPVAAIAKFAAQGKRTRKKDLGMMAMSYGNVYVAQVAMGADKDQ
TLRAIAEAEAWPGPSLVIAYAACINHGLKAGMRCSQREAKRAVEAGYWHLWRYHPQREA
EGKTPFMLDSEEPEESFRDFLLGEVRYASLHKTTPHLADALFSRTEEDARARFAQYRRL
AGEE"

CDS 18844..18906
/codon_start=1
/note="S10_JV"
/translation="ARGGGRRAFHTRGGGRRAFHT"

CDS 18907..20046
/codon_start=1
/note="Sc_KonifV"

/translation="MERVLINDTTLRDGEQSPGVAFRTSEKVAIAEALYAAGITAMEVG
TPAMGDEEIARIQLVRRQLPDATLMTWCRMNALEIRQSADLGIDWVDISIPASDKLRQY
KLREPLAVLLERLAMFIHLAHTLGLKVCIGCEDASRASGQTLRAIAEVAQQCAAARLRY
ADTVGLLDPFTTAAQISALRDVWSGEIEMHAHNDLGMATANTLAAVSAGATSVNTTVLG
LGERAGNAALETVALGLERCLGVETGVHFSALPASCQRVAEAAQRAIDPQQPLVGELVF
THESGVHVAALLRHSESYQSIAPSLMGRSYRLVLGKHSGRQAVNGVFDQMGYHLNAAQI
NQLLPAIRRFAENWKRSPKDYELVAIYDELCGESALRARG"

CDS 20047..20109
/codon_start=1
/note="S10_VW"
/translation="RGGGRRAFHTRGGGRRAFHT"

CDS 20110..20370
/codon_start=1
/note="Sc_KonifW"

/translation="MMEWFYQIPGVDELRSAESFFQFFAVPYQPELLGRCSLPVLATFH

RKLRAEVPLQNRLEDNDRAPWLLARRLLAESYQQQFQESGT"
     terminator   20372..20589
                     /note="ScCYC1 ter"
     misc_feature   20622..20782
                     /note="gap9 sequence"
     promoter   20813..21523
                     /note="SaGAL1.M1 Pro (S. arboricola)"
     sig_peptide   21525..21731
                     /note="su9.2"
     CDS   21732..23102
                     /codon_start=1
                     /product="CAI：0.80"
                     /note="Sc_KoNifE"

/translation="MKGNEILALLDEPACEHNHKQKSGCSAPKPGATAAGCAFDGAQIT
LLPIADVAHLVHGPIGCAGSSWDNRGSASSGPTLNRLGFTTDLNEQDVIMGRGERRLFH
AVRHIVTRYHPAAVFIYNTCVPAMEGDDLEAVCQAAQTATGVPVIAIDAAGFYGSKNLG
NRPAGDVMVKRVIGQREPAPWPESTLFAPEQRHDIGLIGEFNIAGEFWHIQPLLDELGI
RVLGSLSGDGRFAEIQTMHRAQANMLVCSRALINVARALEQRYGTPWFEGSFYGIRATS
DALRQLAALLGDDDLRQRTEALIAREEQAAELALQPWREQLRGRKALLYTGGVKSWSVV
SALQDLGMTVVATGTRKSTEEDKQRIRELMGEEAVMLEEGNARTLLDVVYRYQADLMIA
GGRNMYTAYKARLPFLDINQEREHAFAGYQGIVTLARQLCQTINSPIWPQTHSRAPWR"
     CDS   23103..23165
                     /codon_start=1
                     /note="S10S_EN"
                     /translation="RGGGRRAFSTRGGGRRAFST"
     CDS   23166..24548
                     /codon_start=1
                     /product="CAI：0.79 "
                     /note="Sc_KoNifN"

/translation="MADIFRTDKPLAVSPIKTGQPLGAILASLGIEHSIPLVHGAQGCS
AFAKVFFIQHFHDPVPLQSTAMDPTSTIMGADGNIFTALDTLCQRNNPQAIVLLSTGLS
EAQGSDISRVVRQFREEYPRHKGVAILTVNTPDFYGSMENGFSAVLESVIEQWVPPAPR
PAQRNRRVNLLVSHLCSPGDIEWLRRCVEAFGLQPIILPDLAQSMDGHLAQGDFSPLTQ
GGTPLRQIEQMGQSLCSFAIGVSLHRASSLLAPRCRGEVIALPHLMTLERCDAFIHQLA
KISGRAVPEWLERQRGQLQDAMIDCHMWLQGQRMAIAAEGDLLAAWCDFANSQGMQPGP
LVAPTGHPSLRQLPVERVVPGDLEDLQTLLCAHPADLLVANSHARDLAEQFALPLVRAG
FPLFDKLGEFRRVRQGYSGMRDTLFELANLIRERHHHLAHYRSPLRQNPESSLSTGGAY
AAD"
     CDS   24549..24593
                     /codon_start=1
                     /note="GS linker"
                     /translation="GGGGSGGGGSGGGGS"
     CDS   24594..25994

```
                     /codon_start=1
                     /note="Sc_KonifB"

/translation="TSCLSFSGGKACRPADDSALTPLVADKAAAHPCYSRHGHHRFARM
HLPVAPACNLQCNYCNRKFDCSNESRPGVSSTLLTPEQAVVKVRQVAQAIPQLSVVGIA
GPGDPLANIARTFRTLELIREQLPDLKLCLSTNGLVLPDAVDRLLDVGVDHVTVTINTL
DAEIAAQIYAWLWLDGERYSGREAGEILIARQLEGVRRLTAKGVLVKINSVLIPGINDS
GMAGVSRALRASGAFIHNIMPLIARPEHGTVFGLNGQPEPDAETLAATRSRCGEVMPQM
THCHQCRADAIGMLGEDRSQQFTQLPAPESLPAWLPILHQRAQLHASIATRGESEADDA
CLVAVASSRGDVIDCHFGHADRFYIYSLSAAGMVLVNERFTPKYCQGRDDCEPQDNAAR
FAAILELLADVKAVFCVRIGHTPWQQLEQEGIEPCVDGAWRPVSEVLPAWWQQRRGSWP
AALPHKGVA"
     terminator      25999..26134
                     /note="ScNAT1 ter"
     misc_feature    26200..26872
                     /note="3'-YNRC-Delta-9 (Chr XIV)"
     CDS             27000..27815
                     /codon_start=1
                     /product="aminoglycoside phosphotransferase"
                     /note="KanR"
                     /note="confers resistance to kanamycin in bacteria or G418
(Geneticin(R)) in eukaryotes"

/translation="MSHIQRETSCSRPRLNSNMDADLYGYKWARDNVGQSGATIYRLYG
KPDAPELFLKHGKGSVANDVTDEMVRLNWLTEFMPLPTIKHFIRTPDDAWLLTTAIPGK
TAFQVLEEYPDSGENIVDALAVFLRRLHSIPVCNCPFNSDRVFRLAQAQSRMNNGLVDA
SDFDDERNGWPVEQVWKEMHKLLPFSPDSVVTHGDFSLDNLIFDEGKLIGCIDVGRVGI
ADRYQDLAILWNCLGEFSPSLQKRLFQKYGIDNPDMNKLQFHLMLDEFF"
     rep_origin      27908..28527
                     /label=pBR322_origin
                     /note="pBR322_origin"
ORIGIN
        1 cgttatcccc tgattctgtg gataaccgta ttaccgcctt tgagtgagct gataccgctc
       61 gccgcagccg aacgaccgag cgcagcgagt cagtgagcga ggaagcggaa gtctagattc
      121 gtgaaacacg tgggataccg gttaggtaat attacagttt ccttacacac ttactaaata
      181 cgcaacagag catgttatta atcgtatatt taagtaataa taacgtattg accattccta
      241 agactgaagc agtaccaaga aatggcactt tttgtttata tgacatagta aaagtgttca
      301 tttggcagaa aacaagggta tctggatact gcaatcctca ttagttgcgg agagcttttt
      361 taaagtttta ttacttcgat aatgctttcc tccagaactg ggcgcgcact gaagccataa
      421 actgagtggt tgtccctgtg tatattctac aatcgcctat ttgaaacttc tgggtgataa
      481 tatcgattta gcgattgggt ttctcccttc tgagattctg gataaatgag atgctaccct
      541 cacagtaaaa acagtacaaa acctttgtat agttctagtc aaatatgaac tccttcattc
      601 atggattgta tactattgtt cttgttgaaa gattcctctt gtgattttca tggtgatttt
      661 aagccttttt tattgaaaga attaaatatt cactaggctg cgatacgata gacaaacgaa
      721 gtgattgaaa cccgaattaa cggagaagac gcaggtatcg cgtgattgag gagcataaca
```

781 tgtttagtga aggtttcttt tggaaaactt cagtcgctca ttattagaac cagggaggtc
841 caggctttgc tggtgggaga gaaagcttat gaagctgggg ttgcagattt gtcgattggt
901 cgccagtaca cagttttaaa aagtcagaga atgtagagaa gtatggatct ttgaaaccta
961 tatattttgg gaagaataaa gctttttgtt gatttcaaac tatataaggt gggcccatat
1021 tacatggcat taccaccata tacatatcca tatctaatct tacttatatg ttgtggaaat
1081 gtaaagagcc ccattatctt agcctaaaaa aaccttctct ttggaacttt cagtaatacg
1141 cttaactgct cattgctata ttgaagtacg gattagaagc cgccgagcgg gcgacagccc
1201 tccgacggaa cactctcctc cgtgcgtcct cgtcttgacc ggtcgcgttc ctgaaacgca
1261 gatgtgcctc gcgccgcact gctccgaaca ataaagattc tacaatacta gcttttatgg
1321 ttatgaagag gaaaaattgg cagtaacctg gccccacaaa ccttcaaatt aacgaatcaa
1381 attaacaacc ataggatgat aatgcgatta gtttttagc cttatttctg ggtaattaa
1441 tcagcgaagc gatgattttt gatctattaa cagatatata aatggaaaag ctgcataacc
1501 actttaacta atactttcaa cattttcagt ttgtattact tcttattcaa atgtcataaa
1561 agtatcaaca aaaaattgtt aatatacctc tatactttaa cgtcaaggag aaaaaactat
1621 aatggcatct actagagttt tggcttcaag attagcttct cagatggctg cttccgctaa
1681 ggtagcaaga ccagctgttc gtgttgctca agtttctaag agaacaattc aaacaggatc
1741 tcctttacaa acattaaaaa gaactcaaat gacttctata gttaatgcta ctacaagaca
1801 agcttttcaa aaaagggctt attcttctat gggcagtagc tggtcacatc cgcagtttga
1861 aaaaggtggt ggatctggcg gcggttctgg tggttcagcc tggagccatc cgcagtttga
1921 aaaaagcagc ggcatgacta tgagacaatg tgctatttat ggtaaaggtg gaattggaaa
1981 atctactaca acacagaatt tagtggctgc ccttgccgaa atgggtaaaa aggttatgat
2041 tgtcggatgt gaccctaaag ctgacagtac taggctgata ctacacgcaa aagcacaaaa
2101 taccattatg gagatggctg cagaagttgg ttcagtcgag gacttggaat tggaggacgt
2161 tttgcagatt ggttatggag acgtaagatg tgctgaaagt ggtggtccag aaccaggtgt
2221 tggttgcgca ggtagaggtg ttataactgc aattaatttt ttggaggaag aaggtgcata
2281 cgaggacgat ttggacttcg tgttttacga cgtccttggc gatgtggttt gcggaggttt
2341 cgcaatgccc attagagaaa ataaggctca agaaatctat atcgtttgtt caggagaaat
2401 gatggcaatg tatgctgcaa ataacatatc aaagggcatc gttaagtatg caaaaagtgg
2461 taaggttaga ctaggaggcc taatttgtaa ctcaagacaa acagacagag aagatgaatt
2521 aatcattgca ttggcagaaa aattgggtac tcaaatgatc cacttcgtcc caagagacaa
2581 tattgtccaa agggctgaaa tcagaagaat gaccgttatc gaatatgatc cagcatgcaa
2641 gcaagctaac gaatatagaa ctctagcaca gaagattgtt aataatacta tgaaggtagt
2701 gcctactcca tgtactatgg acgaattaga gtctttacta atggaatttg gtatcatgga
2761 agaagaagat acctctataa taggcaagac cgctgcagag gaaaatgctg ccagaggcgg
2821 tggtagaagg gcttttagca ctagaggtgg aggacgtaga gcgttctcta ctatgatgac
2881 caatgctact ggcgaaagaa acctggcatt gattcaagag gttttggagg tatttccaga
2941 gactgcaaga aaggaaagaa ggaaacacat gatggtttcc gatccaaaga tgaaaagtgt
3001 aggtaagtgc attatttcta atagaaagtc tcaacctggc gttatgacgg taagaggttg
3061 tgcttatgcc ggctctaaag gtgttgtttt tggaccaatt aaggacatgg ctcatatatc
3121 tcacggccct gctggctgcg gtcaatattc cagggccgag agaagaaacc aatatacagg
3181 cgtctcaggt gtagattctt tcggtactct aaatttcact tcagatttcc aagaaagaga
3241 tatagttttt ggaggtgata aaaagttgtc taaactgatc gaagaaatgg aattgttatt
3301 ccctttgaca aagggaataa ccatacaatc cgaatgtcct gtaggtttga tcggcgatga
3361 tatatccgct gtcgcaaacg cttcttctaa ggctttggac aagcctgtca tcccagtcag
3421 atgcgagggt tttagaggcg tatctcaatc tttgggtcat catatcgcaa atgacgttgt

3481 cagagactgg atattgaata atagagaagg ccaaccattc gaaacaactc cttatgatgt
3541 tgctataatt ggtgattaca acattggcgg agacgcatgg gcatccagaa tacttttaga
3601 agaaatgggt ctgagagtag ttgcacaatg gagcggtgat ggtactttgg ttgaaatgga
3661 aaacacacca tttgtaaaac taaacttagt tcattgttat agatctatga actatatagc
3721 taggcacatg gaagagaaac accagatccc ttggatggaa tataattttt ttggaccaac
3781 caagatagca gaatctctga gaaaaattgc tgatcaattt gatgatacga ttagagcaaa
3841 cgcagaggct gttatagcta gatatgaggg tcagatggcc gccataatcg ccaaatacag
3901 gcccaggtta gaaggcagaa aagtcttgtt atatattgga ggtttgaggc caaggcatgt
3961 cattggagct tacgaagatt tgggtatgga aataatagct gcaggttacg agttcgctca
4021 taacgatgat tatgacagga ctttgccaga tctgaaagaa ggtactttgt tgtttgatga
4081 cgcatctagt tacgagttag aagccttcgt aaaagcctta aaaccagatt taattggctc
4141 tggcatcaaa gaaaagtaca tttttcaaaa aatgggtgtc ccattcagac agatgcattc
4201 atgggattat tccggtcctt atcatggtta tgatggattc gctatttttg caagagatat
4261 ggacatgaca ttaaataatc cagcttggaa tgaactaact gcaccttggt tgaagagcgc
4321 tcgtggcggt ggtagaaggg ccttttctac cagaggtggc ggacgtaggg catttagcac
4381 catgtctcag actatcgaca aaataaattc ttgttatcca ttattcgaac aagatgaata
4441 ccaagagcta tttagaaata aaaggcaatt ggaagaagcc catgatgctc aaagagtgca
4501 agaagtgttt gcttggacta caacagcaga atatgaagcc ttaaatttca gaagagaggc
4561 tttaactgtt gaccctgcta aggcttgcca accattgggc gcagtcttgt gctctttagg
4621 ctttgcaaac accctgcctt atgtccacgg ttcacagggt tgcgtcgctt actttagaac
4681 gtacttcaat agacatttca aggaacctat tgcatgtgta tcagacagta tgactgaaga
4741 tgctgcagtg tttggcggta acaataatat gaatttaggt ctacaaaacg cctctgctct
4801 ttataagcct gaaattattg cagtgtccac tacgtgcatg gctgaagtca ttggagacga
4861 cctgcaagcc tttattgcca atgcaaagaa agatggtttt gtcgattcct caatagctgt
4921 gcctcatgcc catactccat catttattgg ctctcatgtg actggttggg acaatatgtt
4981 tgaaggtttt gcaaaaacct ttactgccga ttaccaaggt caaccaggca agttaccaaa
5041 actaaacttg gtaactggat tcgagactta tttaggtaat ttcagagttt taaagagaat
5101 gatggagcaa atggctgtac catgtagcct tttaagtgat ccatcagagg tcttggatac
5161 accagcagac ggtcattata gaatgtactc aggtggaacc actcagcagg aaatgaagga
5221 agctcctgac gctatcgata cattactatt gcagccttgg cagttattga aatcaaaaaa
5281 ggttgttcaa gaaatgtgga atcaaccagc tacagaagtt gctatcccac ttggtttagc
5341 cgcaactgac gagctactga tgacagtctc ccaactttca ggtaaaccta tagccgacgc
5401 tctaactctg gagagaggca ggttggtgga catgatgcta gactcccata catggttgca
5461 tggaaaaaag tttggtttat atggtgatcc tgattttgtc atgggtttaa ctagatttct
5521 gttagaattg ggttgcgagc caaccgttat attgtctcac aatgccaata aaagatggca
5581 gaaagctatg aataagatgc ttgatgcaag cccatacggt agagattctg aggtttttat
5641 taattgtgat ctgtggcatt ttagatctct gatgtttact agacagccag attttatgat
5701 aggtaactca tatggtaaat tcatacaaag ggatactttg gcaaaaggta aggcctttga
5761 ggtcccatta atcagactag gttttcctct atttgataga catcaccttc atagacaaac
5821 aacctggggt tacgaaggtg ctatgaacat agtcaccaca ttggttaacg ccgtcttaga
5881 aaaattagat agtgacacat cccaattggg aaaaacggac tatagttttg atttagtaag
5941 ataagcgctc aaaccaggct tttcttttcc gtttttacga gctagataag cgcatccata
6001 tttactaata gatataatga gatatctgag atacatgtgt atgtatatat gcacgttttc
6061 ttttattatc taaaaatcat attatattaa gtaagagaaa aaaatgtaca actatataaa
6121 tatatattta tttaaaatgg ttttgaattt ttcctattct ggttgatatt gcccaaaagc

6181 tattcagtgg taccgcttta tataatttga aacgaatcaa ctgatttttt agtttcaaat
6241 tatataaggt gggccctccc tccttcttga attgatgtta ccctcataaa gcacgtggcc
6301 tcttatcgag aaagaaatta ccgtcgctcg tgatttgttt gcaaaaagaa caaaactgaa
6361 aaaacccaga cacgctcgac ttcctgtgtt cctattgatt gcagcttcca atttcgtcac
6421 acaacaaggt cctagcgacg gctcacaggt tttgtaacaa gcaatcgaag gttctggaat
6481 ggcgggaaag ggtttagtac cacatgctat gatgcccact gtgatctcca gagcaaagtt
6541 cgttcgatcg tactgttact ctctctcttt caaacagaat tgtccgaatc gtgtgacaac
6601 aacagcctgt tctcacacac tcttttcttc taaccaaggg ggtggtttag tttagtagaa
6661 cctcgtgaaa cttacattta catatatata aacttgcata aattggtcaa tgcaagaaat
6721 acatatttgg tcttttctaa ttcgtagttt ttcaagttct tagatgcttt ctttttctct
6781 tttttacaga tcatcaagga agtaattatc tactttttac aacaaatata aaacaatggc
6841 ctccactaga gtcctcgcct ctcggctggc ctcccagatg gccgcttccg ccaaggtagc
6901 ccgccctgct gtccgcgttg cccaggtcag caagcgcacc atccaaactg gctccccccct
6961 ccagaccctc aagcgtaccc agatgacctc catcgtcaac gccaccactc gccaggcttt
7021 ccagaagcgc gcctactctt cctggaatta ctctgaaaag gtaaaagacc atttttttcaa
7081 tcccaggaac gctagagtag tagataatgc caacgcagtt ggtgatgtag gttcattgtc
7141 atgtggcgat gctttgagat taatgttgag agtcgatcca caatctgaaa taatagaaga
7201 agctggtttc caaacatttg gttgtggctc agctattgct tcttcctccg ctttgacaga
7261 attaattatc ggacacacac tagccgaagc cggacagata acaaatcaac agattgctga
7321 ttacttagat ggattgccac ctgaaaaaat gcattgttct gtaatgggac aagaagccct
7381 aagagctgcc atcgctaatt tcagaggcga atcattagaa gaagagcatg acgaaggcaa
7441 actaatttgc aaatgctttg gagtggatga aggacacatc agaagagccg tacaaaacaa
7501 tggtttaaca accttggcag aagttatcaa ctacaccaaa gccggcggag gctgtacaag
7561 ttgtcatgag aaaatcgaac tagccttggc tgagatattg gcccaacagc ctcaaactac
7621 accagcagtt gcctctggaa aagatcctca ttggcagtca gttgtggata ccatcgcaga
7681 gctgcgccca catattcaag ctgatggagg tgatatggct ttgctatctg ttactaacca
7741 tcaagttact gtatcactgt ctggttcatg cagcggctgt atgatgacag atatgacgct
7801 ggcctggtta caacaaaagt tgatggaaag aacgggttgt tacatggagg tggttgcagc
7861 cagagggggt ggtagaagag ctttctcaac tagaggtggt ggaagaagag cgttttcgac
7921 tatgaaacag gtttacctag acaacaatgc aacaacaagg ttggacccta tggttcttga
7981 agccatgatg ccatttttga cagactttta tggtaaccca tcttctatac acgattttgg
8041 tattcctgcc caggccgccc tggaaagagc ccatcaacag gctgctgcat tgctaggcgc
8101 agaataccca agtgaaataa tcttcacttc ttgcgctact gaagctacag caactgctat
8161 cgcctcagcc attgccttgt tacctgaaag aagagaaatc atcacttcgg tcgtggaaca
8221 ccctgctaca ttagccgcat gcgaacacat ggagagagag ggttacagaa tacacagaat
8281 tgcagttgat ggtgaaggag ctttagacat ggctcagttc agagcagctc ttagtccaag
8341 agttgcctta gtaagtgtaa tgtgggcaaa taacgaaacc ggtgtgttat ttcctattgg
8401 agaaatggca gaattagccc acgagcaagg tgctttgttc cattgtgacg ctgttcaagt
8461 ggtaggtaaa attccaattg ccgtgggtca gacaagaatt gatatgttga gctgttctgc
8521 tcacaaattc cacggaccaa aaggagttgg ttgcctatac ttaagaagag gtacaagatt
8581 cagacctcta ctgagaggtg gtcatcaaga atacggtaga agagctggaa ctgaaaacat
8641 ttgcggtatt gttggtatgg gagcagcttg tgaacttgct aatatccact tgccaggtat
8701 gacacacatt ggccaattga gaaacaggtt agaacacaga ttgctggcaa gcgtaccatc
8761 tgtaatggta atgggtggtg gccaacctgc agtcccagga acagtcaatt tagcattcga
8821 atttattgag ggcgaagcaa tcttgttgtt acttaatcaa gctggtattg ccgcaagtag

8881 tggctctgcc tgtacttctg gctctttaga accctctcat gttatgagag ctatgaacat
8941 tccttataca gctgcccacg gcactatcag attctctttg tccagataca ccagggagaa
9001 agaaattgac tacgttgtcg ctaccctacc tccaataatt gatagactga gagcactgtc
9061 gccttactgg cagaatggta aaccaaggcc tgctgatgct gtatttacac ctgtctatgg
9121 ttaagcgaat ttcttatgat ttatgatttt tattattaaa taagttataa aaaaaataag
9181 tgtatacaaa ttttaaagtg actcttaggt tttaaaacga aaattcggta ccgctttata
9241 tatattggga aaaacaaata cgtttgttcg tcccaagcta tataaggtgg tacccctggt
9301 agatacattg atggtctcaa ggtggtaccc ctggtagata cattgatgct atcaatcaag
9361 agaactggaa agattgtgta accttgaaaa acggtgaaac ttacgggtcc aagattgtct
9421 acagattttc ctgatttgcc agcttactat ccttcttgaa aatatgcact ctatatcttt
9481 tagttcttaa ttgcaacaca tagatttgct gtacaacgaa ttttatgcta ttttctaaat
9541 ttggagttca gtgataaaag tgtcacagcg aatttcctca catgtaggga ccgaattgtt
9601 tacaagttct ctgtaccacc ttggagacat caaaaattga aaatctatgg aaagatatgg
9661 acggaagcaa caagaatata gcacgagccg cggagttcat ttcgttactt ttgatatcac
9721 tcacaactat tgcgaagcgc tccagtgaaa aaatcataag gaaaagttgt aaatattatt
9781 cgtagtattc gtttggtaaa gtagaggggg aaatttttcc cctttatttt gttcatacat
9841 tcttaaattg ctttgcctct ccttttggaa agctatactt cggagcactg ttgagcgaag
9901 gctcattaga tatattttct gtcattttcc ttaacccaaa aataagggaa agggtccaaa
9961 aagcgctcgg acaactattg accgtgaccc gtaggactgg ctatacagtg ttcacaaaat
10021 agccaagctg aaaatagtgt gtagctatgt tcagttagtt tggctagcaa agatataata
10081 gcaggtcgga aatatttatg gccgttatta tgcagaccat caacatgata aaaaaaaaca
10141 gttgaatatt ccctcaaaaa tggcctcgac tcgtgtcctc gcctctcgcc tggcctcgca
10201 gatggctgct tccgccaagg ttgctcgccc tgctgtccgc gttgctcagg tcagcaaacg
10261 caccatccag actggctccc ccctccagac tctcaagcgc acccagatga cctccatcgt
10321 gaacgccacc acccgccagg ctttccagaa gcgtgcctac tcttccgcaa acatcggtat
10381 tttctttggt actgatacag gtaaaactag aaagatcgct aagatgatcc ataagcaatt
10441 gggtgaattg gctgatgcac cagttaacat caacagaact acattagatg atttcatggc
10501 atacccagtt ttgttgttgg gtactccaac attgggtgac ggtcaattac caggtttgga
10561 agctggttgt gaatctgaat catggtctga gtttatttca ggtttagatg atgcatcttt
10621 gaagggtaaa acagttgctt tgtttggttt gggtgaccaa agaggttatc cagataattt
10681 tgtttctggt atgaggccat tgtttgatgc tttgtcagca agaggtgctc aaatgattgg
10741 ttcttggcca aatgaaggtt acgaattttc agcatcttca gctttagaag gtgacagatt
10801 cgttggttta gttttggatc aagataacca attcgatcaa actgaagcaa gattagcttc
10861 atggttggaa gaaattaaaa gaacagtttt ggcgagaggt ggcggacgtc gtgcttttca
10921 tactagggga ggcggtagaa gagctttcca tacaatgaac ccttggcaga gatttgctag
10981 acaaaggttg gctcgttcga gatggaatcg tgacccagca gctttggacc cagcagatac
11041 accagcattc gagcaagcat ggcaacgtca gtgtcatatg gaacaaacga ttgttgctag
11101 ggttcctgaa ggagatatac ctgcagcatt gttggaaaat attgctgcat ctctagcaat
11161 ttggttagat gaaggagatt tcgctcctcc agagagagcc gccattgtaa gacatcatgc
11221 tagactagaa ctagctttcg ccgatattgc tagacaagct ccccagcctg atttgtctac
11281 agttcaagcc tggtatttga gacaccagac acagtttatg aggcctgaac agagactgac
11341 caggcacctt ttactgaccg ttgataatga tcgtgaagct gttcatcaga gaatccttgg
11401 tttgtataga cagataaacg caagtagaga tgcttttgct ccactagctc aaaggcactc
11461 ccactgccca tctgcactag aggaaggcag attgggatgg ataagcagag gacttttgta
11521 tcctcaactg gaaaccgcac tattctcatt ggctgaaaat gcacttagcc ttccaattgc

11581 tagcgaatta ggatggcatc ttttatggtg tgaagcaata agacccgctg ctcctatgga
11641 accacagcaa gcacttgagt cggctcggga ctatttatgg caacaaagtc aacaacgtca
11701 tcagagacag tggttagaac aaatgatttc acgtcaacca ggtctatgtg gtgcaagagg
11761 cggtggtagg agggcgtttc acactagagg gggcggtaga agggcttttc acaccatgtc
11821 agacaatgat acattgtttt ggagaatgtt ggctctgttt caatccttac ctgatttgca
11881 gccagctcaa attgttgact ggctagcaca ggaaagtggt gagacattga cgccagaaag
11941 attagctacc ttaacacagc cccagttagc tgcatcgttt ccttctgcca cagccgttat
12001 gtccccagcc agatggtcaa gagttatggc aagtctacaa ggtgctctgc ctgcccactt
12061 aagaatcgtg aggccagctc aaagaacacc acaattatta gccgcatttt gctctcaaga
12121 tggtttggtt attaacggtc attttggaca aggtagattg tttttattt atgcattcga
12181 cgaacagggc ggttggcttt acgatctaag aagataccca tctgctccac atcaacagga
12241 agcaaatgag gttagagcta gactaataga ggactgccaa cttttattct gtcaggaaat
12301 tggtggacct gcagcagcta gacccataag acacagaatc cacccaatga aggcacagcc
12361 aggtacaaca atccaagcac aatgtgaggc catcaacacc ttgcttgctg gtagacttcc
12421 accttggttg gctaaaagat taaacagaga caaccccttg gaagagagag tttttgaaca
12481 aaagctaatc tccgaggagg acttgtaaga ttctaaacgc atagttgtaa ggttgatgta
12541 tatatatata tatatgtata tattaattac aataatatgc tcccgcccaa atttttctcc
12601 ttcaataccg ccggaggcgg tattgaagga aatagacgga gaattcctta tcaagaaagc
12661 ttccatcaaa gtgtacataa gaagtgccga aattcgaagt attctttcag agagtatttt
12721 tgcaacatac caataagcca aattactcgg taccgcttta tatactttga aaaaaacaaa
12781 ttcctatata aactgtggga atactcaggt atcgtaagat gcaagagttc gaatctctta
12841 gcaaccatta tttttttcct caacataacg agaacacaca ggggcgctat cgcacagaat
12901 caaattcgat gactggaaat tttttgttaa tttcagaggt cgcctgacgc atatacctttt
12961 ttcaactgaa aaattgggag aaaaaggaaa ggtgagagcg ccggaaccgg cttttcatat
13021 agaatagaga agcgttcatg actaaatgct tgcatcacaa tacttgaagt tgacaatatt
13081 atttaaggac ctattgtttt ttccaatagg tggttagcaa tcgtcttact ttctaacttt
13141 tcttaccttt tacatttcag caatatatat atatatttca aggatatacc attctaatgt
13201 ctgcccctaa gaagatcgtc gttttgccag gtgaccacgt tggtcaagaa atcacagccg
13261 aagccattaa ggttcttaaa gctatttctg atgttcgttc caatgtcaag ttcgatttcg
13321 aaaatcattt aattggtggt gctgctatcg aagctacagg tgtcccactt ccagatgagg
13381 cgctggaagc ctccaagaag gctgatgccg ttttgttagg tgctgtgggt ggtcctaaat
13441 ggggtacagg tagtgttaga cctgaacaag gtttactaaa aatccgtaaa gaacttcaat
13501 tgtacgccaa cttaagacca tgtaactttg catccgactc tcttttagac ttatctccaa
13561 tcaagccaca atttgctaaa ggtactgact tcgttgttgt cagagaatta gtgggaggta
13621 tttactttgg taagagaaag gaagatgatg gtgatggtgt cgcttgggat agtgaacaat
13681 acaccgttcc agaagtgcaa agaatcacaa gaatggccgc tttcatggcc ctacaacatg
13741 agccaccatt gcctatttgg tccttggata aagctaatgt tttggcctct tcaagattat
13801 ggagaaaaac tgtggaggaa accatcaaga acgaattccc tacattgaag gttcaacatc
13861 aattgattga ttctgccgcc atgatcctag ttaagaaccc aacccaccta aatggtatta
13921 taatcaccag caacatgttt ggtgatatca tctccgatga agcctccgtt atcccaggtt
13981 ccttgggttt gttgccatct gcgtccttgg cctctttgcc agacaagaac accgcatttg
14041 gtttgtacga accatgccac ggttctgctc cagatttgcc aaagaataag gtcaacccta
14101 tcgccactat cttgtctgct gcaatgatgt tgaaattgtc attgaacttg cctgaagaag
14161 gtaaggccat tgaagatgca gttaaaaagg ttttggatgc aggtatcaga actggtgatt
14221 taggtggttc caacagtacc accgaagtcg gtgatgctgt cgccgaagaa gttaagaaaa

14281 tccttgctta aaaagattct cttttttttat gatatttgta cataaacttt ataaatgaaa
14341 ttcataatag aaacgacacg aaattacaaa atggaatatg ttcatagggt agtgaccgct
14401 ttgattattc ccaacatata taaggtattt aaggaggttt tacggaccag aggagctttc
14461 cagatccaga tcaccggaat atagggcaag agaacatcaa gcagcagccg cacccgagtt
14521 atcactggca actactttgc atcaaactcc aattatatgc agcagaatct tttcttttaa
14581 aacgcactta gcgtcaactc ggagggcttc cttccgggat gtcttaacca gattgcaatt
14641 tcttttccat tttacagtta aaaataggtc acgtgatcta aaatcgagaa agggttgcct
14701 catgaaggca ccggccgtca ctcatccgtg cggagaggtc tgcgccattc aggggtccat
14761 gtgccttaga gagtattgat gcagagggca tcgtcggggc ggaccactcc gaaccgacat
14821 tggttaagcc cttcccatct caagatagga acaaaattgc attatactcc tactagaaag
14881 ctatatgtgt acatgttctc atgaatatac atcgttctag gtagctattg ttgagaggag
14941 ggatactcag taggctaaaa tgtggggaaa ggatgagctt tgtagacaaa tataaacaat
15001 taacaattgg actaaaagtt tggtgttgag aatcgctctt cgctatacat cttgttcact
15061 tatcattgag attaaccaac attaagtaaa ataaagatta acataatata aaataatttt
15121 ttcataatgg cctccactcg tgtcctcgcc tctcgcctcg cctcccagat ggctgcttcc
15181 gccaaggtag cccgccctgc tgtccgcgtt gctcaggtga gcaagcgcac catccagact
15241 ggctcccctc tccagaccct caagcgcacc cagatgacat ccatcgtcaa cgccaccacc
15301 cgccaggcct tccagaagcg cgcctactct tcctctggta aaatgaaaac aatggatggt
15361 aatgctgctg ctgcttggat ttcttatgct tttactgaag ttgctgcaat ctatccaatt
15421 acaccatcta ctccaatggc tgaaaatgtt gatgaatggg ctgcacaagg taaaaagaat
15481 ttgtttggtc aaccagttag attgatggaa atgcaatcag aagctggtgc tgctggtgca
15541 gttcatggtg ctttacaagc tggtgcattg actacaactt atactgcatc tcaaggtttg
15601 ttattgatga tcccaaacat gtacaagatc gctggtgaat tgttgccagg tgtttttcat
15661 gtttcagcta gagcattggc tacaaattct ttgaacatct tcggtgacca tcaagatgtt
15721 atggctgtta gacaaactgg ttgtgcaatg ttggctgaaa acaacgttca acaagttatg
15781 gatttgtcag cagttgctca tttggctgca attaaaggta gaataccatt cgttaatttc
15841 tttgatggtt ttagaacatc tcatgaaatc caaaagattg aagttttaga atacgaacaa
15901 ttggcaactt tattggatag accagcttta gattctttta gaagaaacgc attgcatcca
15961 gatcatccag ttattagagg tacagctcaa aacccagata tctatttcca agaaagagaa
16021 gctggtaaca gattctacca agcattgcca gatatcgttg aatcatacat gacacaaatt
16081 tctgctttga ctggtagaga ataccatttg tttaattaca ctggtgctgc tgatgcagaa
16141 agagttatta tcgctatggg ttctgtttgt gatacagttc aagaagttgt tgatactttg
16201 aatgctgctg gtgaaaaggt tggtttattg tcagttcatt tgtttagacc atttctttg
16261 gcacatttct ttgctcaatt accaaaaaca gttcaaagaa ttgctgtttt ggatagaact
16321 aaagaaccag gtgcacaagc tgaaccattg tgtttggatg ttaaaaatgc attctaccat
16381 catgatgatg ctccattgat tgttggtggt agatacgctt taggtggtaa agatgttttg
16441 ccaaacgata tcgctgctgt ttttgataat ttgaataagc cattgccaat ggatggtttt
16501 acattgggta tcgttgatga tgttactttt acttctttgc caccaagaca acaaacattg
16561 gcagtttctc atgatggtat tactgcttgt aaattttggg gtatgggttc agatggtaca
16621 gttggtgcaa ataagtctgc tattaaaatc atcggtgaca agactccatt gtacgcacaa
16681 gcatatttct cttacgattc taagaaatct ggtggtatta ctgtttcaca tttgagattt
16741 ggtgacagac caattaattc tccatacttg atccatagag ctgatttcat ctcttgttca
16801 caacaatctt atgttgaaag atacgatttg ttggatggtt tgaaaccagg tggtacattt
16861 ttattgaatt gttcttggtc agatgcagaa ttagaacaac atttgccagt tggttttaaa
16921 agatatttgg ctagagaaaa cattcatttt tacactttga atgcagttga tattgctaga

16981 gaattaggtt tgggtggtag attcaatatg ttgatgcaag ctgctttctt taaattggct
17041 gcaattattg atccacaaac tgctgctgat tatttgaagc aagctgttga aaaatcatac
17101 ggttctaaag gtgctgctgt tattgaaatg aaccaaagag caatcgaatt gggtatggct
17161 tcattgcatc aagttacaat tccagcacat tgggctactt tggatgaacc agctgcacaa
17221 gcatctgcta tgatgccaga tttcatcaga gatatcttac aaccaatgaa tagacaatgt
17281 ggtgaccaat tgccagtttc agcttttgtt ggtatggaag atggtacatt tccatctggt
17341 actgctgcat gggaaaagag aggtattgct ttggaagttc cagtttggca accagaaggt
17401 tgtactcaat gtaaccaatg tgcttttatt tgtccacatg ctgcaattag accagcttta
17461 ttgaatggtg aagaacatga tgctgcacca gttggtttat tgtcaaaacc agcacaaggt
17521 gctaaggaat accattacca tttggctatc tcaccattgg attgttctgg ttgtggtaat
17581 tgtgttgata tttgtccagc tagaggtaaa gcattgaaaa tgcaatcatt ggattctcaa
17641 agacaaatgg ctccagtttg ggattatgca ttagctttga caccaaagtc taaccctttt
17701 agaaaaacta ctgttaaggg ttcacaattc gaaactccat tattggaatt ttctggtgca
17761 tgtgctggtt gtggtgaaac accatacgct agattgatca ctcaattgtt cggtgacaga
17821 atgttaattg caaatgctac aggttgttct tcaatttggg gtgcatcagc tccatctatt
17881 ccatacacaa ctaatcatag aggtcatggt ccagcatggg ctaattcatt gttcgaagat
17941 aacgctgaat ttggtttagg catgatgttg ggtggtcaag cagttagaca acaaattgct
18001 gatgatatga cagctgcatt agctttgcca gtttcagatg aattatctga tgcaatgaga
18061 caatggttgg ctaaacaaga tgaaggtgaa ggtactagag aaagagctga tagattatct
18121 gaaagattgg ctgctgaaaa agaaggtgtt ccattattgg aacaattgtg gcaaaacaga
18181 gattacttcg ttagaagatc acaatggatc tttggtggtg acggttgggc atacgatatt
18241 ggttttggtg gtttagatca tgttttggct tctggtgaag atgttaacat cttggttttt
18301 gatacagaag tttattcaaa tactggtggt caatcttcaa aatctacacc agttgctgca
18361 attgcaaaat ttgctgcaca aggtaaaaga actagaaaga aagatttggg tatgatggct
18421 atgtcttatg gtaatgttta cgttgcacaa gtcgctatgg gtgcagataa agatcaaact
18481 ttgagagcaa ttgctgaagc agaagcatgg ccaggtccat cattagttat tgcttatgct
18541 gcatgtatca atcatggttt gaaagctggt atgagatgtt ctcaaagaga agctaaaaga
18601 gcagttgaag ctggttattg gcatttgtgg agataccatc cacaaagaga agctgaaggt
18661 aaaacacctt ttatgttgga ttcagaagaa ccagaagaat cttttagaga tttcttgttg
18721 ggtgaagtta gatacgcttc attacataag acaactccac atttggcaga tgctttgttt
18781 tcaagaactg aagaagatgc aagagctaga tttgcacaat acagaagatt ggctggtgaa
18841 gaagctagag gggggggtag acgtgctttt catactcgtg ggggaggtcg tcgtgccttt
18901 catactatgg aaagggtctt aattaatgac actacattgc gtgatggtga acagtctcca
18961 ggagtcgcat ttagaacgag cgagaaggta gccatcgctg aggctttata tgctgccggt
19021 ataaccgcaa tggaagttgg cacacctgca atgggagatg aagaaatagc taggatacag
19081 cttgttagaa ggcaactacc tgacgctacc ctaatgactt ggtgtagaat gaacgccctg
19141 gagataagac agtctgcaga cttaggaata gattgggttg acatttccat cccagcttca
19201 gataagttgc gtcaatacaa gttgagagaa cctttggcag ttttgctgga gagattagca
19261 atgtttattc atttagccca tacactgggc ttaaaggtct gtattggttg tgaggatgca
19321 tcaagagctt ctggacaaac tttgagggct attgctgaag ttgctcagca atgtgctgca
19381 gctagactta gatacgcaga tactgtagga ctactagatc cattcactac cgcagcccaa
19441 atttcggctc ttagggatgt ttggtctgga gaaattgaaa tgcatgctca taacgattta
19501 ggaatggcta ctgctaacac tcttgccgca gtgtcagccg gtgctacaag tgtaaacaca
19561 accgttctgg gcctgggcga gagagccggt aatgctgctt tggaaactgt tgccctagga
19621 ttagagagat gtttaggagt ggagactgga gtacacttct cggctctacc tgcttcttgc

19681 caaagagtgg ctgaagctgc ccaaagagct attgatcctc aacaaccctt agtgggtgaa
19741 cttgttttta cacacgaatc cggtgtacat gtagcagctt tgttaagaca ctcagagtct
19801 taccaaagta ttgcaccttc attaatggga agatcgtata ggttggttct tggcaaacat
19861 tctggtagac aagctgttaa cggtgtgttc gatcagatgg gttaccactt aaatgcagct
19921 caaattaacc aattgttacc agctatcagg agattcgcag aaaattggaa gcgttctcca
19981 aaggattacg aattggtcgc aatttatgac gaattgtgtg gtgaatcagc cctgagagct
20041 agaggtgctc gtggaggggg cagaagagcg ttccatacga gaggtggcgg tagaagggca
20101 tttcatacaa tgatggaatg gttctaccaa atcccaggtg ttgatgaatt gagatctgct
20161 gaatctttct ttcaattttt cgcagttcca taccaaccag aattgttagg tagatgttct
20221 ttgccagttt tggctacatt ccatagaaag ttgagagcag aagttccatt gcaaaacaga
20281 ttggaagata acgatagagc accatggttg ttagctagaa gattgttagc agaatcttac
20341 caacaacaat tccaagaatc aggtacttaa gcaggcccct tttcctttgt cgatatcatg
20401 taattagtta tgtcacgctt acattcacgc cctcctccca catccgctct aaccgaaaag
20461 gaaggagtta gacaacctga agtctaggtc cctatttatt tttttaata gttatgttag
20521 tattaagaac gttatttata tttcaaattt ttcttttttt tctgtacaaa cgcgtgtacg
20581 catgtaacag ggcccgcttt atataatttg aaataaataa accgttcgct acctggagag
20641 acgcgcccgc tgatcctttg cgaatacgcc cacgcgatgg gtaacagtct tggcggtttc
20701 gctaaatact ggcaggcgtt tcgtcagtat ccccgtttac agggcggctt cgtctgggac
20761 tgggtggatc agtcgctgat tagcgattga ttactcccaa gatatataag gtttattttt
20821 atttttgttt gatttatccc tttcgctata ttcaaattat agtagaaagt gttgtcacca
20881 cattgcaatt caagctatcc tatatttaaa caatttctat tcttacttat atgccatgaa
20941 ggatttcaat aaccccagt atcactacca ataaaggaaa gcaatattta atagctttta
21001 ctactatatg gttagctgct cactactact ttgcaatgtt gaagtacgga ttactaaccg
21061 ccgaccggat gacagcactc cggcggaata ctcttttccg tgcgtcaccg tctgcgccgg
21121 tcgtgcccct gaactcagat gtgtctcgca ccgccatgct ccggacaatg caaaagtagg
21181 aagaggtgcc tgtcgtgatg gttatagtga atgcaaaaga cgattcccct ggccccgcat
21241 actttgcact cggtacaatg cattgattag cataatgata ataatacgat tgaggggggtt
21301 tcctcaccat tgtaatagtt gcaacataaa gtggtggttt tcaatatctt gacaaacata
21361 taaatgcaaa tgtaatattg attaaggtac tcagtttttt aatcattccc tctttatagt
21421 tttatcactt ctagttccac actactatga gctaagaaag agatcaagag caaatcttta
21481 atatccttcg ggatactatt tatttaggga aatatacaac tacaatggct tccactcgtg
21541 tcctggcctc tcgcctggcc tcgcagatgg ctgcttccgc caaagttgcc cgccctgctg
21601 tccgcgttgc tcaggtgagc aagcgcacca tccagactgg ctcgcccctc cagaccctca
21661 agcgcaccca gatgacttcc atcgtcaacg ccaccacccg ccaagctttc cagaagcgcg
21721 cctactcttc gatgaagggt aatgagattc ttgccttatt agatgaacct gcctgcgaac
21781 acaaccataa acaaaagtca ggttgttcgg caccaaagcc cggtgccact gccgccggtt
21841 gtgctttcga cggtgctcag atcacacttc ttcccattgc agatgtagcc catttggtcc
21901 acggtcccat cggatgtgct ggttcttcat gggacaacag aggttctgct tcctcgggcc
21961 caactttaaa tagacttgga tttacaacag atcttaatga acaagacgtt attatgggta
22021 gaggtgaaag aagattgttt cacgcagtta gacacattgt tactagatac catccagcag
22081 cagtttttat atataacaca tgtgtgccag ctatggaggg tgatgatttg gaagctgttt
22141 gccaagctgc tcagactgct actggtgttc cagtgattgc catagacgct gccggctttt
22201 atggtagtaa aaatctagga aacagacctg ctggtgatgt tatggtcaaa agagtcatcg
22261 gccaaagaga gccagctcct tggccagaat ctactctatt cgctcctgaa caaaggcatg
22321 atattggtct tattggtgaa tttaatatcg ctggtgagtt ctggcacatt cagccactgt

22381 tagacgaatt aggcatcaga gttcttggtt ccctttctgg tgatggtaga tttgctgaaa
22441 ttcaaactat gcatagagcc caagctaaca tgttggtatg ttccagagca ttgattaatg
22501 tcgcaagagc attagaacaa agatacggta caccttggtt cgaaggctct ttttacggta
22561 ttagagccac atccgacgca ttaagacaac ttgcagctct attgggtgat gatgacttaa
22621 gacaaagaac tgaagccttg attgctagag aagaacaagc tgctgagcta gcattacaac
22681 cttggaggga acaattgaga ggaaggaaag ccttgctata cacaggtggt gttaagtcat
22741 ggtccgtggt ttccgctttg caagaccttg gtatgaccgt tgttgctaca ggtacgagaa
22801 agagtactga agaggacaag cagagaatca gagagctgat gggtgaagaa gcagttatgt
22861 tggaagaagg aaacgctagg accttgctag acgttgtata caggtatcaa gccgatttga
22921 tgatagcagg tggtaggaat atgtacacag catataaagc aaggttacca ttcttggaca
22981 tcaatcaaga aagagaacac gctttcgctg gataccaagg tatagtcact ttggctagac
23041 agctatgtca aaccattaat tcccctatct ggccacaaac tcactccaga gccccctgga
23101 gagcaagagg aggtggtcgt agggcctttt ctactagagg aggaggtagg agagcctttt
23161 ccacaatggc cgatattttt agaactgaca agccattggc cgtatctcca atcaagaccg
23221 gacaaccatt gggcgccatc ttagcttcct taggcataga acattctatt cctttggttc
23281 atggtgctca gggatgttct gcatttgcaa aagttttttt tatacagcac tttcacgacc
23341 cagttccact tcaatcgaca gcaatggacc ctacgtccac tatcatgggt gcagatggaa
23401 atatctttac agcattagat accttgtgcc aaagaaacaa cccacaagcc atcgtattgc
23461 taagtactgg cttaagtgaa gcccaaggtt ccgacatatc aagagtagtc agacaattca
23521 gggaagaata ccctaggcac aagggtgtag ctattttgac tgtaaatact ccagacttct
23581 atggttcgat ggagaatgga ttttcggctg ttcttgaatc tgtcatcgaa caatgggtcc
23641 ctcccgcacc aagaccagct cagagaaata gaagagttaa tctattagtc agtcatttat
23701 gctcaccagg tgacattgag tggctaagaa gatgcgtaga agcctttggc ttgcaaccaa
23761 ttatccttcc agatttggct caatctatgg atggtcactt agctcaaggt gatttttctc
23821 ccttgacgca aggaggtaca ccacttagac aaatagaaca aatggacaa tcactatgta
23881 gctttgccat cggtgtatct ttgcacagag cctcttcttt gttggctcca agatgtagag
23941 gtgaagttat tgctctgcct catcttatga ctttggagag atgtgatgct tttatccatc
24001 agttagccaa aatcagtggt agagccgtgc cagagtggct agaaaggcag agaggtcaat
24061 tacaagacgc tatgattgat tgtcatatgt ggttgcaagg tcagagaatg gctattgccg
24121 cagagggtga ccttcttgca gcctggtgtg actttgccaa ctctcaagga atgcaacccg
24181 gtccactggt agcaccaaca ggacacccctt cccttagaca attgccagtt gaaagagttg
24241 ttcctggtga cctagaagat cttcagacct tattatgtgc tcacccagca gatcttttgg
24301 tagcaaatag tcatgccaga gacttggcag aacagttcgc tcttccactg gtgagagctg
24361 gttttccatt gtttgataaa ttgggtgagt tcagaagagt gagacaaggt tattctggta
24421 tgagagatac gttgttcgag ttggcaaatt tgattagaga aagacatcac cacttggcac
24481 attatagatc ccctttaaga caaaatccag aatccagtct ttccactggt ggtgcatatg
24541 ctgcagatgg tggaggtggt tctggaggtg gtggatcagg tggtggaggt agtacaagtt
24601 gtttatcttt ctctggtggc aaagcctgta gacctgctga tgattcagca ttaacccctc
24661 ttgttgcaga taaggctgct gcacatccat gttactccag acatggacac catagatttg
24721 ctaggatgca tttaccagtg gctccagctt gtaatctaca gtgcaattac tgcaatagaa
24781 aattcgactg ctccaatgaa tcaagacctg gcgtgagtag caccttactt acccctgaac
24841 aggctgtggt caaggtaaga caggttgcac aggcaattcc acaactttct gttgtgggaa
24901 ttgccggacc tggcgatcct ttagccaata tcgctagaac cttcaggact ttggagttga
24961 ttagagaaca attgccagat cttaagctgt gtttatcaac taatggatta gttttaccag
25021 atgccgtgga taggttgttg gacgtgggtg ttgaccacgt taccgttacg atcaacacgt

25081 tggatgctga aattgctgct caaatttatg cttggctttg gttagatggc gaaagatatt
25141 caggaagaga agctggagaa attttgattg ctaggcaatt agagggagtt aggagactta
25201 ctgcaaaagg cgtgttggtt aagataaact ctgttttgat tccaggtatt aacgattcag
25261 gtatggccgg tgtctccaga gcattgagag cttccggtgc ctttattcac aatattatgc
25321 cattaattgc tagaccagaa cacggcactg tctttggtct gaacggtcaa cctgaaccag
25381 acgcagaaac tttggctgca accagatcga gatgtggaga agtcatgcca caaatgaccc
25441 attgtcacca atgtagggcc gacgctattg gcatgctggg agaagatagg agtcagcaat
25501 ttacgcaatt gcctgctcca gaatctctac ccgcttggtt gcctatatta catcagagag
25561 cacaactgca tgcttctata gcaacaagag gtgaatcaga agcagacgac gcctgcctag
25621 ttgctgttgc atcatcaaga ggcgacgtca tcgattgcca tttcggacat gcagacagat
25681 tttacatata ttcccttagt gctgctggca tggtcctagt taatgaaaga ttcacaccaa
25741 aatattgtca aggtagagat gattgtgagc ctcaagataa cgcagctaga tttgctgcca
25801 ttctggagct actggctgat gttaaagctg ttttctgtgt taggatcggt catactccct
25861 ggcaacagct tgagcaagaa ggtattgagc catgtgtaga cggagcatgg aggccagtta
25921 gcgaggtatt gcctgcatgg tggcagcaga ggagaggtag ctggccagcc gctttaccac
25981 acaagggcgt cgcctaagct gcaactcctc aatgtgtcaa ttaactctta cttaatttat
26041 gtatatattt tttatgtata tgcttatata catgcgcata tgttcataaa agatacattg
26101 ttataggtcc tttcttttcc aagctacatc tagcgcttta tataatttgg gactaacaaa
26161 gggctttgtt cgtcccaatc aacttatgct tgcgtcttct gcctacgcaa cactttagct
26221 gcttttttta agcagcggca gatgaggatt taaataacgc ataaccaaga atatacctaa
26281 tgaagtgaag gtatacaggc tgttctatgg acttgaataa tactggctac cctggcgtcc
26341 taatgtcgtt aagaaagtaa cttgatatca gattaatcac aaacttggtc cacaaaatac
26401 cacttatctc atataatcta aatctgacta aagggtgtag gcacggaact tcggacctag
26461 aggtattgct caatatgtat atttctcaat ggttcatcta ggtaagcaag gccttgaata
26521 atatacttaa tcttgtctgt ttcgcaacaa ttgctgagta caataatgtt ggaataaaaa
26581 tcaactatca tctactaact agtatttacg ttactagtat attatcatat acggtgttag
26641 aagatgacgc aaatgatgag aaatagtcat ctaaattagt ggaagctgaa acgcaaggat
26701 tgataatgta ataggatcaa tgaatattaa catataaaat gatgataata atatttatag
26761 aattgtgtag aattgcagat tcccttttat ggattcctaa atcctcgagg agaacttcta
26821 gtatatctac atacctaata ttatagcctt aatcacaatg gaatcccaac aatctagaga
26881 agatcctttg atcttttcta cggggtctga cgctcagtgg aacgaaaact cacgttaagg
26941 gattttggtc atgaacaata aaactgtctg cttacataaa cagtaataca aggggtgtta
27001 tgagccatat tcaacgggaa acgtcttgct ctaggccgcg attaaattcc aacatggatg
27061 ctgatttata tgggtataaa tgggctcgcg ataatgtcgg gcaatcaggt gcgacaatct
27121 atcgattgta tgggaagccc gatgcgccag agttgtttct gaaacatggc aaaggtagcg
27181 ttgccaatga tgttacagat gagatggtca gactaaactg gctgacggaa tttatgcctc
27241 ttccgaccat caagcatttt atccgtactc ctgatgatgc atggttactc accactgcga
27301 tccccgggaa aacagcattc caggtattag aagaatatcc tgattcaggt gaaaatattg
27361 ttgatgcgct ggcagtgttc ctgcgccggt tgcattcgat tcctgtttgt aattgtcctt
27421 ttaacagcga tcgcgtattt cgtctcgctc aggcgcaatc acgaatgaat aacggtttgg
27481 ttgatgcgag tgattttgat gacgagcgta atggctggcc tgttgaacaa gtctggaaag
27541 aaatgcataa acttttgcca ttctcaccgg attcagtcgt cactcatggt gatttctcac
27601 ttgataacct tatttttgac gaggggaaat taataggttg tattgatgtt ggacgagtcg
27661 gaatcgcaga ccgataccag gatcttgcca tcctatggaa ctgcctcggt gagttttctc
27721 cttcattaca gaaacggctt tttcaaaaat atggtattga taatcctgat atgaataaat

```
27781 tgcagtttca tttgatgctc gatgagtttt tctaagaatt aattcatgag cggatacata
27841 tttgaatgta tttagaaaaa taaacaaata ggggttccgc gcacatttcc ccgaaaagtg
27901 ccacctgaga tcaaaggatc ttcttgagat cctttttttc tgcgcgtaat ctgctgcttg
27961 caaacaaaaa aaccaccgct accagcggtg gtttgtttgc cggatcaaga gctaccaact
28021 ctttttccga aggtaactgg cttcagcaga gcgcagatac caaatactgt tcttctagtg
28081 tagccgtagt taggccacca cttcaagaac tctgtagcac cgcctacata cctcgctctg
28141 ctaatcctgt taccagtggc tgctgccagt ggcgataagt cgtgtcttac cgggttggac
28201 tcaagacgat agttaccgga taaggcgcag cggtcgggct gaacgggggg ttcgtgcaca
28261 cagcccagct tggagcgaac gacctacacc gaactgagat acctacagcg tgagctatga
28321 gaaagcgcca cgcttcccga agggagaaag gcggacaggt atccggtaag cggcagggtc
28381 ggaacaggag agcgcacgag ggagcttcca gggggaaacg cctggtatct ttatagtcct
28441 gtcgggtttc gccacctctg acttgagcgt cgatttttgt gatgctcgtc aggggggcgg
28501 agcctatgga aaaacgccag caacgcggcc tttttacggt tcctggcctt ttgctggcct
28561 tttgctcaca tgttctttcc tg
//
```
